# A Semi-automated and Scalable 3D Spheroid Assay to Study Neuroblast Migration

**DOI:** 10.1016/j.stemcr.2020.07.012

**Published:** 2020-08-06

**Authors:** Martin Ducker, Valerie Millar, Daniel Ebner, Francis G. Szele

**Affiliations:** 1 Department of Physiology, Anatomy and Genetics, University of Oxford, South Parks Road, Oxford OX1 3QX, UK; 2 Target Discovery Institute, Nuffield Department of Medicine, University of Oxford, Oxford, UK

**Keywords:** migration, neuroblasts, 3D, assay, drug discovery, subventricular zone, imaging, screen, cancer, regenerative medicine

## Abstract

The subventricular zone of the mammalian brain is the major source of adult born neurons. These neuroblasts normally migrate long distances to the olfactory bulbs but can be re-routed to locations of injury and promote neuroregeneration. Mechanistic understanding and pharmacological targets regulating neuroblast migration is sparse. Furthermore, lack of migration assays limits development of pharmaceutical interventions targeting neuroblast recruitment. We therefore developed a physiologically relevant 3D neuroblast spheroid migration assay that permits the investigation of large numbers of interventions. To verify the assay, 1,012 kinase inhibitors were screened for their effects on migration. Several induced significant increases or decreases in migration. *MuSK* and *PIK3CB* were selected as putative targets and their knockdown validated increased neuroblast migration. Thus, compounds identified through this assay system could be explored for their potential in augmenting neuroblast recruitment to sites of injury for neuroregeneration, or for decreasing malignant invasion.

## Highlights

• No accessible screening assay exists for neuroblast migration
• We have developed a simple and robust assay for measuring neuroblast migration
• The assay is suitable for functional or screening studies
• We used a library of kinase inhibitors and several increased or decreased migration

## Graphical Abstract

**Figure:**
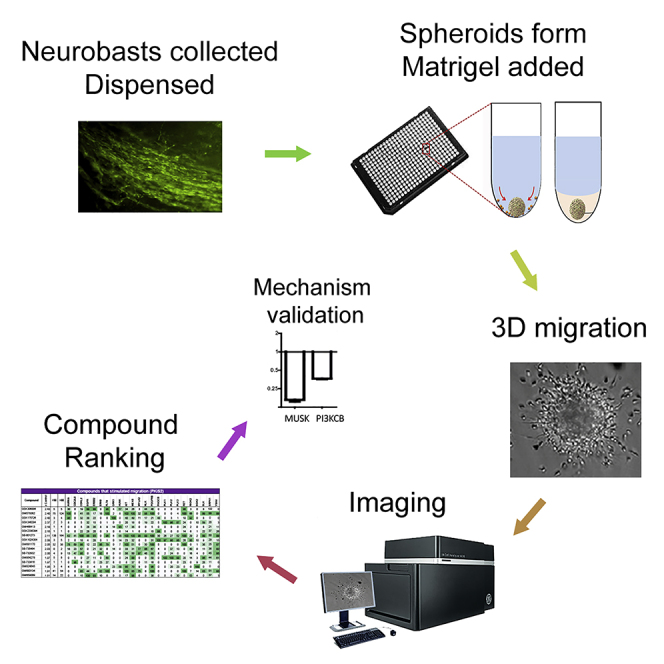

## Teaser

Cell migration is essential in development and regeneration and contributes to cancer pathology. Here, Szele and colleagues have developed and used a fast, easy, and robust assay for neuroblast migration. The assay is versatile, with medium throughput and was used to screen a kinase inhibitor library. They found and validated that MuSK and PIK3CB are necessary for neuroblast migration.

## Introduction

Neurogenesis is not a single event but a complex multistage process encompassing the proliferation of neural stem cells, their survival, migration, differentiation, and functional integration into the developed neuronal circuitry (Ming and Song, 2005). The rodent subventricular zone (SVZ) lies next to ependymal cells lining the wall of the lateral ventricles. Adjacent to the ependymal cells are quiescent stem cells that occasionally divide symmetrically to produce two transit amplifying progenitors, which divide several times before giving rise to highly motile neuroblasts. Neuroblasts characteristically express the polysialylated form of the neural cell adhesion molecule and the early neuronal marker doublecortin (DCX) (Alvarez-Buylla and Garcia-Verdugo, 2002). Neuroblasts in the rodent brain initiate the migratory route by establishing chains of migration along the rostral migratory stream (RMS), the route to the olfactory bulb (OB). The cells detach from the RMS in the OB, migrate radially to the outer layers, and terminally differentiate into different subtypes of interneurons, with six independent cell types characterized, dependent on their origin along the axes of the SVZ (Merkle et al., 2007). A few molecules, such as galectin-3 (Comte et al., 2011), DCX (Koizumi et al., 2006), and prokineticin (Ng et al., 2005) are known to mediate RMS neuroblast migration. Yet, overall little is known about mechanisms regulating migration compared with other functions of the SVZ.

Pharmacologically modulating the migration of neuroblasts has the potential to improve the prognosis of multiple neurological disorders (Chang et al., 2016; Young et al., 2011). Research into the process of neuronal migration and its regulatory mechanisms has evolved due to advances in molecular genetic technologies, allowing for the effects of individual genes on migration to be interrogated. This has relied on the development of assay platforms for neuronal migration, which have included organotypic slice cultures (Stoppini et al., 1991), transwell migration assays (Kramer et al., 2013), *ex vivo* explant assays (Leong et al., 2011), hanging drop assays (Falenta et al., 2013; Sonego et al., 2013), and 3D tumor spheroid assays (Vinci et al., 2015).

There is great potential in using phenotypic screening techniques to uncover mechanisms regulating neuroblast migration, but to date few successful studies have been published. Existing models for studying neuroblast migration have not been suitable to use in a high-throughput screen (HTS) due to the cost, labour-intensive design, or lack of reproducibility. There is considerable momentum in the drug discovery field to move from the reductionist 2D *in vitro* HTS platforms using immortalized cell lines to more physiologically relevant 3D models using primary cell types and 3D scaffolds for high-content screening. These models are far better mimics of complex tissue and adhere far better to a set of principles to facilitate the definition and development of disease-relevant assays (Hulkower and Herber, 2011). Building on established assay techniques, and taking these principles into consideration, we set out to develop a screening platform that was both physiologically relevant and highly reproducible.

Protein kinases have a vast array of roles in cellular activity, and have been strongly implicated in neuroblast migration. Several receptor tyrosine kinases are expressed by neuroblasts and have been shown to modulate migration, including the IGF1R, Eph receptors EPHA4 and EPHB1–3, PDGFR, and VEGFR (Hurtado-Chong et al., 2009; Ishii et al., 2008; Kim et al., 2010; Mani et al., 2009; Todd et al., 2017). Some of these were surprising as they had been associated with regulating proliferation. The EGFR, or ERBB1, is necessary for driving proliferation and is a marker of mitotic progenitors in the SVZ, but it is also negatively correlated with neuroblast motility (Kim et al., 2009). A large collection of intracellular kinases mediate neuronal migration by phosphorylating key components of signaling pathways, such as CDK5, GSK3β, MAPK-upstream protein kinase, members of c-Jun N-terminal kinase family, and extracellular signal-regulated kinase (Kawauchi, 2014; Simo et al., 2007; Hirai et al., 2002; Konno et al., 2005). However, small-molecule compounds that stimulate neural progenitor migration *in vivo* is still an open challenge in drug discovery.

Our main aim was the development of a neuroblast migration assay that is more physiologically relevant and therefore a better tool to investigate large numbers of interventions to identify new drug targets and starting molecules for drug development. Compounds that increase migration of neuroblasts could have therapeutic potential in stroke, traumatic brain injury, or neurodegenerative disorders; and compounds that decrease migration may be useful as anti-invasive treatments for brain cancer. Therefore, a reliable assay to identify such compounds could be a powerful tool in neuroscience research. The Published Kinase Inhibitor Set (PKIS1 and PKIS2), an annotated set of approximately 900 small-molecule kinase inhibitors, was developed by GlaxoSmithKline due to the abundance of uncharacterized kinases (Drewry et al., 2014). The PKIS library has previously been used to investigate the functions of kinases in various cell types and diseases, including in neuroregeneration (Al-Ali et al., 2015). Using the PKIS, we validated the potential for our 3D spheroid migration assay to identify compounds that significantly increase or decrease neuroblast migration.

## Results

### 3D RMS Spheroid Migration Assay

**Figure 1:**
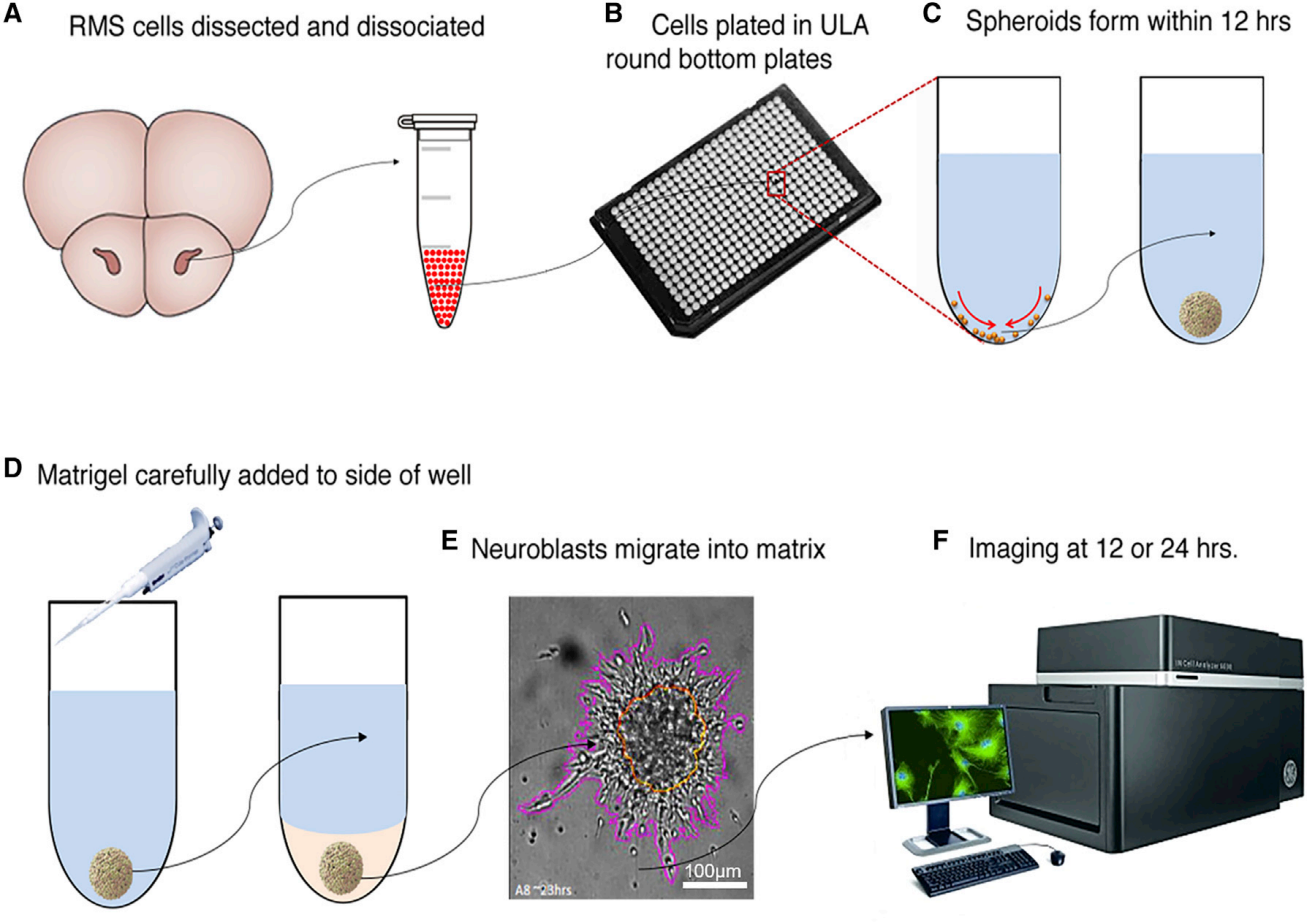
Schematic of Assay Developed for Neuroblast Migration (A) The RMS (dark pink) is dissected from slices and single-cell dissociated. (B and C) (B) Cells are plated in round bottom plates and (C) cells sink to bottom and form spheroids. (D and E) (D) Matrigel is added to the spheroids and (E) neuroblasts emigrate from them in 3D. The image shows the spheroid (encircled in yellow) at 24 h with the emigrated cells manually outlined in purple to determine emigration surface area. Scale bar, 100 μm. (F) Migration is imaged and measured at various time points.

Figure 1 shows the overall spheroid migration assay protocol. Neuroblast migration was studied by embedding the spheroids in Matrigel, which was previously reported to support SVZ cell migration *in vitro* (Azzarelli et al., 2017; Dizon et al., 2006) (Supplemental Results). Images of the spheroid we taken at 2, 24, and 48 h using an IN Cell Analyzer 6000 high-content image capture and analysis system (Figure 1). Figures S1–S3 show further details of the assay development.

Analysis of the frequency distribution in spheroid size indicated a Gaussian distribution, which was supported by a passed D'Agostino-Pearson omnibus normality test (p = 0.1813). The mean spheroid size was 27,437 ± 1,073 μm^2^ confirming that the Greiner ULA plates used can produce large quantities of reproducible RMS spheroids. The area containing migrating neuroblasts (Figure 1E) only very rarely extended out of the imaged field, averting the need for upper thresholds. Moreover, excellent optical properties of our spheroid-containing wells make them compatible with various types of readout devices, such as standard and lens-free microscopes, high-content imaging systems, and microplate readers.

### Application of Vital Dyes to 3D RMS Spheroids

**Figure 2:**
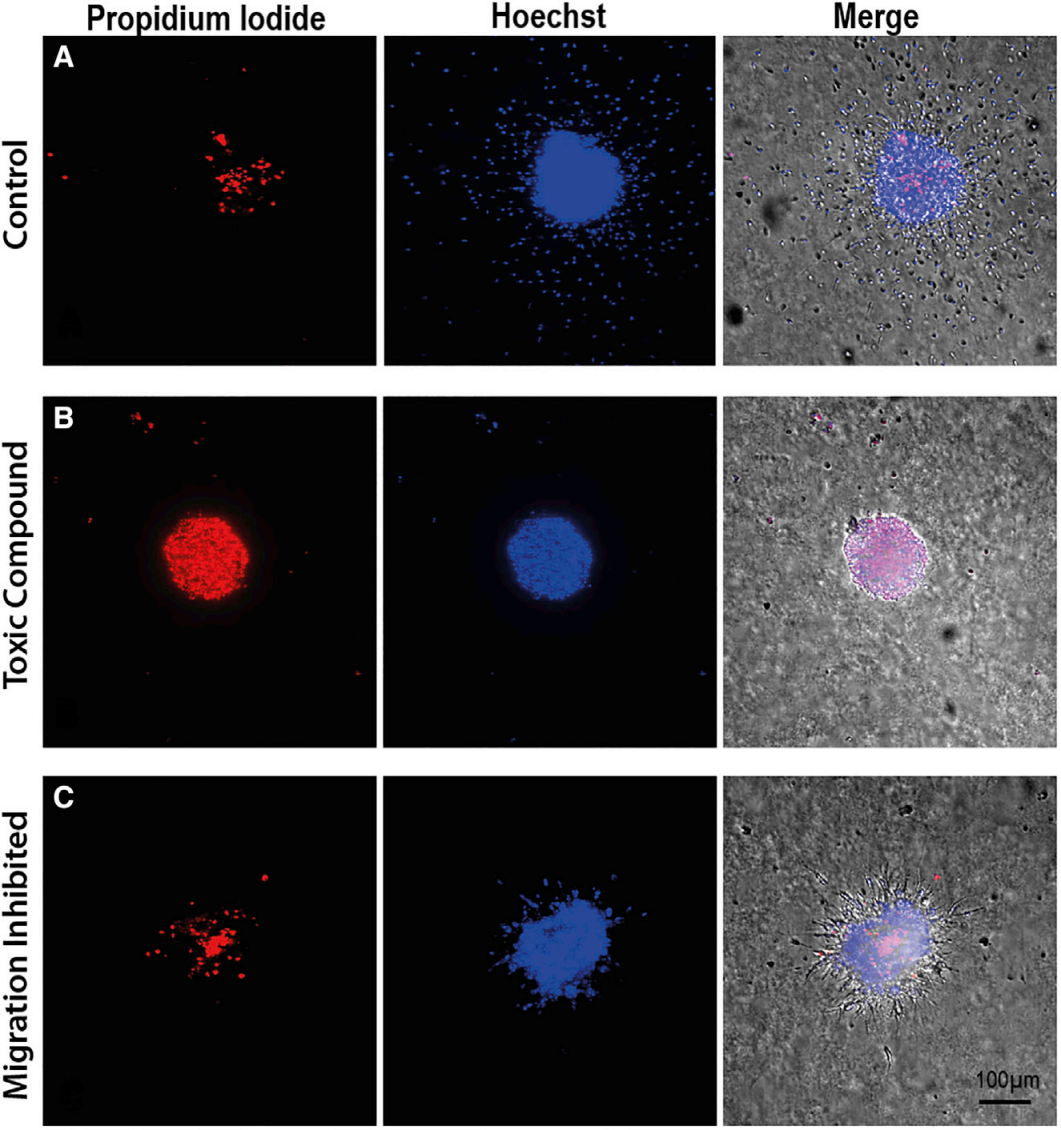
Spheroids Labeled with Fluorescent Dyes (A) Control neurosphere shows few propidium iodide+ (PI+) cells (dead, red) but many Hoechst+ cells (live, blue) in the core and that have emigrated. (B) A toxic compound (GW406731X) causes robust PI staining. Most cells died before they could emigrate. (C) A migration inhibitor (GSK1389063A) allows robust survival but little emigration compared with the control spheroid. The right-hand column shows merge of two fluorescent channels and bright field. Scale bar, 100 μm.

Cell viability and general spheroid health were measured using propidium iodide and Hoechst (Figure 2). The Hoechst dye was imaged using the standard DAPI filter set (361 nm/497 nm) and propidium iodide can be imaged using the standard Texas Red filter set (535 nm/617 nm). Both of these dyes bind to DNA, but while Hoechst is cell permeable, PI cannot cross the membrane of live cells. Therefore, the combination is useful to differentiate apoptotic and healthy cells in a culture. CellTracker Green was also shown to effectively label migrating spheroid cells (Figure S4).

### Image Acquisition

**Figure 3:**
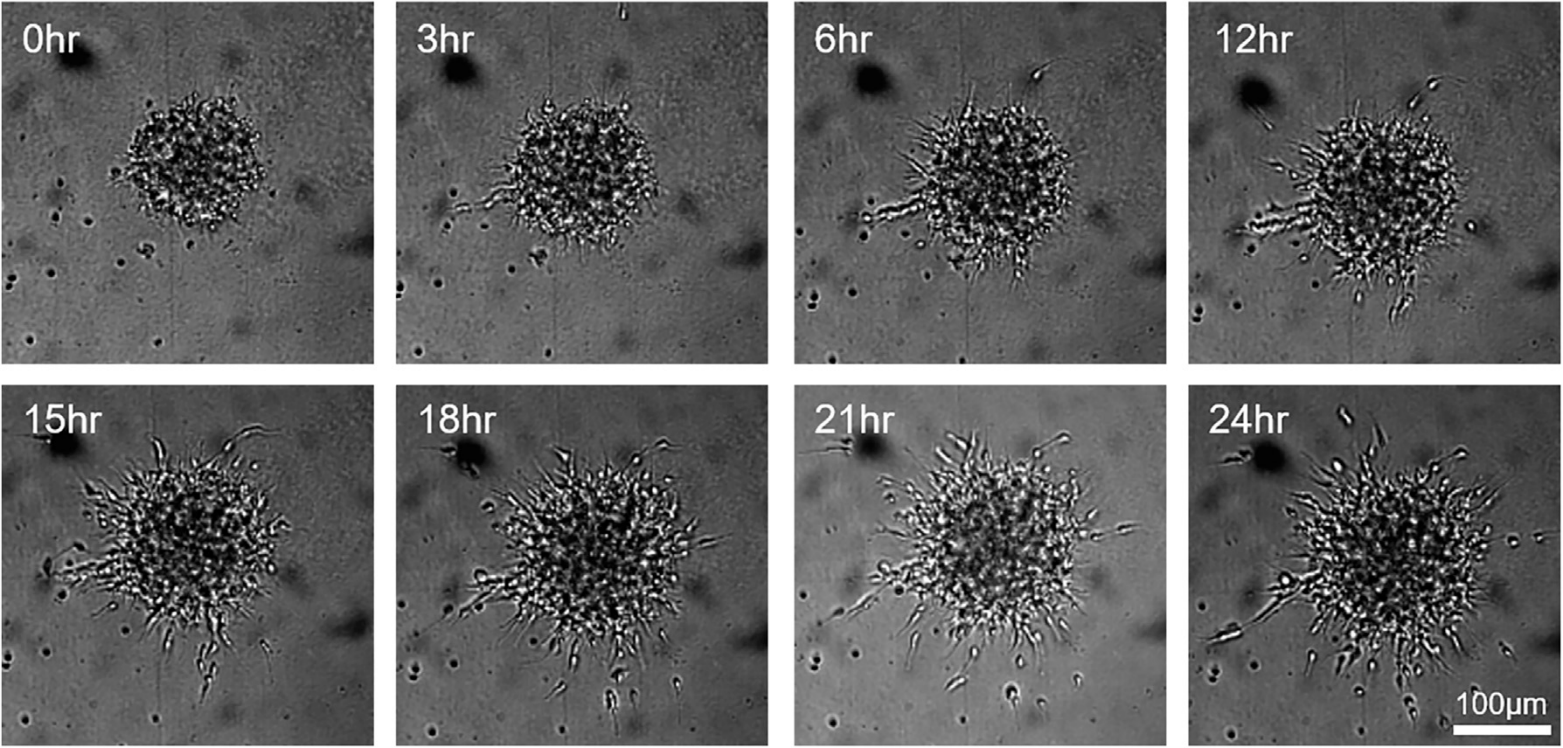
Time-Lapse Imaging of Spheroid Migration Using the GE IN Cell The IN Cell imaging platform took images over a 24-h period from plating of a spheroid in Matrigel. Migration of neuroblasts out of the spheroid could already be observed at 3 h and continued throughout the duration of the time lapse. Scale bar, 100 μm.

Uniform spheroids were established reproducibly and were robust enough for time-lapse microscopy. Imaging was primarily completed using the GE IN Cell Analyzer 6000, high-content imager (GE Healthcare). This system comprises both a bright-field and a line-scanning laser confocal microscope making it ideal for quick 3D cell imaging, in a high-content image format. A protocol was established on the IN Cell acquisition software that imaged the center of each well of the Greiner ULA plate. The starting plane was set by the microscope's built-in “software autofocus” function and nine optical sections were imaged in the z plane at intervals of 25 μm, which amounted to a total depth of 200 μm. Before starting the imaging, a trial scan was completed to check the position of the spheroid in each image. If any spheroid was dramatically off center or shifted in the z plane, the acquisition field was manually adjusted for that well. All scans included a bright-field image, taken above the well using an orange bright-field diode and a dsRed emission filter at an exposure of 0.2 s. When fluorescence was used, the exposure was set based on the individual signal level of that experiment. A single scan of the entire plate took 15 min on bright field and 25 min when using fluorescence channels. Images were taken every 30 min for 24 h (Figure 3).

Imaging was also carried out on three other systems, demonstrating versatility: (1) a Motic AE2000 inverted microscope with Moticam 580 digital camera, (2) an Essen Bioscience Incucyte, and (3) an EVOS FL Auto. For the Essen Bioscience Incucyte, the 10× objective was used, imaging in the center of the plate with the software's built-in autofocus.

### Manual Image Analysis

**Figure 4:**
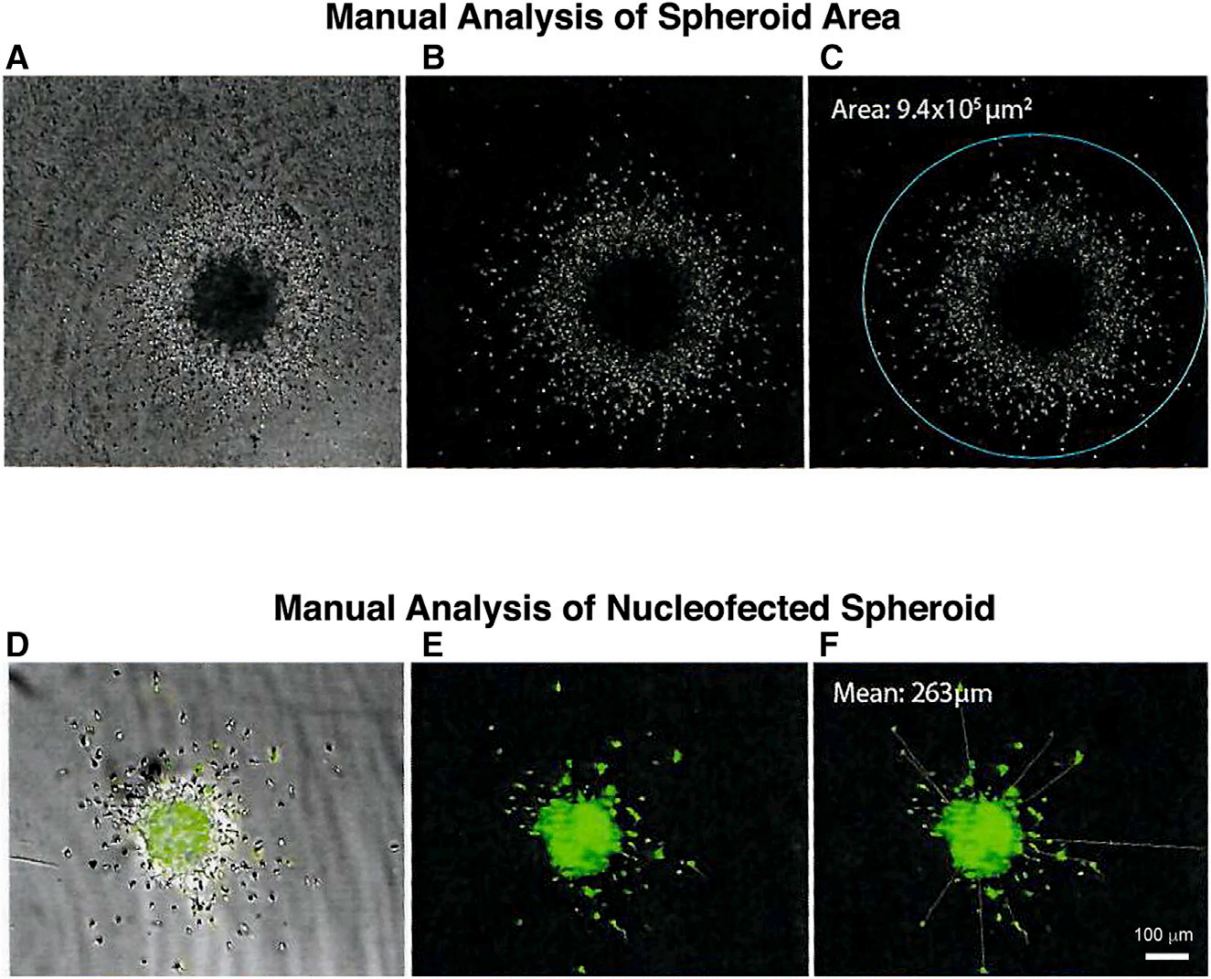
Images Showing the Workflow for Manually Quantifying Spheroid Images (A) Brightfield image of spheroid and emigrated cells. (B) A maximum intensity projection was created. (C) The area of an elipse encircling the migration was calculated. The "Find Edges" function was used as needed to improve the signal-to-noise ratio. (D) Overlap of brightfield and flouresence imaging of nucloefected cells. (E) Fluorescence imaging of nucleofected cells. (F) For manual analysis of nucleofected fluorescent cells, the average distance of the eight maximally migrated cells was measured. Scale bar, 100 microns.

To manually quantify migration of cells from spheroids, individual bright-field image stacks were loaded into ImageJ and a maximum intensity projection created (Figure 4). Unlike explants and hanging drop aggregates, we found that cell migration from spheroids is relatively evenly distributed and continued for up to 24 h after plating (Figure S5). This property was exploited to provide a quick and simple quantification method. An ellipse was drawn around the spheroid such that it satisfied the following criteria: (1) it was centered on the spheroid, (2) it intersected at least six cells deemed to have migrated from the spheroid, and (3) it was round (circularity >0.8). The area was then calculated and saved as a “region of interest.” In some instances, the “Find Edges” function was applied to assist in visualizing individual cells. When there were a few single cells that had migrated individually from the spheroid these were ignored so as not to skew the analysis (Figure 4). Any wells that were empty or in which the number of spheroids was greater than 1 were excluded. If the spheroid became misshapen or there was debris in the wells, they were excluded from analysis. Using these criteria, we excluded 4.2% of the wells.

Manual analysis of fluorescent nucleofected spheroids was completed by analysis of the average of the eight maximally migrated cells. The fluorescent channel image stacks were loaded into ImageJ and a maximum intensity projection created. The line function was then used to measure the distance from the spheroid edge to the ten cells that appeared furthest away making sure to save each as a region of interest. Only the top 8 of these measures were used for quantification.

### Automated Image Analysis

**Figure 5:**
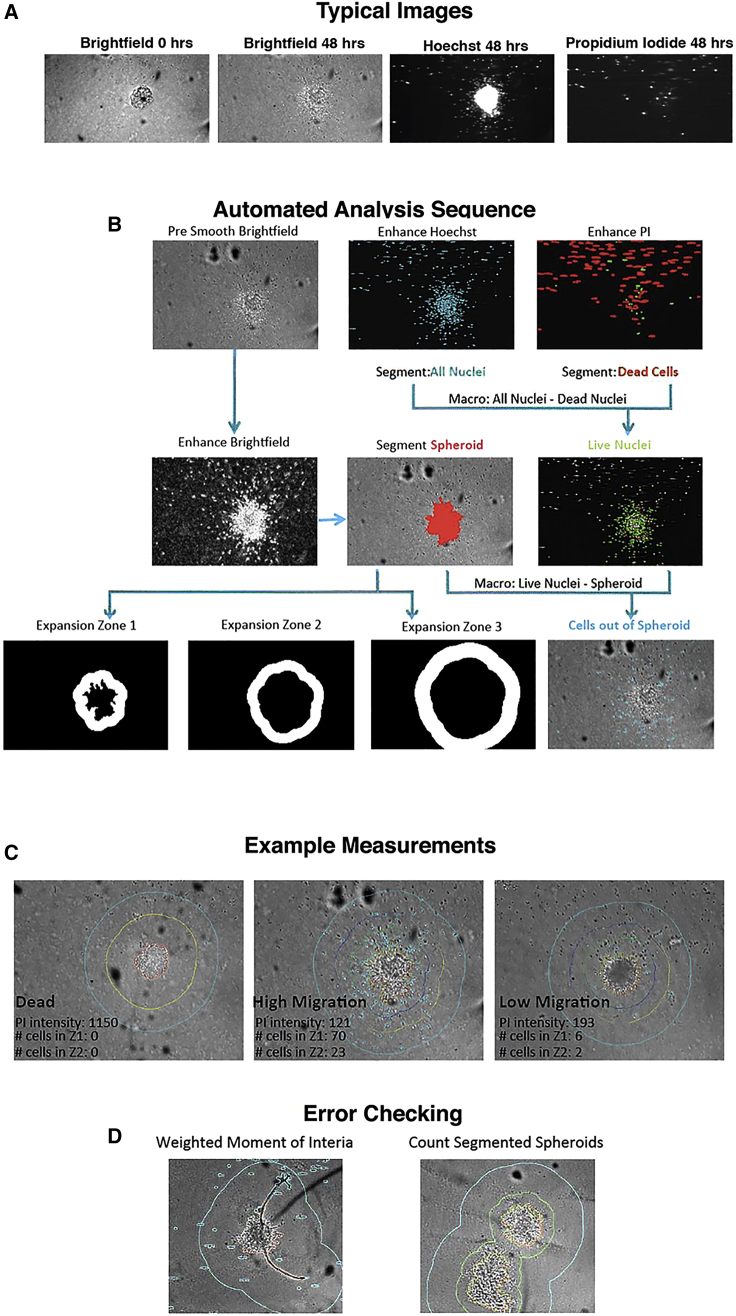
Workflow of Automated Spheroid Image Analysis (A) Examples of typical bright-field, Hoechst (live stain) and propidium iodide (dead stain) images used in our analysis. (B) The automated analysis sequence describes the imaging sequence and the processing carried out by the analysis software. (C) Neuroblast numbers within increasing radius zones from the spheroid were measured by the software to determine migration extent. (D) Errors affecting the counts were checked automatically.

Automated image analysis was carried out using a custom protocol developed in Developer Toolbox 1.9.2, GE IN Cell Analyzer 6000, high-content imager (Figure 5). To analyze collective cell migration from the spheroid, extended focus images were created from each bright-field image stack. A macro was written to apply the following functions to each image: Smooth > Find Edges > Dilation > Fill Holes > Erosion > Segment > Sieve. This resulted in segmentation of the spheroid and halo of migrated cells ready for quantification. To analyze the number and location of individual cells, fluorescence channels (Hoechst/propidium iodide) were used. First, extended focus projections were created for each image stack and the contrast enhanced. A macro was written to apply the following functions to each image: Segment > Fill Holes > Sieve. The Hoechst and propidium iodide images were then compared with identify live cells. With the central spheroid, halo, and all dead and alive cells segmented it is now possible extract features and quantify the migration. The area of the migration halo was calculated by subtracting the area of the spheroid core from the entire contiguous spheroid area. The halo area is a good measure of the extent of collective and chain migration from the spheroid. Similar to manual image analysis, two main exclusion criteria were used. The first was to omit wells where zero, two, or more spheroids were found and the second was to remove wells within which the spheroids were artificially misshaped due the incorporation of a fiber or other artifact. The upper adjacent value of the spheroid weighted moment of inertia, a measure of roundness, was used to identify misshapen spheroids. We also excluded cases of incorrect bright-field segmentation of the spheroid itself. Using these criteria a total of 8.5% of wells were excluded.

### Validation of the 3D Spheroid Assay through a Kinase Inhibitor Screen of SVZ Neuroblast Migration

PKIS1 and PKIS2 were openly available in screening quantities from the Structural Genomics Consortium, University of Oxford. Both libraries were requested at www.sgc-unc.org and chemical structures and pharmacological activity profiles for the compounds found at https://www.ebi.ac.uk/chembldb/extra/PKIS/compounds.html.

Six 96-well plates of 3D RMS spheroids, with each well containing 4,000 cells, were treated with 10-μM compound or DMSO as a control and returned to the incubator for 2 h to incubate. Plates were then imaged at 2 h (T = 0) and 24 h with bright field only. Two hours before the 48-h endpoint, the live/dead assay was added to each well and the plates were assessed with automated imaging of bright field and fluorescence.

Triplicate wells for each compound were normalized against the DMSO controls, and cutoffs for “high migration” or “low migration” compounds introduced, as those producing a migration area at least 4 standard deviations greater or lesser, respectively, from the mean of the controls. For the low-migration compounds, toxicity was excluded with the live/dead assay. This experiment yielded 24 compounds that elicited high migration areas in the screen ranging from 1.7× to 3.0× that of controls, and 36 compounds that decreased migration, with areas ranging from 0.17× to 0.35× that of controls (Tables S1–S3). In total, 181 compounds were deemed toxic based on qualitative evaluation of cell death compared with controls.

The 60 compounds selected as putative modulators of migration were then triaged into a dose-response confirmation experiment. Each compound was tested in triplicate at concentrations up to 10 μM, and dose-response curves were made to identify the concentration that produces the greatest migration phenotype (Tables S4 and S5). The intended targets for which the compounds were designed are also listed in Tables S4 and S5. Table S4 shows the 21 compounds out of the 24 tested that significantly increased migration at the maximum 10 μM concentration. Representative images of spheroid migration and the compound structures are indicated. The profile of the dose-response curves varied across the hit compounds with some causing significant increases in migration at the lower concentrations (GW607049C, GW459135A, GSK2336394A, GW579362A, and GSK1024304A), while others increased migration only at the highest concentration (GW693881A and GW854278X). The initial screen was performed blind: the primary kinase targets of the small-molecule inhibitor hits were unknown at the time of treatments and active compounds were selected solely based on migration area. Table S5 shows the compounds that significantly reduced migration area in comparison with DMSO. All 36 compounds tested significantly reduced neuroblast migration at the highest concentration of 10 μM.

**Figure 6:**
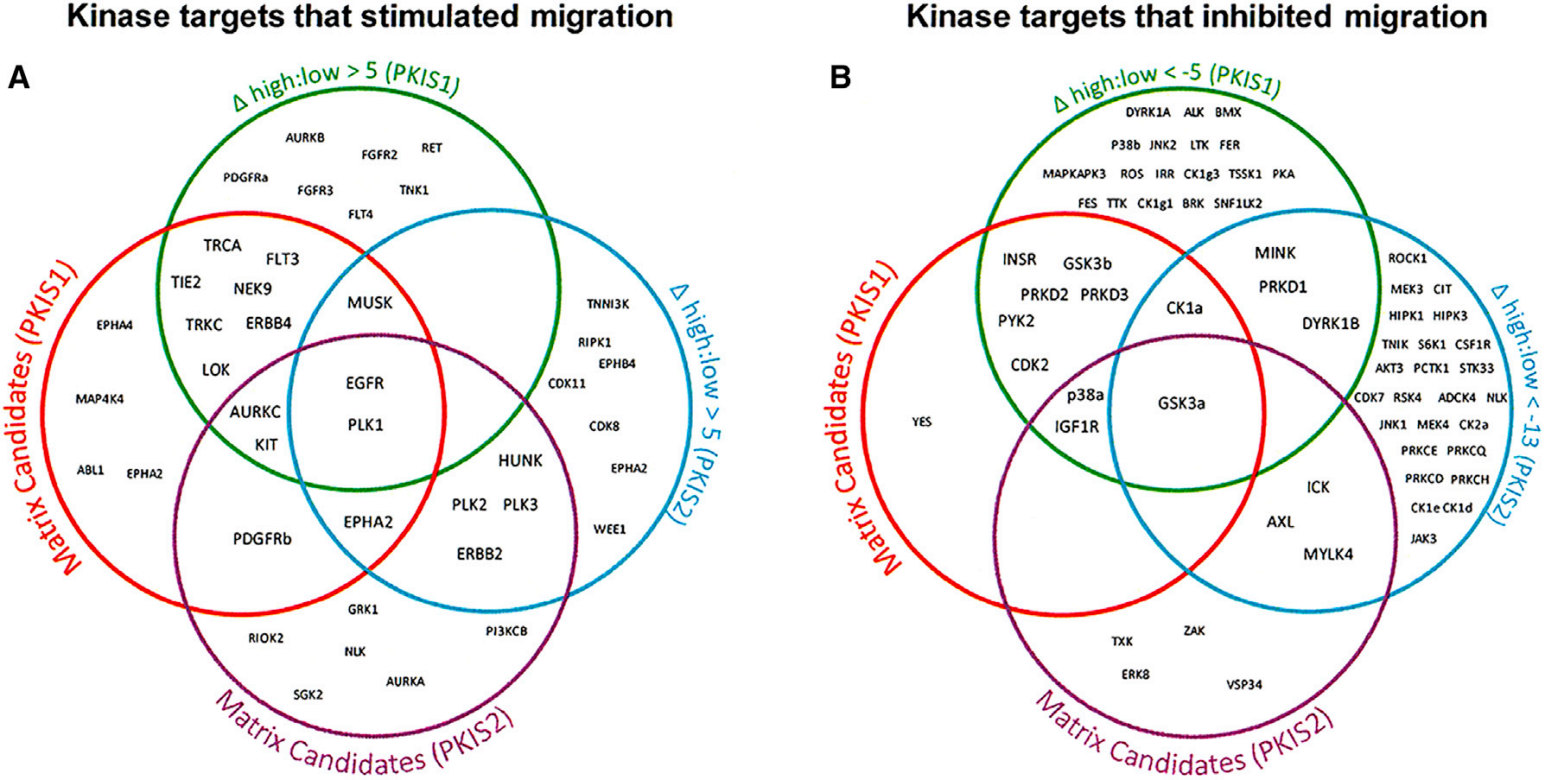
Comparison of Kinase Targets Selected by Analysis of the PKIS1 and PKIS2 Datasets Venn diagrams of the putative kinase targets selected by the analysis of the kinase targets for the “hit” compounds and the relative kinase affinity to compounds increasing (A) or decreasing (B) migration for both PKIS1 and PKIS2.

The lists of putative hits selected from each analysis method were then compared Figure 6. Please see Supplemental Results for a detailed description of criteria for hit selection in Figure 6. Several receptor tyrosine kinase targets were selected as inducing robust migration when inhibited. These included EGFR, ERBB2, ERBB4, EPHA2, TRKA, TRKC, PDGFR, TIE2, FLT3, and KIT. Other kinases selected included four members of the Polo-like kinase (PLK) family: PLK1, PLK2, PLK3, and LOK; NimA-related protein kinase 9; hormonally upregulated Neu-associated kinase; muscle-specific kinase (MuSK); and Aurora C kinase. Tyrosine kinase targets that reduced migration when inhibited included GSK3α, GSK3β, MAPK14/P38α, IGF1R, IR, CDK2, protein kinase D1, casein kinase 1, and dual-specificity Yak 1-related kinase 1B. Details of the active compound's binding ratios and relative kinase affinities are provided in Table S1. Figure S6 shows STRING representations of kinase targets that increased and decreased migration in the PKIS screens.

### Confirmation Studies Inhibiting MuSK and PIK3CB Promote the Migration of Neuroblast

**Figure 7:**
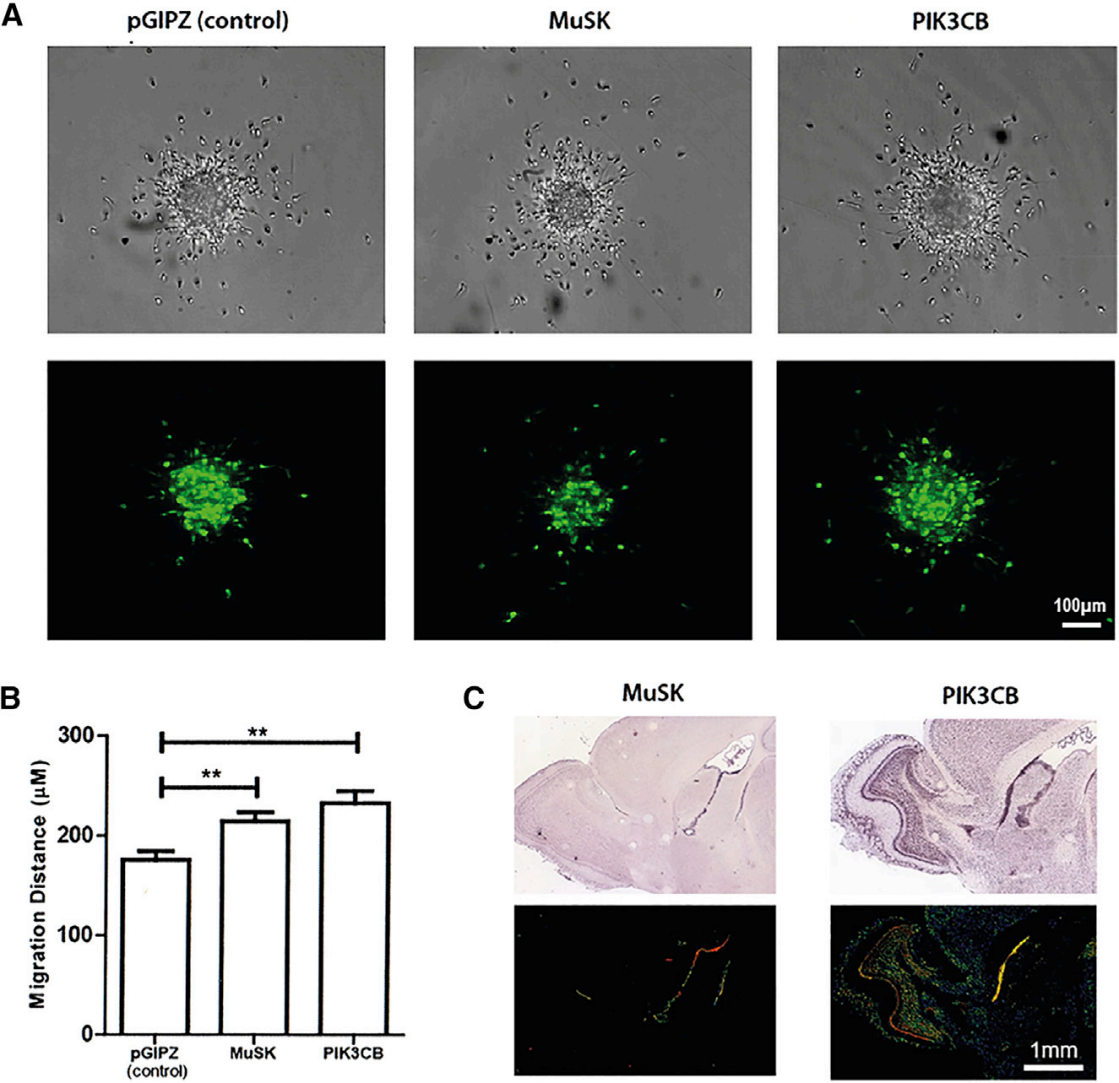
Knockdown of *MuSK* and *PIK3CB* Increased Migration of Neuroblasts from 3D Spheroids (A) Representative images of RMS spheroids nucleofected with shRNA plasmids targeting *MuSK* and *PIK3CB*. Scale bar, 100 μm (B) Quantification of the distance migrated by nucleofected neuroblasts shows that knockdown of both *MuSK* and *PIK3CB* increased the average migration distance. (C) Images showing the expression of *MuSK*, *LRP4*, and *PIK3CB* in sagittal mouse brain sections (taken from Allen Brain Atlas). Scale bar, 1 mm.

We selected two less-well-studied kinases that significantly increased neuroblast migration in the spheroid screen when inhibited. The kinase MuSK was selected based on analysis of the affinity matrices and phosphatidylinositol-4,5-bisphosphate 3-kinase catalytic subunit beta isoform (PIK3CB) was selected because it was the only indicated binding partner of GSK2336394A, a compound validated to increase migration. Knockdowns (KD) for each kinase were generated with short hairpin RNAs (shRNAs) and verified by qPCR (Figure S7E). Migration of neuroblasts expressing plasmids for *pGIPZ* (GFP controls), *MuSK* KD and *PIK3CB* KD were measured. KD of both *MuSK* and *PIK3CB* significantly increased the average distance cells migrated compared with control cells (Figures 7 and S7) Figure S7, as predicted from our results. Interrogation of the Allen Brain Atlas also shows that *PIK3CB*, and *MuSK* and its ligand *LRP4*, are specifically highly expressed in the ventricular region (Figure 7C), further supporting their role in neurogenesis.

## Discussion

The results presented here detail the establishment of an *in vitro* phenotypic assay to study the migration of SVZ neuroblasts. Whereas several *in vitro* migration assays have been developed, to our knowledge none have been coupled to drug-screening approaches. An evolution of the hanging drop assay, 3D spheroids are generated from cells dissected from the mouse RMS in a robust and standardized way. The assay has been designed to be easy to implement and cost-effective using ULA round-bottomed plates. The spheroid assay is compatible with the vital dyes: CellTracker, propidium iodide, and Hoechst, as well as with cell nucleofection of KD constructs. It could therefore be used for this physiologically relevant higher-throughput assay and also to study mechanisms regulating migration and potentially other cellular behaviors. Whereas several *in vitro* migration assays have been developed, to our knowledge none have been coupled to drug-screening approaches. These results demonstrate the utility of the 3D spheroid migration assay to assess small-molecule regulation of SVZ neuroblast migration. Twenty-one compounds significantly increased migration of neuroblasts and 36 compounds reduced their migration. Confirmation studies of two kinase pathways were performed amongst previously uncharacterized kinases that may have regulatory roles in neuroblast migration.

The biggest source of variation was from the initial RMS dissection. In addition to varying the starting cell population, imperfect dissection occasionally resulted in increased cell death, and thus smaller, less healthy spheroids with increased debris. One potential improvement could be to use transgenic mice, such as the DCX-GFP mouse where the RMS is labeled with a fluorescent protein (Nam et al., 2007). In fact, the early development of this assay was completed with one such mouse strain, the GAD65-GFP mouse (De Marchis et al., 2004; Nam et al., 2007), which made the dissection process far easier due to much more accurate visualization of the SVZ while also reducing preparation time, reducing cellular debris, and making imaging and quantification much easier. The spheroid migration assay has been developed to study neuroblasts. However, there is no reason why it cannot be applied to study different migratory cell types. For example, the SVZ also gives rise to oligodendrocyte precursor cells and visualisation of migration of these cells could be accomplished with OLIG2-GFP mice.

Automated image analysis has many advantages. It can substantially cut down analysis time and permit quantification that is technically difficult and/or laborious to do manually. It can also eliminate the inherent variability of manual scoring because computer measurements are not subject to external factors, such as human fatigue, illumination, or ambient noise. However, care must be taken at all steps of a high-content screen to avoid inconsistent imaging, artifacts, or contamination from foreign debris as this will severely compromise the image analysis results (Buchser et al., 2004). The automated analysis toolset developed in this article can quantify multiple parameters and can be optimized to quantify more if needed. It is important to consider that migration is a multistep process. The initiation, maintenance and termination of motility, speed, directionality, and percent of cells migrating can all affect overall migration. Limiting the time series to one cell cycle can to some extent control for proliferation but the results from any screen will need to be further investigated to understand more precisely what underlies the phenotype.

It is important to keep in mind the complexity of the *in vivo* environment dictating neuroblast migration compared with the reductionist approach of *in vitro* assays. *In vivo*, cell migration is modulated by a complex cellular and extracellular matrix architecture and a combination of short- as well as long-range chemoattractant and chemorepellent molecules. It is impossible to perfectly replicate, *in vitro*, the complex network of cells, structures, and signaling molecules that cells interact with *in vivo*. Indeed, although the neuroblasts radiating from the spheroids maintain the ability to migrate in a similar way to their *in vivo* counterparts, displaying chain migration and saltatory dynamics, they lack interactions with other RMS components, such as vasculature. It is widely accepted, in addition, that cells growing in a monolayer on plastic dishes have little in common with the complex 3D multicellular organization found in living organisms (Duval et al., 2017; Haycock, 2011). For this reason, it is believed that their form of migration is not equivalent to the chain migration typically observed *in vivo*. Our 3D spheroid assay is likely to more accurately reflect *in vivo* conditions then do monolayers. Explants are small (<1 mm^2^) chunks of SVZ or RMS tissue that are embedded in an extracellular matrix, such as Matrigel. Neuroblasts contained within the explant are able to migrate out radially in 3D allowing the measurement of multiple parameters, such as speed and directionality (Comte et al., 2011). Explants retain the complex cellular architecture of the brain and recapitulate both single-cell and chain migration (Comte et al., 2007). Leong et al. (2011) utilized explants to demonstrate that the ROCK1 inhibitor Y27632 disrupted chain formation. However, every explant is inherently different, leading to an unavoidably high sample-to-sample variation and they are thus not suitable for HTS. Another limitation of explants is that because the tissue remains intact, genetic manipulations are almost impossible without the use of previous *in vivo* work. Two separate studies used an embryonic organotypic brain slice culture, which identified the Src family kinase inhibitor PP2, the PKC inhibitors BIM1 and Ro318220, and 11 novel nonannotated compounds as disruptors of cortical radial migration (Jossin et al., 2003; Zhou et al., 2007). *In vitro* slice assays are even more naturalistic and have been successfully used to study RMS migration (James et al., 2011; Kim et al., 2009; Nam et al., 2007); however, they are impractical for HTS formats as only a few sagittal slices containing the RMS can be generated per animal.

The development of this 3D spheroid neuroblast migration assay is a useful tool for identifying pathways and small molecules that mediate neuroblast migration. The higher-throughput assay with the PKIS library validated the utility of the assay, and two hits, MuSK and PIK3CB, were further confirmed by KD of these genes. These data can be used in the future to develop therapeutic strategies for inducing emigration of RMS neuroblasts for injury or reducing infiltration in cancer from the RMS. Use of the PKIS library for neuroblast migration is a good example of re-purposing libraries, it was previously used to screen for compounds that could impact necroptosis (Hildebrand et al., 2014) and the sleeping sickness protozoan (Amata et al., 2016). More similar to our goals, PKIS was used in a high-content screen to find targets that could promote neurite outgrowth in primary neuronal cultures as a model for axon regrowth after injury to the brain (Al-Ali et al., 2015). Kinases that promoted or repressed neurite outgrowth were identified. A top hit for increasing neurite outgrowth, which showed polypharmacology, was tested *in vivo* in a mouse model of spinal cord injury and the compound promoted growth of corticospinal axons (Al-Ali et al., 2015). This study demonstrates the importance of developing physiologically relevant screens for neuroregeneration so that promising compounds *in vitro* can be reliably translated to *in vivo* trials (Al-Ali et al., 2015).

Kinases are key mediators of cell migration and invasion processes in brain cancer, via regulation of mesenchymal genes and rearrangement of cytoskeletal components. Cyclin-dependent kinase 5 (CDK5) modulates neuroblast migration through phosphorylation of cytoskeletal proteins. CDK5 deletion is associated with impairment in chain formation, speed, directionality, and extension of leading processes of neuroblasts (Hirota et al., 2007; Kawauchi, 2014). The cell-polarity factors glycogen synthase kinase-3β and protein kinase Cζ are needed for centrosome reorientation (Higginbotham et al., 2006). Inhibition of the PI3KAkt pathways is known to disrupt neuroblast migration and regulates neuroblast migration in stroke (Katakowski et al., 2003; Meng et al., 2010). Despite the roles of a number of protein kinases in migration being well studied, the mechanisms by which each protein kinase is involved in neuronal migration remains far from complete. Therapeutic strategies targeting kinases has been a major focus of drugs for tumorigenesis and metastasis and our assay can be incorporated into this important effort. The phenotypic assay also revealed compounds that blocked migration. These data can be used in the future to provide potential therapeutic strategies for cancer or in research into the mechanisms of neuroblast migration in the RMS. The kinase targets of the compounds that inhibited migration are yet to be further validated experimentally. Nevertheless, initial analysis of the affinity matrices provides confidence in the assay by identifying pathways known to be critical to neuroblast migration and also indicates the role of some lesser-studied kinases (Supplemental Discussion).

The large diversity in chemical structures of hit compounds suggests that neuroblast migration may be promoted through modulating a variety of target kinases and corresponding host networks. Additive effects may therefore be achieved by co-treating spheroids with hits that act via distinct biological targets. Applying machine-learning algorithms may also help to illuminate the most relevant biological targets and systems. One potential option would be to use Support Vector Machines. These are computer-based learning algorithms that recognize patterns given examples belonging to each of two categories and build a model that can classify new examples into either category. Thus, they can be used to predict kinase inhibition profiles that will have maximal biological effect.

In conclusion, we have developed an *in vitro* neuroblast migration assay coupled to a bespoke screening approach, the combination of which offers several distinct advantages. It uses primary cells rather than transformed cell lines and thus it likely better represents neuroblast *in vivo* behavior. The spheroid 3D organization was chosen as it is more naturalistic than monolayer cultures. Our proof-of-principle work shows broad applicability of the technique and also that it is relatively fast, easy, and cheap and thus accessible to most laboratories interested in mechanism discovery. Finally, automation of cell imaging and quantification renders our approach suitable for pharmacological screens of small-molecule modulators.

## Experimental Procedures

### Culturing 3D RMS Spheroids

P3-P5 CD1 wild-type mice pups were culled and their brains removed and sliced into 300-μm sections on a microtome. To dissect out the RMS tissue, the OB and the anterior-most two to three sections containing the RMS were collected in ice-cold Hanks' balanced salt solution (HBSS) (Gibco 10888-022) (Figure S1). Under a dissection microscope and using 27G syringe needles the RMS was carefully dissected and transferred into a 2-mL Eppendorf tube on ice. The RMS was identified as the translucent, region at the center of the OB sections which are cell dense and thus appear dark in Nissl stains (Figure S1). As the RMS is followed back into the more posterior sections, it elongates in a C shape. This was also collected until the ventricle begins to open up. We did not collect RMS tissue from the lateral ventricle region as it contains ciliated ependymal cells, which we noticed could prevent correct spheroid formation. The experiments were performed in accordance with the UK Animals (Scientific Procedures) 1986 Act, UK Home Office. All animal work was approved by the UK Home Office, License #30/2496, and the University of Oxford Department of Physiology, Anatomy and Genetics Departmental Ethical Review Committee.

The dissected RMS tissue was spun down briefly using a bench top centrifuge and the HBSS carefully removed. The tissue was then resuspended in 2 mL Accutase (Thermo Fisher A1110501) and carefully dissociated by gently triturating with a P200 Pipetman. It was very important that all cells were dissociated from one another. The Accutase was diluted out by adding 2 mL of the cells to 8 mL of NB-A+ medium (Neurobasal-A) (Gibco 10888-022) supplemented with 1% Glutamax (Gibco 35050-038) and 2% B27 (Gibco 17504). The cell suspension was transferred through a 40-μm cell strainer (Corning 352340) into a 50-mL Falcon tube and washed with a further 10-mL of NB-A+. The cell suspension was spun down at 300 × *g* for 5 min, resuspended in 1 mL of NB-A+ medium and counted using a hemocytometer. Typically, we collected approximately 500,000 cells per mouse. The cells were diluted to a concentration of 50,000 cells per ml of NB-A+ and 5,000 cells were added to each well of a 96-well ultra-low adhesion round bottom plate (Greiner 650970). The culture plate was then sealed with a gas-permeable seal (4TI 044850) and incubated overnight at 37°C, 5% CO_2_, during which spheroids formed. It was critical to not spin the plate down as this trapped cell debris in the center of the spheroid.

The spheroid plate was removed from the incubator and left to cool to room temperature. Matrigel was diluted to 50% in ice-cold NB-A+ medium. The spheroid plate was then briefly chilled in ice and the seal removed. Matrigel (25 μL) was then carefully trickled down the side of each well using a multichannel pipette and the plate returned to ice for the Matrigel to settle to the bottom of the well. The plate was then re-sealed and left for 3 min to warm to room temperature before replacing in the incubator for the Matrigel to set.

### Preparation of shRNA Plasmid DNA

Plasmid DNA was prepared as per the EndoFree Maxi Kit (QIAGEN, 12,362). In brief, stock cultures from the Dharmacon mouse shRNA library (cat. no. RMM5829) were cultured overnight at 37°C in 3 mL Luria-Bertani (LB) broth supplemented with ampicillin. The following day the cell suspension was plated overnight on LB agarose supplemented with ampicillin. The next day, three single colonies were picked per plate and transferred to 3 mL of LB broth supplemented with ampicillin for 4 h before being transferred to 250 mL of LB broth supplemented with ampicillin and left to shake at 37°C overnight. The following morning the cultures were processed as per the QIAGEN kit protocol apart from the following change to the final stage. Once the DNA pellet had been precipitated out using isopropanol, it was redissolved in 500 μL pure water and transferred to a 2-mL Eppendorf tube. Ethanol (1,400 μL, 100%) was added, mixed well, and spun down at maximum speed for 5 min on a desk top microcentrifuge. The ethanol was carefully removed and the DNA pellet allowed to dry completely. The DNA pellet was resuspended in 50 μL pure water and the concentration measured using a NanoDrop. Table S6 shows the shRNA constructs used for kinase KDs. Table S7 shows the primers used to verify the KDs.

### Reverse Transcription

Complimentary DNA (cDNA) was synthesized using SuperScript III Reverse Transcriptase. One microliter of 250 ng/μL random hexamers (N8080127) and 1 μL of 10 mM dNTP mix (Thermo Fisher R0191) was added to 1 μg of DNase-treated RNA, and made up to 13 μL with nuclease-free H20 (Sigma-Aldrich, Gillingham, UK). The mix was incubated at 65°C for 5 min to denature RNA secondary structure and then incubated on ice for 1 min. The mix was made up to 20 μL with 4 μL 5× first strand buffer (Life Technologies), 5 mM dithiothreitol (18080-044), 40 units RNaseOUT (10777), 1 μL (200 units) SuperScript III Reverse Transcriptase (Invitrogen, 18080-044) and 3 μL of nuclease-free H_2_O. Samples were incubated in a PCR machine on an RT protocol: 25°C for 5 min, 55°C for 1 h (optimal temperature for Superscript III), and 70°C for 15 min (termination of the reaction). Samples were diluted in 100 μL nuclease-free H_2_O for qPCR.

### Primer Design

PCR primer pairs were designed to amplify an amplicon of approximately 100 base pairs. DNA sequences for genes of interest were obtained from the University of California, Santa Cruz, genome browser (http://genome.ucsc.edu) and primers designed using Integrated DNA Technology (IDT) software (http://www.idtdna.com/primerquest/home/index). GC content was kept between 40% and 60%, and primers were designed with an annealing temperature between 55°C and 65°C with only 1°C between the pair. They were designed to have minimal self-complementarity and no complementarity to the other primer and were tested for single-peak melt curves using uMelt software (https://www.dna.utah.edu/umelt/umelt.html). RT-PCR primers were designed to span large exon boundaries to prevent the amplification of any residual genomic DNA. The GAPDH primer pair used to test for genomic DNA contamination did not span exon boundaries. Oligonucleotides were synthesized by IDT. They were resuspended in nuclease-free H_2_O to a stock concentration of 100 μM and used at a working concentration of 2 μM.

### RT-PCR

The reaction consisted of 33 ng template nucleic acid (4 μL), 10 μL 2× SYBR Green PCR Master Mix, 0.2 μM primer mix (3 μL), and nuclease-free H_2_O to make a total reaction volume of 20 μL. All PCR reactions were carried out with one negative control for each primer pair, where H_2_O was used as the template. RT-PCR reactions were carried out on a Chromo4 Real-Time PCR detector (Bio-Rad, Bath, UK) using the following parameters: initial denaturation 95°C for 15 min, denaturation 95°C for 30 s, annealing 60°C for 30 s, extension 72°C for 30 s × 45 cycles. Fluorescence was recorded at the end of each cycle. A melt curve was carried out from 60°C to 95°C with a 10-s hold and a plate read at every 1°C increment. Each reaction was carried out with a technical replicate and with three biological replicates. Relative transcript quantification used the cycle threshold (CT) values for the ΔΔCT method to determine relative expression of the target gene to confirm KD. The expression of the gene of interest was calculated by normalizing against the housekeeper gene, and two-tailed, independent t tests were performed in Prism 6 (GraphPad) to test for level of KD of the gene, with significance determined as ^∗^p < 0.05, ^∗∗^p < 0.01, ^∗∗∗^p < 0.001, ^∗∗∗∗^p < 0.0001.

### Postnatal Electroporation

*In vivo* SVZ electroporation was carried as in previous work (Al-Dalahmah et al., 2020; Sun et al., 2018). In brief, plasmids used for postnatal electroporation were prepared with EndoFree Plasmid Maxi Kits as above. Before use, 1% Fast Green in PBS (0.22 μm filtered) was mixed with the plasmid solution at 1:10 for visualizing injection. P0–P1 pups were deeply anesthetized by hypothermia on wet ice. Plasmid solution (∼2 μL, 5 μg/μL) was injected into the right lateral ventricle via a pulled glass capillary. Very brief, electrical current was passed across the snouts (−100 V, 50 ms ON with 850-ms intervals for 5 cycles) (BTX ECM 830) by tweezer electrodes coated with conductive gel. After surgery, pups were kept in a 37°C warming box for recovery and then returned to their cage. The pups were monitored for the following 4 days to check for adverse reactions to the procedure and on the 5th day the pups were culled and the electroporated hemispheres removed for spheroid preparation.

### Nucleofection of Primary RMS Cells

Cells from the RMS were nucleofected via a modified version of Lonza protocol VPG-1001. In brief, the RMS was dissected out as described in the main text, dissociated, counted, and split across 2 to 4 different 15-mL Falcon tubes containing 1× 10^6^ cells and 5 mL NB-A+ medium. The cells were spun down at 300 × g for 5 min, the supernatant removed and then very carefully resuspended in 50% of the standard nucleofection solution diluted in HBSS minus calcium and magnesium (41 μL of Nucleofector Solution + 9 μL of supplement + 50 μL of HBSS) and 5 μg DNA before being transferred into the certified cuvette. Nucleofector program G-13 was used instead of the recommended O-005. Pre-warmed culture medium (500 μL) was added into the cuvette and the sample was then transferred by the supplied pipette into 1 mL medium and centrifuged at 300 × *g* for 5 min. Cells were then suspended with 4 mL fresh medium, plated in a single well per condition in a 6-well plate and left to recover at 37°C. After 4 h the cells were collected and used as normal for the remainder of the spheroid assay.

### PKIS High-Content Imaging

We used the IN Cell Analyzer 6000 high-content imager for image acquisition. This system comprises both bright field and a line-scanning laser confocal microscope making it ideal for quick 3D cell imaging, albeit at a slightly poorer resolution than standalone point-scanning confocal microscopes. A protocol was established on the IN Cell acquisition software that imaged the center of each well of the Greiner ULA plate. The starting plane was set by the microscope's in-built “software autofind” function and nine Z slices were imaged at 25 μm per well, which amounted to a total section of 200 μm. Before starting the imaging experiment a trial scan was completed to check the position of the spheroid in each image. Should any spheroid be dramatically off center or shifted in the Z plane the acquisition field was manually set for that well. All images were taken with the 10× 0.45NA objective. The IN Cell has four lasers (UV, blue, green, and red) and four capture filters (DAPI, GFP, dsRed, and Cy5). Images were taken above the well using an orange bright-field diode and dsRed emission filter at an exposure of 0.2 s. When fluorescence was used the exposure was set based on the individual signal level of that experiment. To scan each plate, it took around 15 min on bright field only and around 25 min when using fluorescence.

## Author Contributions

M.D., D.E., and F.S. carried out experimental design. M.D. and V.M. carried out the experiments. M.D., V.M., and F.S. carried out data analysis. M.D., V.M., D.E., and F.S. wrote the paper.

## Supplemental Information

Document S1. Supplemental Experimental Procedures, Figures S1–S7, and Tables S1–S7

Document S2. Article plus Supplemental Information
