# Supplementary material for: A Semi-automated and Scalable 3D Spheroid Assay to Study Neuroblast Migration
Source: Stem Cell Reports. 2020 Aug 6;15(3):789–802. doi: 10.1016/j.stemcr.2020.07.012 (PMC7486343; doi:10.1016/j.stemcr.2020.07.012)
Supplement: Document S1. Supplemental Experimental Procedures, Figures S1–S7, and Tables S1–S7 [file mmc1.pdf]

Stem Cell Reports, Volume *15*

Supplemental Information

A Semi-automated and Scalable 3D Spheroid Assay to Study Neuroblast Migration

Martin Ducker, Valerie Millar, Daniel Ebner, and Francis G. Szele

Supplemental Methods

The following shRNA plasmids were selected from the Dharmacon GIPZ Lentiviral Mouse shRNA Library (RMM5829).

Table S6. shRNA constructs used for kinase gene knockdown

Reference	Gene Target	Vector	Sense Sequence
Abl1#1	*Abl1*	pGIPZ	CTCTTATAAATGACATGTA
Abl1#2	*Abl1*	pGIPZ	GAACCACCATTCTACATAA
Hunk#1	*Hunk*	pGIPZ	CAAAGAGATCTTTCTTTAT
Mertk#1	*Mertk*	pGIPZ	CTGTTATATTCCCGATTAA
Mertk#2	*Mertk*	pGIPZ	CGGAACATAAAAATGTCAA
MuSK#1	*MuSK*	pGIPZ	GGAGCTTAAGGATCCATAA
Nek9#2	*Nek9*	pGIPZ	AGATGTTTACCCAACTACA
Pik3cb#1	*Pik3cb*	pGIPZ	CTCACATCGGTCAAAGATA
Pik3cb#2	*Pik3cb*	pGIPZ	CTGGAGAACTTGGAAGATA

Table S7 Table of primers used for confirmation of shRNA knockdown

Gene	Forward Sequence	Reverse Sequence	Source
Plk1	CTTCGCCAAATGCTTCGAGAT	TAGGCTGCGGTGAATTGAGAT	Life Technologies
Riok2	TAAGCTGTTCAACAATCCCTCC	GCTGCTTGGTAAACACATTGG	Life Technologies
Abl1	CAGCAGCCTGGAAAAGTTCTT	CCCTGCCCCTTTGATAAAATGC	Life Technologies
Hunk	GTGCTGACGGGAGAAAAGGTA	GGGTGTCGGATCATCTGCTG	Life Technologies
Mertk	CCTAACCGTACCTGGTCTGAC	GGGAGGGGATTACTTTGATGTTG	Life Technologies
MuSK	TACAGAGGGGAGGTGTGTGAT	TCCCGGTAGGAGGTGTTGAA	Life Technologies
Nek9	TACGAGCGACACTGCGATTC	ACGCGGATGGGGATGTAGT	Life Technologies
Pik3cb	CTATGGCAGACAACCTTGACAT	CTTCCCGAGGTACTTCCAACT	Life Technologies

DNAse treatment of RNA To remove residual genomic DNA contamination, all RNA samples were treated with TURBO DNA- freeTM (Invitrogen AM1907). 2µg of extracted RNA was combined with 1µl of TURBO DNase, 2.5µl of TURBO DNase Buffer and made up

to 25µl with nuclease-free $H_2$0. The sample was incubated for 50 min at 37°C. The DNase was subsequently inactivated by incubation with 6µl of DNase Inactivation Reagent at RT for 5 min, vortexing every 30 seconds. The sample was then centrifuged at 13,000 RPM for 90 seconds and the supernatant was transferred to a new Eppendorf tube and stored at -80°C.

Supplemental Results

The RMS was dissected (Figure S1) and spheroids generated as described in the Supplemental Results. The assay was initially trialled with GAD65-GFP mice (Figure S2A). Neuroblasts migrating out from the spheroids are able to self-organise into chains closely recapitulating *in vivo* migration (Figure S2B), a critical feature in a physiologically relevant migration assay. Spheroids ranging from 600 to 10,000 cells were generated and all resulted in robust migration (Figure S3A). In subsequent work we systematically eliminated factors confounding image analysis (Figure S3B).

The use of GAD65-GFP or similar transgenic mice is recommended for anyone new to RMS dissections to improve dissection accuracy, consistency and speed. The RMS is more translucent and distinguishable from surrounding tissue and most researchers are able to make this distinction with short training times. We estimate that 75-85% of cells in our samples are neuroblasts. Because we generate a stock of single cells, mix them and aliquoted into each well there is little variation in the extent of migration from control neurospheres.

GAD65-GFP cells form spheroids

To confirm that the Greiner ULA plate system could be applied to RMS cells, we set up a pilot experiment in which the RMS was dissected from GAD65-GFP mice (Figure S2A). This is a convenient mouse strain for this work because the majority of neuroblasts in the RMS express GFP making dissecting and subsequent imaging relatively straightforward. As well, we have extensively studied GAD65-GFP neuroblast migration in slices with 2-photon microscopy (Nam et al., 2007). Two P4 mice pups were used, which provided sufficient cells for culturing 5,000 cells in each of the middle 20 wells of a 96 well plate. The cells naturally reaggregated overnight and formed well-defined and uniform spheroids. In the pilot experiment one well had to be excluded due to debris, nevertheless 19 uniform spheroids with an average 2D surface area of 15,603±214 μm^2 (Figure S2A). Thus, spheroid are expected to be normally distributed with variances in initial sizes expected to arise only due to statistical fluctuation.

Neuroblast migration from 3D RMS spheroids recapitulates *in vivo* migration and is distinct from neurosphere migration

A critical feature in developing an SVZ neuroblast migration assay is that it recapitulates in vivo features such as chain migration that occurs *in vivo*. It is argued that chain migration is orchestrated by SVZ glial tubes, however, as Figure S2B illustrates, neuroblasts radiating out from the spheroids were able to self-organise into chains. As evidenced by the GAD65-GFP expression in Figure S2A, RMS spheroids were predominantly composed of

neuroblasts. It was observed that neuroblasts routinely changed directions while migrating and alternated between pauses and bursts of fast migration, which is consistent with previously described behaviors of neuroblasts migrating in slices (James et al., 2011). Almost all cells emerged from the spheroid in chains and then continued to migrate as individual cells, often following the path of previous neuroblasts. As a means of rapid quantification of images, a relatively simple segmentation of the radiation area was quantified using the InCell analysis Software (Methods) (Figure S5). Thresholding enabled the segmentation of both the central spheroid and the halo of cells that has migrated out of the spheroid in chains. The number of single cells could easily be counted for either the entire image or within a given area. To quantify distance of migration it is possible to expand the area of the spheroid by set distances and quantify the number of cells that occupy that area. Repeating this several times creates defined zones that can provide data on the frequency distribution of the distance migrated by the neuroblasts. The spacing of these bins can be set depending on the requirements of the experiment. However, it is worth considering how to plan to compare across the sample set. The ratio of the area of the halo to the number or area occupied by single cells may also be used as an indicator of conditions that modulate collective or chain migration. Although single neuroblasts could be discerned at the end of the experiment, it was not possible to capture time-lapse single cell migration using this method, however, single cell tracking may be an option for future time-lapse experiments.

It is important to emphasize here that the 3D RMS spheroids are distinct from SVZ neurospheres used in the field for ascertaining SVZ stem cell properties, such as self-renewal, proliferation and fate choice. Neurospheres have a heterogeneous cell composition that varies depending on age, seeding density, culture conditions, and passage number. Nevertheless, the predominant cell type is generally considered to be the rapidly dividing transit amplifying cells (Gil-Perotin et al., 2013). Dcx+ neuroblasts are only found in very few numbers during the self-renewal phase of neurosphere cultures. This concurs with the consistent finding from our group that the radial migration from neurospheres is almost entirely of glial cells. Moreover, glial migration is amoebic in nature and does not display the mixture of chain and single cell migration evident in both the explant assay and now the spheroid assay.

Assay Optimisation

Once it had been determined that RMS spheroid formation was robust it was important to optimise a few criteria. The first of these was spheroid size or cell number per well. The greater the cell number the more continuous the radiating cell halo becomes, making image quantification more consistent. Moreover, a greater number of cells permits greater complexity in 3D cellular architecture. However, the transfer of nutrients into the spheroid centre gradually decreases with spheroid size, potentially causing variations in cell viability or metabolism. In SVZ neurospheres this is known to lead to the formation of hollow necrotic cores once the diameter exceeds ~150µm (Ge et al., 2012). In addition, monitoring cell migration with videomicroscopy is limited by the field size and camera aperture, thus smaller spheroids may enable migration to be tracked over longer periods of time. A balance is therefore required to retain physiological relevance, while permitting high content screening.

Figure S3A shows spheroids plated at cell densities between 600 and 40,000 cells per well. Imaging at 24 hours shows that the spheroids with over 10,000 cells at the start of the assay contain a lot of migrating cells, but the cells with highest levels of migration do not remain within the field of image, and therefore cannot be reliably measured. The largest spheroids also develop necrotic centres due to the lack of nutrients reaching the spheroid core. However, the spheroids tested with less than 2,500 cells do not have sufficiently large numbers of cells to migrate from the spheroid, and therefore do not make a reliable high throughput assay. The best compromise under the experimental conditions and equipment available were spheroids of 5,000 cells.

Matrigel is a soluble basement membrane biomaterial extracted from Engelbreth-Holm-Swarm mouse tumours; it contains several molecules including laminin, collagen IV, heparan sulfate proteoglycans, and entactin/nidogen-1, 2. It is liquid below 4°C but sets to a gel above 10°C. We used Growth Factor Reduced Matrigel (BD 356231). Importantly, Matrigel is viscous; it has a protein concentration of 8-12mg/ml and will sink in water. Matrigel™ is relatively expensive, therefore we next sought to determine whether the volume of Matrigel could be reduced. The concentration of Matrigel used is known to strongly influence neuroblast migration so we were reluctant to vary this dramatically (Ward and Rao, 2005). However, experience from explant assays indicates that neuroblasts grow well *in vitro* in drop volumes down to ~10µl. When pipetted carefully with a single tip pipette we confirmed that the volume of Matrigel used could be reduced to 20µl. However, when attempted with a multichannel pipette this volume proved too restrictive as roughly 10% of the spheres failed to embed correctly. The smaller volume of Matrigel displaced the spheroid from the centre of the well, instead of enveloping it, resulting in the spheroids being displaced to the side (Figure S3B). While this may not necessarily effect invasion it hindered both imaging and analysis as steep sides of the U bottom create image aberrations (Figure S3B). We hypothesised that this was due to the leading edge of the Matrigel beginning to polymerise as it reaches the bottom of the well. To overcome this, the culture plate with the spheroids was cooled on ice prior to embedding and the Matrigel volume was increased to 25µl. This resolved the problem of spheroid centring but introduced a second complication. Using ice resulted in watermarks on the bottom of the plate that introduced large artefacts into the brightfield image background (Figure S3B). These proved very difficult to prevent via simple cleaning measures. Wiping with alcohol wipes was either too mild to remove the mark or too harsh; either creating scratches on the plate surface or dislodging the Matrigel plug. To cool the spheroid plate, we therefore used a large reusable icepack recycled from a consumable delivery covered in a single sheet of tissue paper.

Another strategy considered was miniaturisation to a 384 well format. The smaller volumes and tighter gradient of curvature of the U-bottomed 384 well plates (Corning) allowed the volume of Matrigel per well to be reduced to just 10µl. However, because of the increased well curvature, large spherical aberrations were created making them unsuitable for migration assays that require a large image field (Figure S3B). This, in addition to the increased plate cost and technical difficulty, confirmed the 96 well Greiner ULA plate with 25µl 50% Matrigel as the best format.

The other most pertinent issues to overcome in the experimental set up are represented in Figure S3B. The media composition needed to be optimised to sustain dense spheroid formations while minimising potential variants. Spheroids formed in standard neurobasal

media supplemented with B27 “complete media”. There was no need for additional supplementation with serum or growth factors. However, culture medium with low insulin (5nM) did not support spheroid formation. Debris in the cultures was important to minimise as this disrupted spheroid formation and integrity. Debris came in two forms 1) contamination (large debris), and 2) Cellular debris. This assay will test the experimenter’s aseptic technique. Any contamination that falls in the well will be focused into the centre and incorporated in the spheroid. Arm sleeves are highly recommended to prevent fibre contamination from lab coats. Cellular debris were cells that had not reached the centre of the well and were not incorporated in the spheroid. They were not necessarily dead and could retain the ability to migrate. This did not create a huge obstacle to manual image analysis where the experimenter can be more discerning but for automation it could be a problem. The amount of debris is largely dependent on the accuracy and speed of the dissection. Very little debris is experienced when using a fluorescently labelled mouse line, such as the GAD65-GFP or DCX-GFP strains. However, when using wild type tissue, a degree of contamination was unavoidable (Figure S3B). In some instances where the SVZ was accidently dissected, ciliated ependymal cells could also disrupt spheroid formation entirely. The best strategy to overcome this is to improve dissection technique. If dissecting more than four brains in one sitting it is also advisable to add in a recovery period after the dissection to let the cells recover and limit the addition of dead or dying cells to the ULA plate. Even with these measures it was impossible to form a spheroid with absolutely no debris. For very high-quality images, it is advised to pick the spheroids out of the well and wash prior to embedding.

Live Dead Assay

The combination of Hoechst 33342 (ThermoFisher 62249) and Propidium iodide (PI) (Invitrogen P3566) was used to assess the viability/cytotoxicity in the spheroid assay. A concentrated stock of Live/Dead reagent was prepared by adding 5µg/ml Propidium Iodide solution and 10µg/ml Hoechst to NB-A+. Two hours prior to the final timepoint of the assay the plates were removed from the incubator, the seal carefully removed and 10µl of Live/Dead reagent added to each well. The plate was returned to the incubator for the remaining time period and assayed as normal. Both dyes worked well in RMS spheroids, however the compact spheroid mass created dye gradients necessitating staining time to be increased from 10 minutes to 60 minutes to reach equilibrium. The response of the spheroid bodies and the migrating cells to screened compounds can be used to distinguish between compounds that inhibit migration vs those that are cytotoxic (Figure 4). However, due to the cytotoxicity of the vital dyes themselves this assay should only be used as an endpoint.

CellTracker Green

CellTracker Green is a more stable form of the thiol-reactive Fluorescent Probe Calcein-AM that is suitable for long-term cell labelling (Kim et al., 2009). It passes freely through cell membranes, but once inside the cell, is transformed into fluorescent cell-impermeant reaction product. To avoid uneven dye transfer into the compact spheroid mass, the dye was added to the dissected cells prior to spheroid formation. CellTracker stained all cells in the spheroid with no obvious detriment to spheroid formation or neuroblast migration (Figure S4). Single cells could easily be identified, thus providing the option to use high content analysis software to count and measure the dynamics of single neuroblasts.

Published Kinase Inhibitor Set (PKIS) migration assay

The PKIS1 is a collection of 367 compounds that are split across 5x96 well plates and the PKIS2 is a collection of 645 compounds that are split across 7x96 well plates. We used 4,000 cells per well rather than 5,000 to increase the number of compounds that could be included. We split the experiment into 4 separate rounds where each round screened 3 plates in duplicate. Six 96 well plates of 3D RMS spheroids were prepared overnight as described above. Each spheroid contained 4,000 cells and was suspended in 80µl of Neurobasal. The compound libraries were diluted to a 5X (50µM) working concentration in Neurobasal. Columns 1 and 2 were used as DMSO controls. 20µl of working concentration compound was added to each well of the spheroid plates and they were returned to the incubator for 2 hrs to incubate. The drugs were added manually with a StarLab ErgoOne multichannel pipettor. To embed the spheroids in Matrigel the culture plates were cooled on an icepack for ~5 minutes and then 25µl of ice cold 50% Matrigel was carefully added down the side of the well. The plates were then sealed with a gas permeable, optically clear seal (4tittude) and returned to the incubator to set for 2hrs. Plates were then imaged at 2 hours (T=0) and 24 hours with brightfield microscopy. 2hrs prior to the 48hr endpoint the Live/Dead assay was added to each well and the plates were imaged on brightfield, UV and 488. Figures S8 and S9 show compounds, migration images and concentration response curves for kinase inhibitors that increased and decreased migration, respectively.

Analysis of the binding targets of the small molecules indicates potential genes that regulate neuroblast migration.

The PKIS is comprised of inhibitors that function via competitive binding with the cofactor ATP. Because the ATP binding pocket is conserved but not identical across the kinome, many of the PKIS compounds have some degree of promiscuity in their kinase activity. Consequently, the kinase for which a compound was originally prepared might not be the only kinase inhibited, and importantly the original kinase may not be a contributor to the phenotype resulting from compound treatment. Both the PKIS1 and PKIS2 have been characterised to try to provide insights into their likely molecular targets. The PKIS1 was analysed using the Nanosyn enzyme assay panel to determine the binding affinity to 224 recombinant human kinases (Elkins et al., 2016) and the PKIS was analysed using the KINOMEscan competition binding assay against 468 kinases. The results for both provide a percentage of kinase bound the inhibitor compared to DMSO (Drewry et al., 2017). It is important to note that neither panel is exhaustive so will not identify all potential interactions and that there are many kinases characterised for PKIS2 that are absent from the PKIS1 data set. It is also important to note that affinity is not necessarily correlated with potency.

We sought to select the most likely candidate kinase targets from the compounds screened for confirmation studies. The primary method used was to select candidate hit kinases from the affinity matrix for compounds that were validated to increase and decrease migration. To account for the promiscuous polypharmacology only hits in which there were 2 or fewer kinases with an affinity greater than 90 were analysed. The top two kinase hits were then selected for each compound as potential regulators of migration. The second approach we used was to analyse the distribution of binding affinities across the entire data set to identify kinase targets that are concentrated in the higher and lower migration groups. The rationale for this is that the results from the Nanosyn and KINOMEscan profiles do not necessarily

perfectly correlate with inhibition efficiency, so simply screening for hits greater than 90 will indiscriminately mask targets that have low affinity. Conversely, there may be kinases that have a consistently high affinity that are picked up in the screen, but do not actually have any functional activity. The cut offs used were if the migration area was >1.5x control, compounds were allocated to the high migrating pool and if it was <0.5x control, the compounds were allocated to the low migrating pool. All toxic compounds were removed from the analysis. The average affinity of each kinase to all compounds in the two categories were then calculated, compared and ranked. The kinases with a Δ high:low above 5 were considered as potential hits, where inhibition may increase or decrease migration for both PKIS1 and PKIS2 (Table S1). The kinases with a Δ high:low less than -5 were also considered as potential hits where inhibition decreased migration for PKIS1 (Table S1). For PKIS2 the number of putative hits was >200 using -5 as the threshold, and so it was adjusted to -13 (Table S1). The lists of putative hits selected from each analysis method were then compared.

STRING is an accumulated database of known and predicted protein-protein interactions, including both direct (physical) and indirect (functional) associations (Szklarczyk et al., 2017). It was a useful tool to utilise for this screen, to interrogate the kinases that are likely central to the molecular mechanisms behind migration. Proteins that were hits in the screen and have known associations with other proteins that were also hits could be important in future experiments that investigate the migration process. Figure S6A shows the STRING connections between the kinases that, when inhibited, increased migration. These kinases therefore may well normally inhibit migration in the brain. The stem cell growth factor receptor Kit may be an interesting kinase to investigate further as it links two different protein pathway hubs. PLK1, PLK2 and PLK3 were all hits for increasing migration and have known interactions with other proteins that produced the same phenotype and could have important roles in regulating migration. EGFR, the most common target of the compounds that increased migration, has many interactions with other proteins that significantly altered migration, including Kit.

Figure S6B shows kinases that decreased migration and the known interactions between these kinases from the accumulated STRING databases (Snel et al., 2000). MAPK14, an alias for P38A, which was the most common target of compounds that decreased migration is central to the STRING protein interaction map. It is closely associated with MAPK11, an alias for P38B, which was also a significant target for decreasing migration, and both proteins have many interactions in the network. The number of kinases that decreased migration that are closely associated with one another suggests that there may be a common mechanism in this process.

Optimising nucleofection for the spheroid migration assay

The ability to genetically manipulate cells of interest is useful in modern screening, and to demonstrate the wider application of the 3D RMS spheroid assay, we included a method to successfully nucleofect RMS cells prior to formation of 3D spheroids. Initially the Nucleofection programme O-005 was used as per the Lonza protocol (VPG-1001). However, this protocol did not yield compact, round spheroids (Figure S7A). Instead many small spheroids formed, which all collected at the bottom of the well. (A gelatinous substance in the well appeared to prevent complete unification.) We next used setting A-33, another

programme designed for nucleofection of mouse primary neural cells. After nucleofection, cells were healthy and when washed, dissociated and re-plated could re-aggregate and form spheroids. However, when these secondary spheroids were embedded the subsequent migration was limited (Figure S7B).

Another technique used to study neuroblast migration is *in vivo* electroporation. In this technique high concentration plasmid DNA is injected directly into the lateral ventricle of P0-P2 pups and then a pulse electric current is applied across the skull. This robustly labels SVZ stem cells with the plasmid and also therefore their progeny, the neuroblasts (Boutin et al., 2008). We therefore attempted to electroporate *in vivo* the pGIPZ plasmid 5 days prior to dissection. Using this method, spheroids formed correctly but the number of GFP+ cells contained within each spheroid was too low in these experiments to quantify (Figure S7C). Nevertheless most of our other in vivo electroporation experiments have resulted in many hundreds to thousands of labelled neuroblasts in the RMS and OB (Sun et al., 2018), and thus it is likely that this approach could be further optimised for Spheroid migration assays.

Therefore, two modifications were made to the protocol from the Lalli group (Falenta et al., 2013). Firstly, the Nucleofection Solution was diluted in Hanks Balanced Saline Solution minus calcium and magnesium. The rationale was that the lipophilic nature of the reagent may be disrupting membrane integrity. Secondly the nucleofection programme was changed to G-13, which has a reduced electric pulse power. With these modifications the nucleofection of dissociated neuroblasts did not disrupt spheroid formation and the efficiency was sufficiently high (~50%) to quantify migration (Figure S7D). This was confirmed with knockdown of *MuSK* and *Pik3cb* (Figure S7E-H).

Supplemental Discussion

Inhibition of phosphatidylinositol-4,5-bisphosphate 3-kinase catalytic subunit beta isoform (PIK3CB) was found to significantly increase neuroblast migration. PIK3CB is one of the four catalytic subunits of PI3K. Interestingly although in non-neuronal cells these catalytic subunits may have partially redundant functions, there is increasing evidence that in neurons their roles are more specialised, and confined to distinct receptor-dependent pathways. For instance, while the key mediator of IGF1R signalling is PIK3CA, PIK3CB is the major catalytic isoform downstream of the ERBB4 (Law et al., 2012). Activation of PIK3CB by ERBB4 is orchestrated via Yes-associated protein (YAP), the nuclear effector of Hippo signalling (Yan et al., 2017). It is therefore tempting to consider the opposing function of IGF1R and the ERBBs in neuroblast migration may involve differential activation of PIK3CA and PIK3CB. PIK3CB is also the predominant subunit associated with GPCRs (Guillermet-Guibert et al., 2008). The phosphatase and tensin homologue (PTEN), a negative regulator of PI3K activity, was shown to preferentially bind to PIK3CB compared to other PI3K catalytic subunits. PTEN deletion has been shown to disrupt neuroblast migration and, like PIK3CB, has been linked to autism (Gregorian et al., 2009; Zhu et al., 2012). However, the specific regulation of PIK3CB by PTEN in the SVZ remains unstudied.

Genetic validation with shRNA knockdown further supported a function of Muscle-specific tyrosine kinase receptor (MuSK) in neuroblast migration. MuSK is a receptor tyrosine kinase

that is mainly expressed in the muscle and plays an essential role in neuromuscular junction (NMJ) formation (Garcia-Osta et al., 2006). This function requires the interaction of MuSK with the heparan-sulfate proteoglycan Agrin and Low-density lipoprotein receptor-related protein 4 (LRP4). The role of MuSK in the central nervous system is poorly understood, however it has been reported to have a role in memory consolidation (Garcia-Osta et al., 2006; Sun et al., 2016). According to the Allen Brain Atlas MuSK, LRP4 and Agrin expression is evident in the SVZ and hippocampus and MuSK is concentrated at excitatory synapses, marked by coexpression of PSD-95 (Garcia-Osta et al., 2006; Ksiazek et al., 2007). Mice and zebrafish deficient in MuSK have defects in neural crest migration (Banerjee et al., 2011). In the absence of MuSK, streaming neural crest cells, which are normally confined to the central region of each somite, are dispersed and present throughout the somite. Specific knockdown of Agrin in the SVZ by postnatal electroporation disrupted integration, morphological differentiation and survival of new olfactory bulb interneurons (Burk et al., 2012).

The mitogen-activated protein (MAP) kinase family are well-documented regulators of migration in various cell types (Huang et al., 2004). Several are indicated by the screen as potential mediators of neuroblast migration, including MAPK8/Jnk1, MAPK9/Jnk2, MAPK14/p38a and MAPK11/p38b. Of these p38a can be found within the top hits selected by all methods used to interrogate the screen data set. The role of p38α in the SVZ remains incompletely understood but evidence supports a role in migration. It is highly expressed in the brain from E10 and is retained in the SVZ and choroid plexus of the adult brain (Sato et al., 2008). Pharmacological inhibition has been shown to decrease migration and a cell permeable p38 enhances migration *in vitro* without affecting cell survival or differentiation (Hamanoue et al., 2016). p38 also correlates with poor prognosis in several types of cancers, including glioblastoma (Goldsmith et al., 2018). p38 inhibitors have, therefore, attracted significant attention for use in chemotherapy, with at least 22 different compounds being investigated in Phase I/II. Yet despite their potential, high toxicity and off-target effects have severely limited their therapeutic value and none have reached Phase III. It is therefore of significant interest to identify the downstream effectors of p38. One possibility proposed by the screen data set in MAPK-activated protein kinase 3 (MAPKAPK3). This is a known substrate of p38 that is essential for migration of smooth muscle and endothelial cells (Hedges et al., 1999; Rousseau et al., 1997).

SI References

Banerjee, S., Gordon, L., Donn, T.M., Berti, C., Moens, C.B., Burden, S.J., and Granato, M. (2011). A novel role for MuSK and non-canonical Wnt signaling during segmental neural crest cell migration. Development *138*, 3287-3296.

Boutin, C., Diestel, S., Desoeuvre, A., Tiveron, M.C., and Cremer, H. (2008). Efficient in vivo electroporation of the postnatal rodent forebrain. PLoS ONE *3*, e1883.

Burk, K., Desoeuvre, A., Boutin, C., Smith, M.A., Kroger, S., Bosio, A., Tiveron, M.C., and Cremer, H. (2012). Agrin-signaling is necessary for the integration of newly generated neurons in the adult olfactory bulb. J Neurosci *32*, 3759-3764.

Drewry, D.H., Wells, C.I., Andrews, D.M., Angell, R., Al-Ali, H., Axtman, A.D., Capuzzi, S.J., Elkins, J.M., Ettmayer, P., Frederiksen, M.*, et al.* (2017). Progress towards a public chemogenomic set for protein kinases and a call for contributions. PLoS One *12*, e0181585.

Elkins, J.M., Fedele, V., Szklarz, M., Abdul Azeez, K.R., Salah, E., Mikolajczyk, J., Romanov, S., Sepetov, N., Huang, X.P., Roth, B.L.*, et al.* (2016). Comprehensive characterization of the Published Kinase Inhibitor Set. Nat Biotechnol *34*, 95-103.

Falenta, K., Gajendra, S., Sonego, M., Doherty, P., and Lalli, G. (2013). Nucleofection of Rodent Neuroblasts to Study Neuroblast Migration In vitro. J Vis Exp.

Garcia-Osta, A., Tsokas, P., Pollonini, G., Landau, E.M., Blitzer, R., and Alberini, C.M. (2006). MuSK expressed in the brain mediates cholinergic responses, synaptic plasticity, and memory formation. J Neurosci *26*, 7919-7932.

Ge, C., Yang, Q., Zhao, G., Yu, H., Kirkwood, K.L., and Franceschi, R.T. (2012). Interactions between extracellular signal-regulated kinase 1/2 and p38 MAP kinase pathways in the control of RUNX2 phosphorylation and transcriptional activity. J Bone Miner Res *27*, 538-551.

Gil-Perotin, S., Duran-Moreno, M., Cebrian-Silla, A., Ramirez, M., Garcia-Belda, P., and Garcia-Verdugo, J.M. (2013). Adult neural stem cells from the subventricular zone: a review of the neurosphere assay. Anat Rec (Hoboken) *296*, 1435-1452.

Goldsmith, C.S., Kim, S.M., Karunarathna, N., Neuendorff, N., Toussaint, L.G., Earnest, D.J., and Bell-Pedersen, D. (2018). Inhibition of p38 MAPK activity leads to cell type-specific effects on the molecular circadian clock and time-dependent reduction of glioma cell invasiveness. BMC Cancer *18*, 43.

Gregorian, C., Nakashima, J., Le Belle, J., Ohab, J., Kim, R., Liu, A., Smith, K.B., Groszer, M., Garcia, A.D., Sofroniew, M.V.*, et al.* (2009). Pten deletion in adult neural stem/progenitor cells enhances constitutive neurogenesis. J Neurosci *29*, 1874-1886.

Guillermet-Guibert, J., Bjorklof, K., Salpekar, A., Gonella, C., Ramadani, F., Bilancio, A., Meek, S., Smith, A.J., Okkenhaug, K., and Vanhaesebroeck, B. (2008). The p110beta isoform of phosphoinositide 3-kinase signals downstream of G protein-coupled receptors and is functionally redundant with p110gamma. Proc Natl Acad Sci U S A *105*, 8292-8297.

Hamanoue, M., Morioka, K., Ohsawa, I., Ohsawa, K., Kobayashi, M., Tsuburaya, K., Akasaka, Y., Mikami, T., Ogata, T., and Takamatsu, K. (2016). Cell-permeable p38 MAP kinase promotes migration of adult neural stem/progenitor cells. Scientific reports *6*, 24279.

Hedges, J.C., Dechert, M.A., Yamboliev, I.A., Martin, J.L., Hickey, E., Weber, L.A., and Gerthoffer, W.T. (1999). A role for p38(MAPK)/HSP27 pathway in smooth muscle cell migration. J Biol Chem *274*, 24211-24219.

Huang, C., Jacobson, K., and Schaller, M.D. (2004). MAP kinases and cell migration. J Cell Sci *117*, 4619-4628.

James, R., Kim, Y., Hockberger, P.E., and Szele, F.G. (2011). Subventricular zone cell migration: lessons from quantitative two-photon microscopy. Front Neurosci *5*, 30.

Kim, Y., Comte, I., Szabo, G., Hockberger, P., and Szele, F.G. (2009). Adult mouse subventricular zone stem and progenitor cells are sessile and epidermal growth factor receptor negatively regulates neuroblast migration. PLoS One *4*, e8122.

Ksiazek, I., Burkhardt, C., Lin, S., Seddik, R., Maj, M., Bezakova, G., Jucker, M., Arber, S., Caroni, P., Sanes, J.R.*, et al.* (2007). Synapse loss in cortex of agrin-deficient mice after genetic rescue of perinatal death. J Neurosci *27*, 7183-7195.

Law, A.J., Wang, Y., Sei, Y., O'Donnell, P., Piantadosi, P., Papaleo, F., Straub, R.E., Huang, W., Thomas, C.J., Vakkalanka, R.*, et al.* (2012). Neuregulin 1-ErbB4-PI3K signaling in schizophrenia and phosphoinositide 3-kinase-p110delta inhibition as a potential therapeutic strategy. Proc Natl Acad Sci U S A *109*, 12165-12170.

Nam, S.C., Kim, Y., Dryanovski, D., Walker, A., Goings, G., Woolfrey, K., Kang, S.S., Chu, C., Chenn, A., Erdelyi, F.*, et al.* (2007). Dynamic features of postnatal subventricular zone cell motility: A two-photon time-lapse study. J Comp Neurol *505*, 190-208.

Rousseau, S., Houle, F., Landry, J., and Huot, J. (1997). p38 MAP kinase activation by vascular endothelial growth factor mediates actin reorganization and cell migration in human endothelial cells. Oncogene *15*, 2169-2177.

Sato, K., Hamanoue, M., and Takamatsu, K. (2008). Inhibitors of p38 mitogen-activated protein kinase enhance proliferation of mouse neural stem cells. J Neurosci Res *86*, 2179-2189.

Snel, B., Lehmann, G., Bork, P., and Huynen, M.A. (2000). STRING: a web-server to retrieve and display the repeatedly occurring neighbourhood of a gene. Nucleic Acids Res *28*, 3442-3444.

Sun, B., Chang, E., Gerhartl, A., and Szele, F.G. (2018). Polycomb Protein Eed is Required for Neurogenesis and Cortical Injury Activation in the Subventricular Zone. Cereb Cortex *28*, 1369-1382.

Sun, X.D., Li, L., Liu, F., Huang, Z.H., Bean, J.C., Jiao, H.F., Barik, A., Kim, S.M., Wu, H., Shen, C.*, et al.* (2016). Lrp4 in astrocytes modulates glutamatergic transmission. Nat Neurosci *19*, 1010-1018.

Szklarczyk, D., Morris, J.H., Cook, H., Kuhn, M., Wyder, S., Simonovic, M., Santos, A., Doncheva, N.T., Roth, A., Bork, P.*, et al.* (2017). The STRING database in 2017: quality-controlled protein-protein association networks, made broadly accessible. Nucleic Acids Res *45*, D362-D368.

Ward, M.E., and Rao, Y. (2005). Investigations of neuronal migration in the central nervous system. Methods Mol Biol *294*, 137-156.

Yan, F., Tan, X., Wan, W., Dixon, B.J., Fan, R., Enkhjargal, B., Li, Q., Zhang, J., Chen, G., and Zhang, J.H. (2017). ErbB4 protects against neuronal apoptosis via activation of YAP/PIK3CB signaling pathway in a rat model of subarachnoid hemorrhage. Exp Neurol *297*, 92-100.

Zhu, G., Chow, L.M., Bayazitov, I.T., Tong, Y., Gilbertson, R.J., Zakharenko, S.S., Solecki, D.J., and Baker, S.J. (2012). Pten deletion causes mTorc1-dependent ectopic neuroblast differentiation without causing uniform migration defects. Development *139*, 3422-3431.

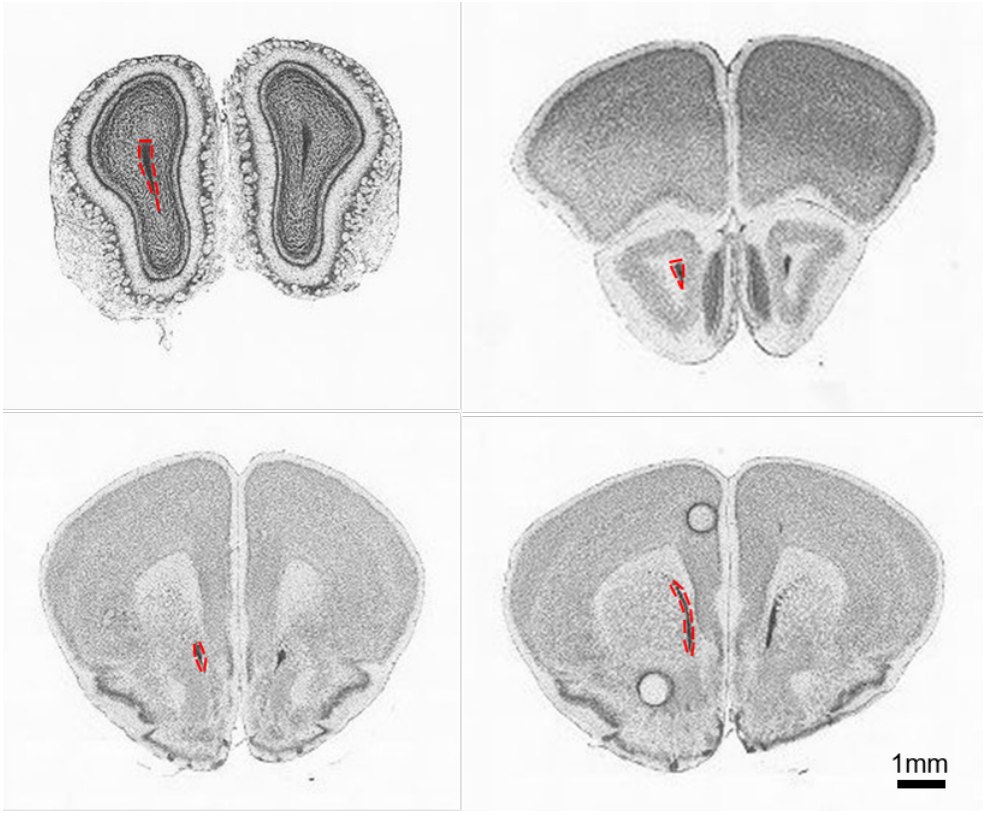

Figure S1: Diagrammatic representation of the regions dissected for the RMS spheroid assay. Nissl stain of representative coronal sections. The RMS outlined in red is 'speckled' in appearance and can be traced back from the centre of the olfactory bulb caudally to the lateral ventricle in a tear drop shape. We avoided dissecting out the anterior commissure which is white in appearance and could be mistaken for the RMS. The rostral half of the olfactory bulb was also excluded as it contains fewer migrating neuroblasts. It was also important not to dissect the SVZ once the ventricle appears to avoid contamination with ciliated ependymal cells, as these may obstruct spheroid formation. Scale bar = 1 mm.

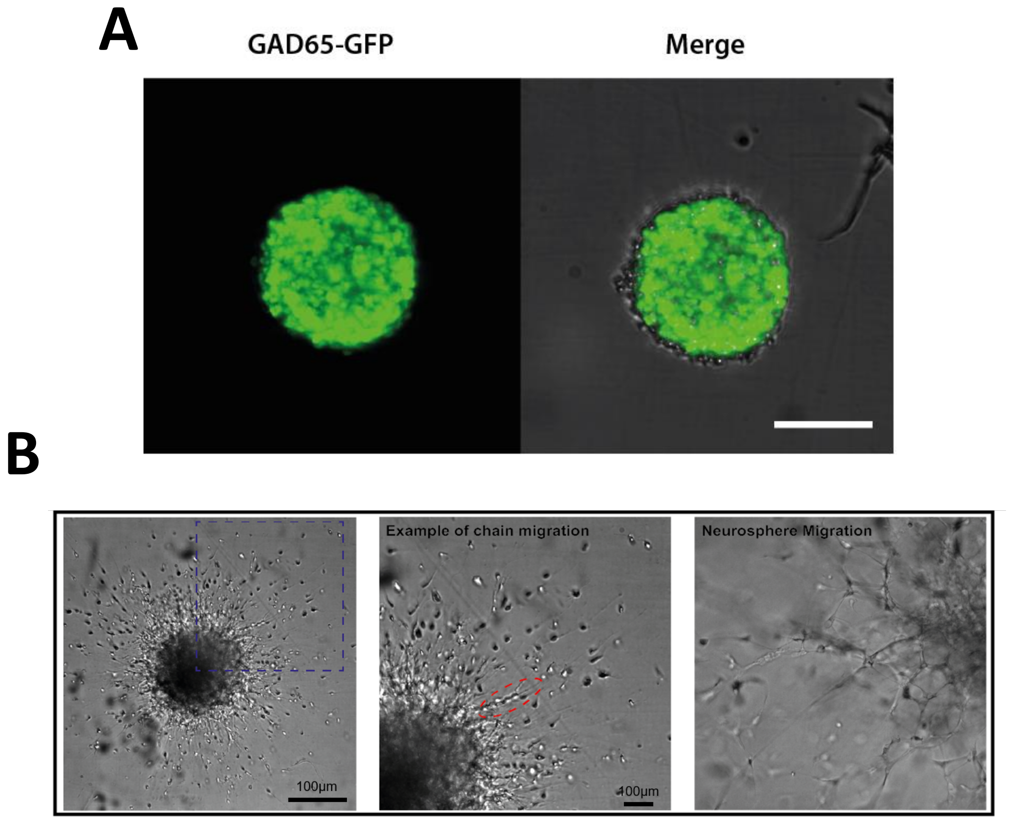

Figure S2.
A: Development of the 3D SVZ spheroid assay using Gad65-GFP mice. Spheroid formation occurred within 24 hours and spheroid shape and size was determined by imaging GFP-labelled cells once transferred onto Matrigel. Right panel shows GFP fluorescence overlap with brightfield. Scale bar = 100 μm.
B: Magnified image of the chain and single cell migration of neuroblasts from an RMS spheroid. Left and central image show cells migrating out as chains from an RMS spheroid. Neuroblasts self-organise into chains to radiate out from the spheroid before continuing as single cells. Scale bars = 100 μm. The right image shows the amoebic glial migration that radiates from a neurosphere as a comparison.

A

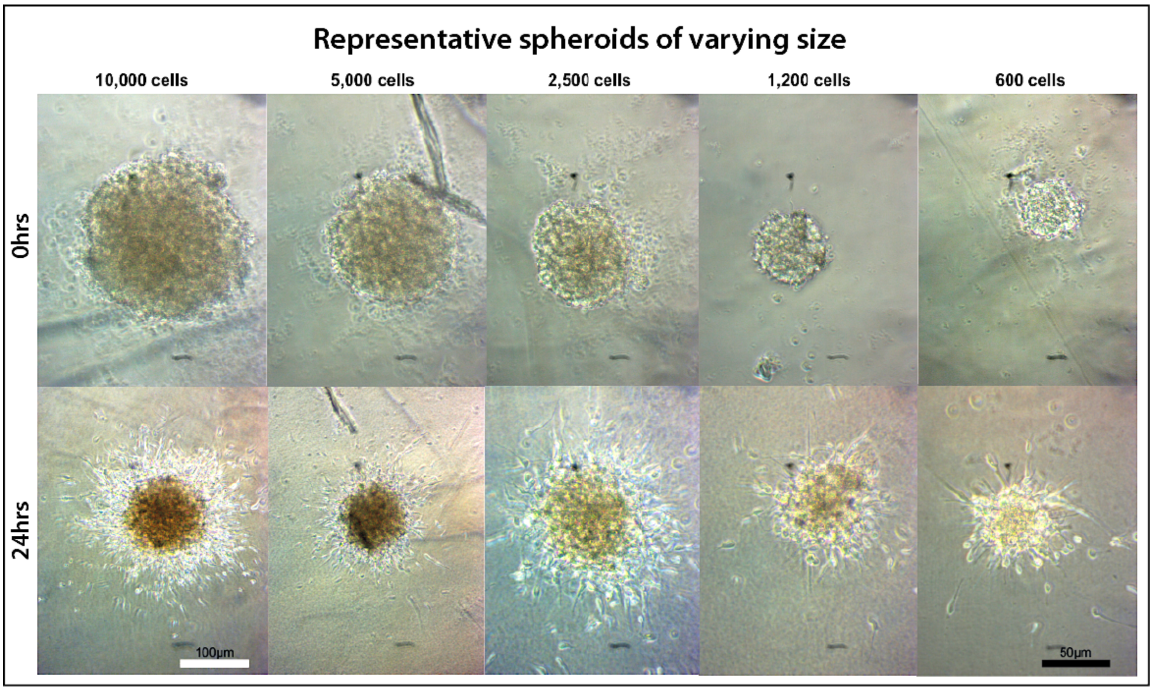

B

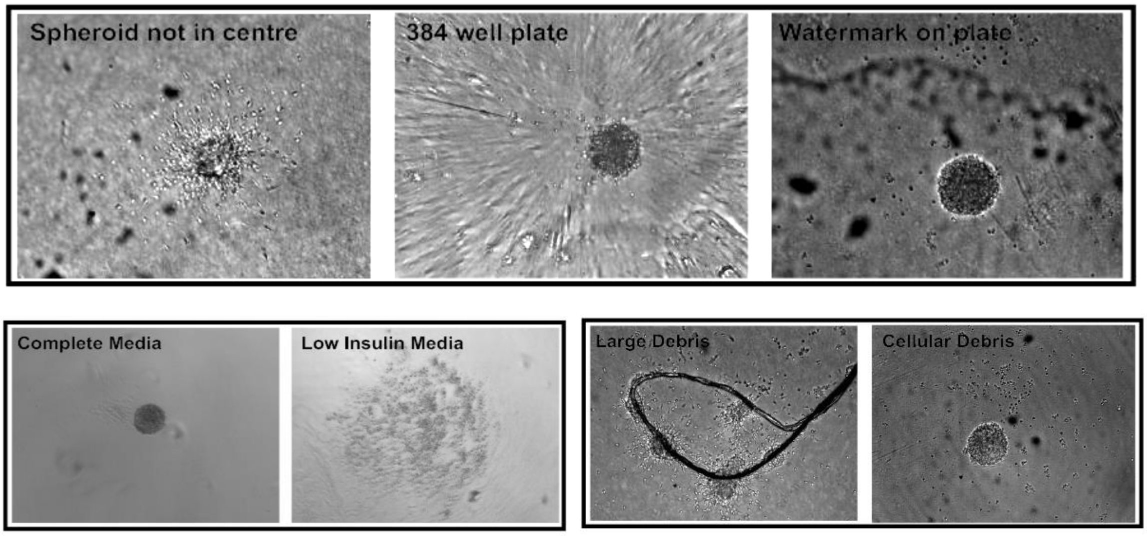

Figure S3.

A: Spheroid size optimisation. Spheroids of between 600 and 10,000 cells were tested for suitability in the 3D assay with imaging at 0 and 24 hours. Larger spheroids showed more migration after 24 hours, but were too large to measure cells migrating the greatest distances. Therefore, intermediate sized spheroids were selected for high throughput screening. The white (100 µm) scale bar on the 5,000 and 10,000 cells reflects a change from a 20x to 10x objective. The black (50 µm) scale bar applies to all other images.

B: Optimising spheroid assay for media composition, debris levels, spheroid positioning and plate suitability. The success of the spheroid assay was dependent on a number of components to ensure spheroid integrity and imaging resolution. These included assessment of the impact on imaging of: spheroids not centred, 384 well plates, watermark on plate, complete media, low insulin media, large debris and cellular debris.

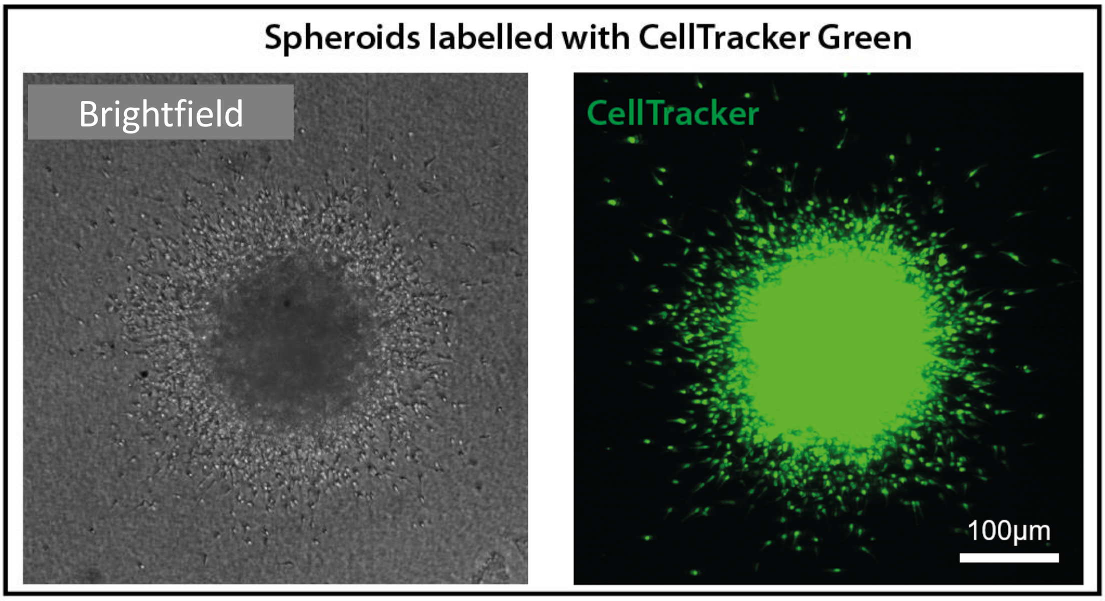

Figure S4. Spheroids labelled with fluorescence dyes. CellTracker Green allows for easy visualisation of spheroids and migrating neuroblasts. Scale bar = 100 µm.

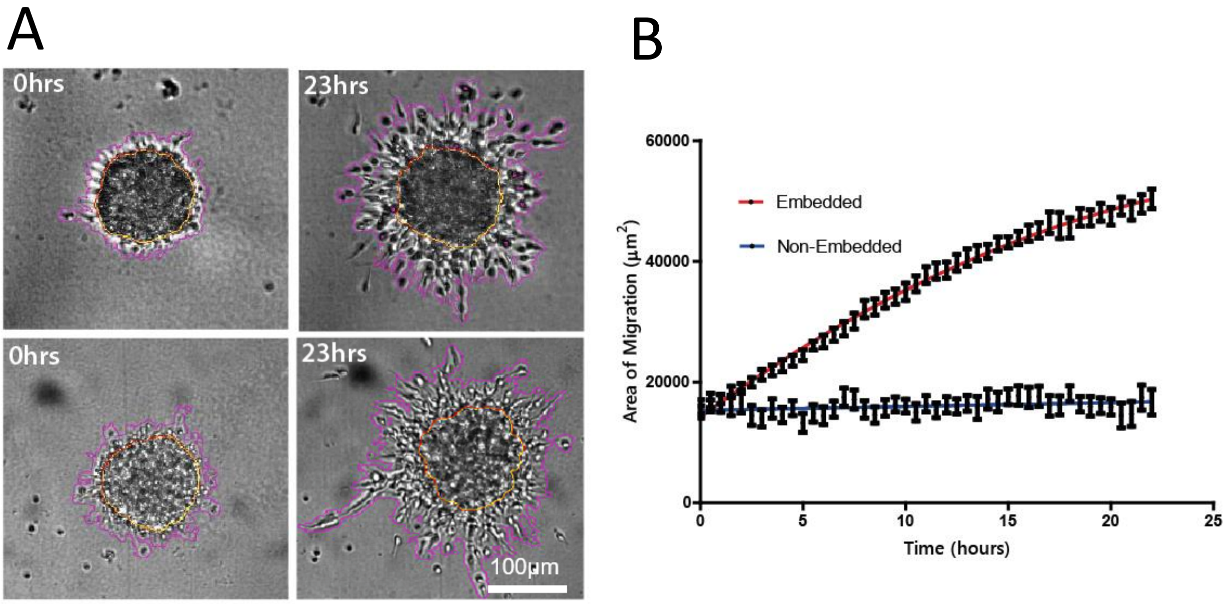

Figure S5: **Segmentation**.
A: Two examples of segmentation of migration area (purple line) around the spheroid (orange line) at 0hrs and 23hrs. Scale bar = 100 µm.
B: The graph shows how the area of migration around 10 spheroids embedded in Matrigel and 10 unembedded spheroids changes over 24 hours.

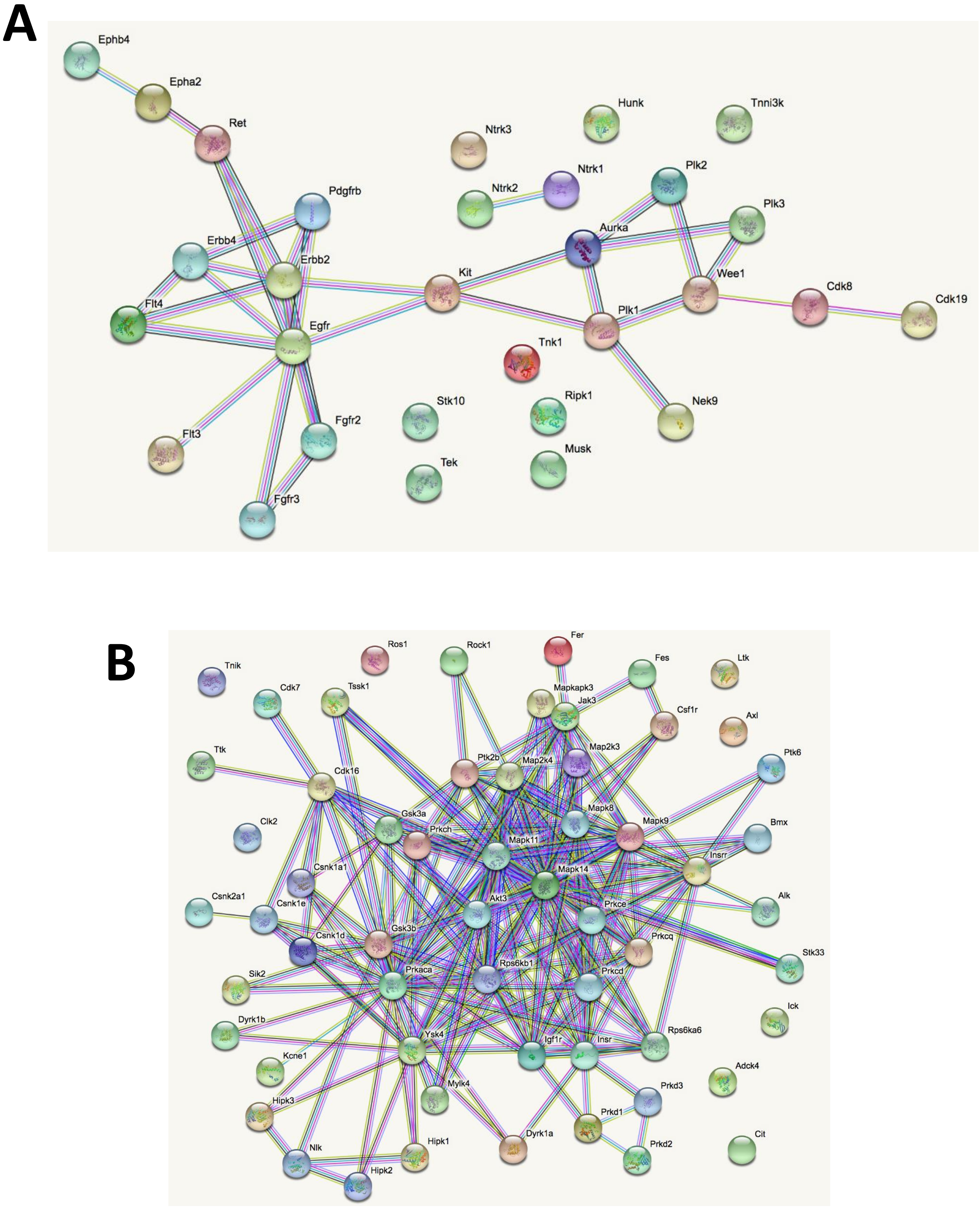

Figure S6

A: STRING representation of kinase targets that increased migration in the PKIS screens

B: STRING representation of kinase targets that decreased migration in the PKIS screens.

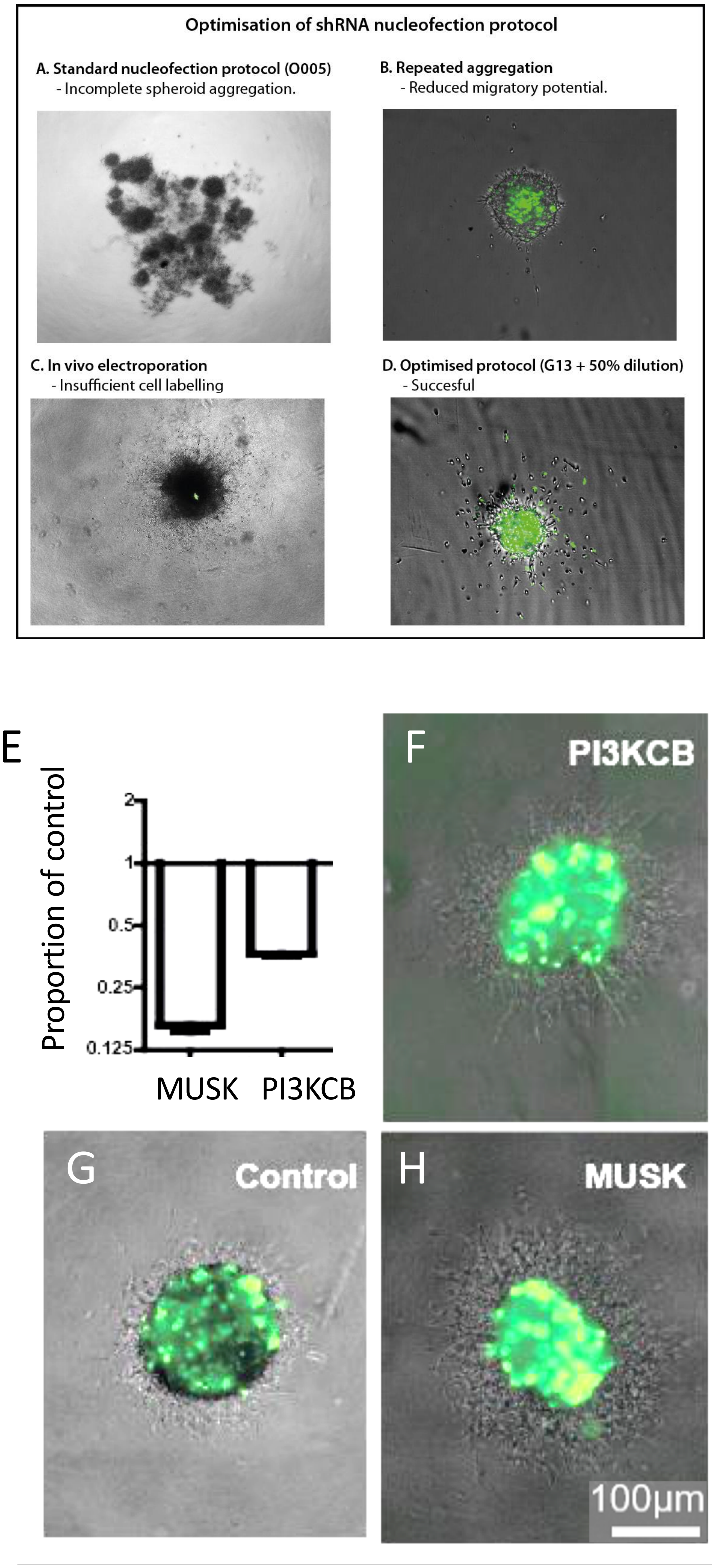

Figure S7

A-D: Optimization of spheroid nucleofection. A-C: Scenarios which were suboptimal. D: Scenario which worked well.

E-H: Knockdown of kinases MuSK and PIK3CB (E). Migration compared to controls increased after both MuSK (H) and PIK3CB KD (F). F-H scale bar = 100 µm.

Table S1. Top kinases selected from analysis of the expression ratio between high and low migrating conditions.

Kinases with highest Δ high:low

PKIS1

Gene	Mean Affinity		Δ high:low
	High	Low	
LOK	25.9	11.9	14.0
FLT3	21.9	8.1	13.8
TRKC	21.8	8.0	13.8
PDGFRα	26.6	13.2	13.4
AURORA-C	21.3	8.5	12.8
TRKB	20.4	7.8	12.6
KIT	18.2	7.4	10.8
TRKA	16.1	6.1	10.0
AURORA-A	18.8	10.9	7.9
EGFR	22.1	14.4	7.7
FGFR2	13.8	6.4	7.4
ERBB4	27.6	20.4	7.2
AURORA-B	15.7	8.5	7.1
RET	21.2	14.6	6.6
FLT4	16.5	10.0	6.5
MUSK	15.5	9.1	6.4
FGFR3	11.4	5.1	6.2
TIE2	16.5	10.4	6.1
NEK9	10.9	5.2	5.7
TNK1	20.3	15.2	5.1
PLK1	13.2	8.2	5.0

PKIS2

Gene	Mean Affinity		Δ high:low
	High	Low	
ERBB2	32.8	20.1	12.7
RIPK1	18.4	8.3	10.0
PLK2	17.2	7.8	9.4
PLK1	15.2	6.3	8.9
MUSK	16.3	7.4	8.9
CDK11	18.4	10.1	8.3
PLK3	13.8	6.7	7.1
EPHB4	16.6	10.0	6.6
CDK8	26.0	19.4	6.6
EGFR	36.8	30.5	6.3
EPHA2	18.1	12.3	5.8
TNNI3K	20.5	14.9	5.6
WEE1	10.5	5.5	5.0
HUNK	14.4	9.4	5.0

Kinases with lowest Δ high:low

PKIS1

Gene	Mean Affinity		Δ high:low
	High	Low	
P38α	11.6	46.3	-34.7
CK1a	4.5	27.8	-23.3
P38β	8.4	27.0	-18.6
PYK2	10.3	26.3	-16.1
JNK2	6.0	20.9	-14.9
INSR	4.6	19.1	-14.6
LTK	4.2	18.1	-14.0
IGF1R	2.7	15.5	-12.9
GSK3B	12.0	24.4	-12.5
FER	4.9	17.2	-12.3
PRKD2	4.6	16.9	-12.3
MAPKAI	4.9	16.9	-12.0
GSK3A	12.3	23.4	-11.1
ALK	5.2	16.1	-10.9
PRKD3	4.0	14.8	-10.8
PRKD1	4.8	15.4	-10.6
ROS	11.6	21.8	-10.2
DYRK1A	7.1	17.1	-10.0
IRR	1.5	11.4	-9.9
CK1-g3	3.1	11.9	-8.9
TSSK1	2.9	11.2	-8.3
DYRK1E	4.0	12.3	-8.3
PKA	2.7	10.7	-8.1
FES	2.6	10.2	-7.7
TTK	10.4	17.1	-6.7
CLK2	7.5	14.2	-6.7
BMX	6.3	12.6	-6.4
CK1-g1	3.2	8.9	-5.7
BRK	9.0	14.4	-5.4
SNF1LK	9.1	14.5	-5.3
MINK	19.2	24.5	-5.2

PKIS2

Gene	Mean Affinity		Δ high:low
	High	Low	
CSNK1A1	11.5	39.9	-28.4
CSNK1E	25.8	51.3	-25.5
CSNK1D	21.5	44.5	-23.0
MEK3	8.3	29.2	-20.9
CSNK1A1	7.2	27.6	-20.4
PRKCH	8.3	28.4	-20.1
HIPK1	8.1	27.1	-19.0
PRKCD	5.0	23.7	-18.7
YSK4	34.1	52.7	-18.6
ROCK1	16.0	33.5	-17.5
HIPK2	5.2	22.7	-17.5
DYRK1B	7.8	24.7	-16.9
HIPK3	12.2	28.9	-16.8
PRKCE	11.4	27.8	-16.4
TNIK	20.4	36.3	-15.9
S6K1	8.3	23.3	-15.0
CSF1R	18.7	33.5	-14.8
AKT3	4.5	19.2	-14.7
CSNK2A1	6.3	20.9	-14.5
MINK	25.8	40.2	-14.4
CIT	31.9	46.3	-14.4
JAK3	13.0	27.3	-14.3
GSK3A	14.2	28.4	-14.2
PCTK1	4.5	18.7	-14.2
MYLK4	13.5	27.6	-14.1
AXL	17.8	31.6	-13.9
STK33	10.3	24.1	-13.8
CDK7	9.9	23.7	-13.7
PKAC-alph	7.1	20.7	-13.6
RSK4	19.1	32.6	-13.5
ADCK4	21.0	34.5	-13.5
PRKCQ	12.7	26.2	-13.5
PRKD1	14.3	27.6	-13.3
MEK4	18.0	31.3	-13.3
ICK	10.8	24.1	-13.3
JNK1	21.7	34.9	-13.2
NLK	22.6	35.6	-13.0

Table S2. Compounds that significantly altered migration from PKIS1.

Compounds that stimulated migration (PKIS1)

Compound	x Control	>90	>80	ABL1	BRK	SRC	YES	EPHA2	EPHA4	DDR2	MUSK	TRKA	TRKC	RET	KDR	KIT	FLT3	PDGFRα	PDGFRβ	TIE2	EGFR	ERBB4	MAP4K4	LOK	PLK1	AURORA-C	NEK9
GW607049C	3.03	0	4	72	1	13	6	90	82	83	79	83	70	74	47	44	33	58	17	71	3		7	73	1	6	0
GW703087X	2.70	0	0	2	2	6	2	2	1	8	7	0	2	11	13	2	2	15	7	7	77	57	3	4	3	15	2
SB-735467	2.43	0	0	69	3	6	7	-2	4	2	5	4	7	4	19	5	4	15	8	7	4	12	70	9	2	7	6
GW300653X	2.39	0	0	7	1	14	9	-2	5	0	4	26	35	34	4	32	44	34	30	3	2	2	62	2	1	7	5
GSK317315A	2.27	1	3	1	-1	0	1	-4	0	-2	22	3	6	4	14	15	8	35	37	5	3	3	4	81	99	0	84
GW627512B	2.23	3	4	22	4	72	47	-1	1	9	14	23	25	16	48	99	91	70	74	5	14	-3	26	11	2	72	8
GSK237701A	2.22	5	9	58	4	41	18	7	27	12	78	12	63	52	72	27	25	100	95	42	3	14	19	88	97	51	92
GW627834A	2.04	0	0	10	3	20	18	1	8	3	3	13	25	4	11	80	62	17	26	2	2	9	24	4	2	6	5
GW804482X	1.99	1	1	1	1	-3	1	2	3	0	-4	-8	-2	7	3	-4	-4	14	8	3	2	13	18	39	92	4	8
GSK259178A	1.96	0	1	4	5	9	8	0	3	3	5	1	3	5	11	2	3	12	8	11	76	86	6	8	11	9	12
GW824645A	1.95	0	1	9	4	10	12	1	10	3	14	19	28	18	15	85	67	36	38	5	3	11	70	16	3	0	9
GW794607X	1.94	0	7	86	51	82	83	5	17	8	50	7	37	55	65	24	18	78	79	36	33	43	44	28	1	59	0
GW701427A	1.94	0	0	15	0	8	2	-5	5	49	3	3	4	14	13	19	6	39	16	7	-2	0	6	7	1	38	2
SB-437013	1.93	0	1	12	42	9	8	2	8	4	3	2	2	9	18	-1	2	10	4	83	3	23	8	78	4	15	0
GW683134A	1.91	7	19	94	4	37	22	83	87	80	83	85	90	84	72	92	71	85	58	82	-1	-5	5	77	1	82	7
GW693881A	1.90	0	1	5	6	7	9	-6	6	-1	6	3	2	12	12	2	2	16	9	9	77	69	14	12	4	-2	9
SB-633825	1.88	1	2	43	85	11	21	1	18	-5	6	1	4	22	48	5	1	24	13	79	6	44	15	95	4	13	18
GSK300014A	1.86	0	1	5	6	15	6	-1	10	6	9	7	4	7	9	8	7	14	11	11	84	77	10	13	8	9	9
GW780159X	1.83	0	0	8	11	14	18	1	15	-6	2	0	5	6	67	8	2	28	15	6	7	28	60	14	4	-2	12

Compounds that inhibited migration (PKIS1)

Compound	x Control	>90	>80	YES	PYK2	LTK	ALK	INSR	IGF1R	DDR2	PDGFRβ	ERBB4	CK1a	PRKD3	PRKD2	TSSK1	CDK2	CDK3	JNK2	P38α	GSK3A	GSK3B	CLK2	DYRK1A	TTK
GW784752X	0.34	6	19	73	16	6	8	7	5	9	81	21	11	9	9	5	77	49	11	16	90	89	35	72	19
SB-686709-A	0.33	0	2	0	1	0	2	1	-1	1	5	1	-1	0	0	-1	20	17	2	-1	90	89	3	61	2
GW811168X	0.32	5	10	57	6	2	-2	1	-5	8	78	8	6	-2	-3	-1	68	21	5	5	81	79	18	64	-19
GW827099X	0.30	5	15	97	44	6	8	18	3	83	70	95	73	48	52	3	4	-3	85	93	60	76	1	3	19
SB-253228	0.30	0	1	0	12	2	2	0	-5	2	11	5	15	6	4	3	-1	-1	4	84	3	3	0	0	14
SB-678557-A	0.30	0	2	3	15	5	7	5	4	1	8	24	4	5	6	7	6	6	6	5	86	84	2	34	15
SB-226879	0.30	0	1	9	18	5	5	3	5	3	21	12	73	11	15	7	-1	0	37	86	5	7	2	4	2
SB-253226	0.28	0	0	3	7	4	5	4	6	2	10	14	21	5	5	5	3	-2	6	71	3	2	0	4	15
GW833373X	0.28	2	10	92	48	3	6	8	5	83	68	89	71	81	87	0	1	3	81	89	45	67	2	5	6
GSK1000163A	0.28	0	0	6	-7	0	0	0	-1	6	12	0	-1	6	7	10	-1	-1	-2	0	22	19	1	2	5
GSK1392956A	0.27	3	7	12	97	89	70	117	93	1	5	16	13	21	17	85	4	-3	57	10	0	-1	83	13	82
SB-220025-A	0.27	1	2	16	4	1	2	5	4	0	26	9	86	26	38	-1	6	-1	7	91	4	6	2	4	0
SB-223133	0.27	2	2	40	2	1	3	0	-1	13	30	46	90	19	23	-2	-5	-1	53	93	6	14	1	6	0
GW829906X	0.26	2	6	92	59	3	4	25	5	47	85	79	59	51	57	4	3	-1	82	74	68	76	3	3	11
SB-220455	0.25	0	1	13	6	5	4	5	3	2	30	19	73	17	16	3	1	0	11	83	7	8	14	6	-8
SB-239272	0.24	0	1	-1	7	2	0	3	-4	2	6	15	31	2	4	2	-2	-2	14	86	-2	-2	-1	-2	6
GW276655X	0.24	1	3	12	20	3	0	7	-2	2	50	14	3	7	9	12	94	89	2	3	67	65	36	81	53
SB-251505	0.24	0	1	7	14	3	4	6	4	4	12	11	32	6	5	4	2	0	13	80	4	4	3	6	8
GSK1751853A	0.23	1	5	1	93	81	82	88	88	-1	5	60	11	8	9	32	2	-1	18	7	2	4	45	10	48
GSK2186269A	0.22	3	7	-2	90	90	85	91	98	1	3	6	2	4	4	19	-2	2	2	4	1	1	44	4	25

Table S3. Compounds that significantly altered migration from PKIS2.

Compounds that stimulated migration (PKIS2)																										
Compound	x Control	>90	>80	AURKA	CDC2L5	CDKL2	EGFR	ERBB2	ERK8	FLT4	GRK1	HUNK	KIT	MEK5	MYLK2	NLK	PDGFRB	PIK3CB	PLK1	PLK2	PLK3	RET	RIOK2	SGK2	SLK	SRPK1	YSK4
GSK306886	2.43	5	5	30	0	19	99	99	0	98	4	57	34	26	3	44	46	0	0	0	0	98	3	0	8	100	0
GW579362	2.40	75	124	100	0	41	55	17	18	100	2	8	100	100	32	98	100	0	0	0	4	100	66	0	99	99	99
GSK175726	2.40	3	4	90	0	32	38	22	0	0	100	0	40	49	11	17	21	40	0	3	1	10	92	0	8	0	0
GSK346294	2.37	2	3	8	0	5	32	15	0	1	0	0	20	18	0	19	22	5	100	96	87	24	0	10	59	20	3
GW466413	2.18	1	4	0	0	5	28	35	8	9	14	93	17	35	0	0	5	0	0	0	0	24	2	0	8	21	11
GSK2336394	2.15	1	1	0	0	22	28	20	0	3	0	6	14	1	0	0	15	98	0	0	0	14	6	1	22	37	26
SB-601273	2.11	138	164	62	41	100	88	93	0	96	6	55	100	100	98	99	100	30	26	27	52	100	29	2	99	50	99
GSK1024304	2.08	3	5	2	0	18	4	28	0	1	0	14	23	32	0	0	31	100	97	90	86	26	80	10	9	29	15
GW681170	2.05	32	38	78	69	98	12	22	95	79	0	7	100	51	0	30	100	5	0	0	0	100	0	1	36	25	87
SB-735464	2.03	0	2	18	23	57	0	21	32	0	14	0	33	31	77	86	67	4	0	37	13	0	40	0	11	36	82
SB-732932	2.00	4	9	13	47	95	0	0	92	0	0	0	56	50	97	46	80	8	2	24	0	0	16	4	0	45	95
GW854278	1.97	5	8	0	5	0	27	11	0	68	0	0	24	99	0	0	58	0	100	100	99	20	14	3	96	44	61
SB-733416	1.97	1	1	0	98	9	37	0	23	27	6	0	8	37	0	11	16	0	0	0	0	23	0	9	28	11	0
GW824645	1.95	3	4	4	0	0	0	4	3	0	23	58	90	70	9	0	96	0	0	20	23	0	0	100	0	0	0
GW683134	1.91	41	54	0	0	85	44	9	82	100	0	0	100	85	75	6	100	0	0	0	0	100	7	14	83	51	93
GW659009	1.81	14	22	0	0	15	100	93	10	0	0	0	17	98	0	0	36	0	0	0	30	0	53	4	0	37	3

Compounds that inhibited migration (PKIS2)																															
Compound	x Control	>90	>80	AMPK-alpha1	AXL	CDKL2	DAPK3	ERK8	FLT3	GSK3A	ICK	IGF1R	LYN	MYLK2	MYLK4	NIM1	PDGFRB	PHKG2	PIK3C2B	PIK3C2G	PIK3CB	PIK3CD	PIK3CG	PLK1	PLK2	PLK3	TXK	VPS34	ZAK	p38-alpha
GSK1440913	0.34	3	4	17	51	89	5	99	48	94	79	0	20	76	100	12	60	15	0	24	0	47	67	0	0	0	35	70	16	0
SB-738481	0.34	5	8	0	37	87	0	84	92	93	95	0	11	89	19	100	90	0	7	0	13	5	12	3	3	12	20	0	15	0
GSK2373693	0.33	5	6	0	4	11	95	26	0	0	15	10	0	6	2	11	65	0	99	89	0	100	94	0	0	0	0	97	15	66
GSK1660437	0.33	3	3	48	68	99	6	66	33	12	0	0	74	0	0	0	99	0	0	0	13	0	0	0	5	0	20	0	97	32
SB-711880	0.32	3	6	90	18	99	21	82	28	87	26	0	0	85	0	0	0	100	14	0	0	0	17	0	0	0	0	0	0	0
SB-229482	0.31	0	1	0	48	22	0	0	27	0	45	0	15	21	11	54	29	16	0	0	37	0	34	11	0	0	0	50	4	89
GW813349	0.30	3	4	0	2	13	0	9	8	17	0	9	33	0	24	5	0	0	24	17	0	0	24	97	92	93	9	84	0	0
GSK1287544	0.29	2	3	17	74	40	0	43	83	0	23	2	0	30	100	15	28	0	0	0	100	0	0	0	14	0	18	28	59	0
GSK1379874	0.28	3	3	0	10	5	0	0	0	0	0	0	0	0	0	33	26	0	0	0	0	0	0	0	0	0	100	16	100	20
GSK1383281	0.28	20	35	57	74	11	37	2	68	7	11	4	0	1	79	0	74	16	0	0	0	0	0	0	13	38	79	0	73	0
SB-219980	0.28	9	11	0	9	0	0	0	0	16	1	0	0	0	0	10	20	0	17	52	4	35	7	12	28	10	0	0	0	100
SB-742034-AC	0.27	1	6	9	13	69	17	2	7	98	22	0	2	89	25	0	30	4	16	13	2	0	4	3	24	5	0	0	0	13
GSK2286295	0.27	4	5	0	100	7	15	2	0	8	3	12	98	2	0	0	37	19	22	0	99	89	50	10	0	0	19	97	6	0
GW494601	0.26	36	53	0	21	2	15	0	28	23	0	13	86	24	0	0	100	0	13	2	0	22	0	30	0	0	98	34	84	50
GSK301362	0.26	7	10	6	47	30	40	0	47	0	14	0	4	40	99	15	67	0	0	0	0	6	2	10	34	0	0	0	0	0
GSK2358994	0.24	2	3	5	3	0	6	13	10	4	4	0	0	0	0	0	2	0	70	64	100	82	45	0	0	16	7	100	12	0
GW560116	0.24	7	9	0	0	2	25	0	0	0	0	24	0	27	5	0	95	0	0	0	0	0	0	0	0	0	23	22	0	36
GSK1581427	0.24	33	54	55	86	18	19	30	68	0	24	0	0	19	100	22	75	19	0	0	0	0	0	0	0	0	1	0	0	0
GSK2373690	0.23	2	2	0	0	24	8	0	0	13	1	41	0	2	100	0	14	0	0	0	97	65	21	0	0	0	3	59	9	0
GW787226	0.23	22	30	17	73	39	0	3	99	0	0	34	84	0	0	0	100	0	5	0	0	0	0	0	0	15	17	0	17	0
SB-219952	0.22	10	11	100	19	0	0	0	24	0	70	0	9	20	21	31	23	0	56	69	0	16	64	23	2	0	16	35	9	95
GSK986310C	0.22	12	29	39	59	28	59	0	23	24	16	0	34	7	94	18	80	44	0	0	0	0	3	1	0	0	0	56	0	0
SB-222903	0.21	6	7	0	0	0	25	6	0	3	0	0	0	0	0	0	17	0	0	0	0	0	0	0	0	4	0	0	0	99
SB-249175	0.21	14	18	1	54	0	0	2	39	0	65	0	12	0	19	44	8	0	23	1	0	18	45	9	24	23	10	24	15	99
GSK2276055	0.20	11	14	0	0	0	23	0	0	98	31	0	0	3	48	30	63	6	9	0	11	3	21	7	18	0	76	0	69	8
GSK1723980	0.20	84	99	74	74	99	0	98	66	74	32	0	98	0	0	0	100	0	7	0	0	0	0	0	0	0	93	0	99	27
SB-710363	0.19	18	27	0	37	97	32	90	82	99	99	0	23	79	31	0	32	0	0	0	0	0	2	0	10	0	0	0	23	0
GSK2008607	0.18	115	145	97	98	99	34	94	70	53	77	8	99	0	0	1	100	0	49	0	0	0	0	0	0	4	95	0	99	81
GSK1389063	0.18	67	100	91	95	40	18	51	98	0	64	0	0	52	99	13	96	24	0	0	52	2	0	0	27	27	30	35	0	0
GSK1383280	0.17	25	41	49	63	0	28	3	69	0	18	23	0	22	86	20	75	20	0	0	0	0	0	0	30	47	0	0	15	11
GSK2181306	0.16	127	170	89	100	44	100	5	61	98	68	19	7	78	60	67	100	94	12	23	100	34	86	31	5	0	26	88	0	0
GSK1379722	0.16	6	6	2	0	30	5	31	1	0	100	100	0	12	0	15	8	0	0	0	0	0	0	0	0	0	16	0	0	6

Table S4. Kinase inhibitors that increased migration of neuroblasts from 3D spheroids. Compounds are listed together with their target (if known), chemical structure, representative image of migration at 24hrs, and the extent of migration above the DMSO control for 5 different concentrations of the compound.

Compound	Chemical Structure	Migration Image	Results
**SB-735467**<br><br>Intended Target:<br><br>**GSK3b**	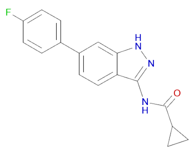	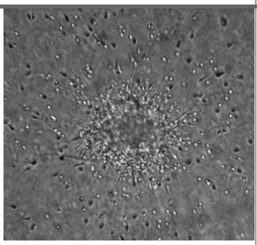	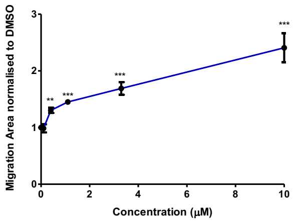
**GW701427A**<br><br>Intended Target:<br>**TIE2/VEGFR2**	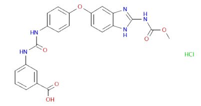	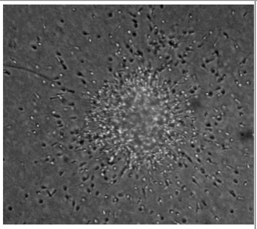	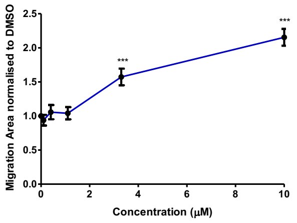
**GW693881A**<br><br>Intended Target:<br>**EGFR/ERBB2**	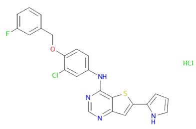	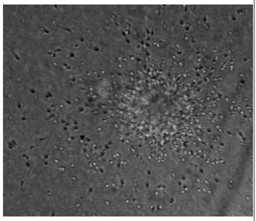	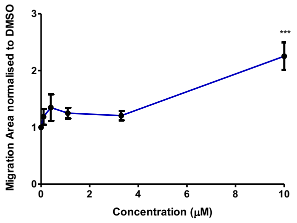
**GW681170A**<br><br>Intended Target:<br>**TIE2/VEGFR**	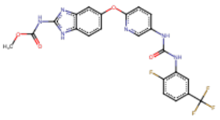	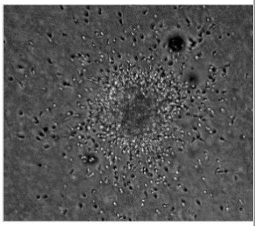	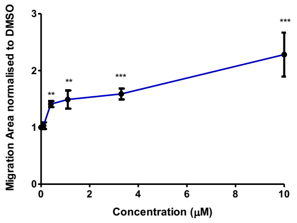
**SB-732932**<br><br>Intended Target:<br>**GSK3a/GSK3b**	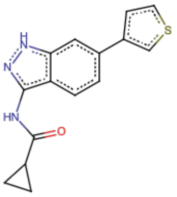	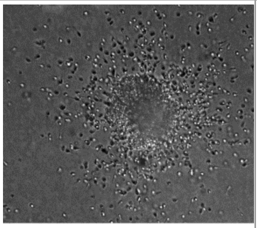	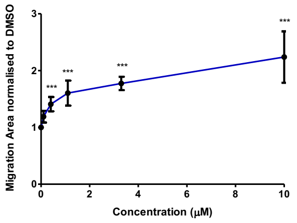
**GW854278X**<br><br>Intended Target:<br>**PLK1/PLK2**	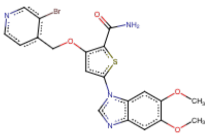	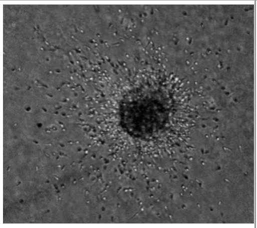	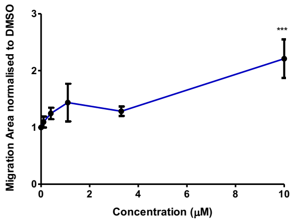

**GW869641X**<br><br>Intended Target:<br>**ALK5**	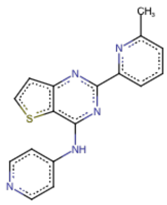	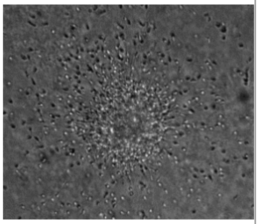	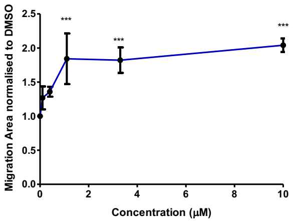
**GW579362A**<br><br>Intended Target:<br><br>**Unknown**	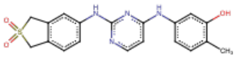	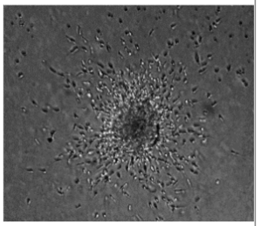	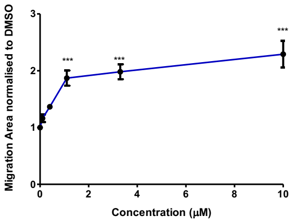
**GW659008A**<br><br>Intended Target:<br>**Unknown**	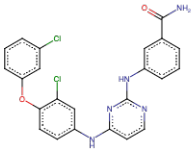	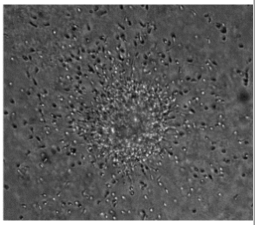	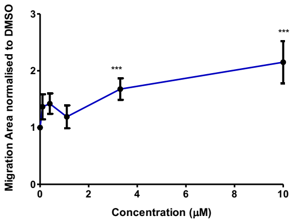
**GW459135A**<br><br>Intended Target:<br>**EGFR/ERBB2**	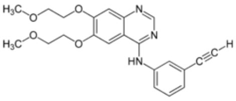	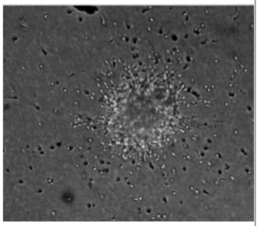	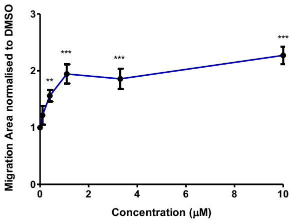
**GW703087X**<br><br>Intended Target:<br>**EGFR**	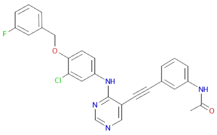	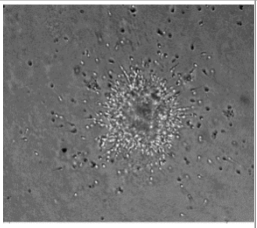	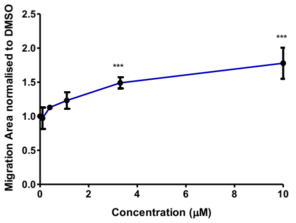
**GW707818B**<br><br>Intended Target:<br>**EGFR/ERBB2**	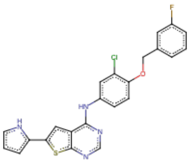	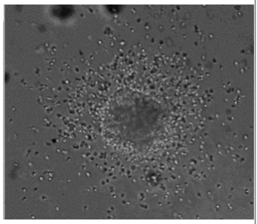	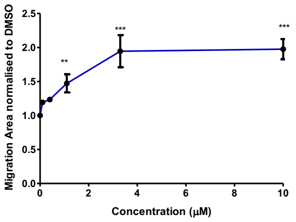
**GSK336313A**<br><br>Intended Target:<br>**Unknown**	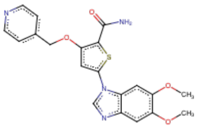	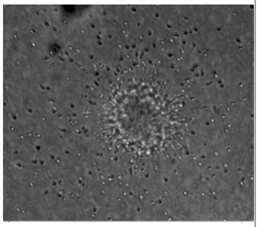	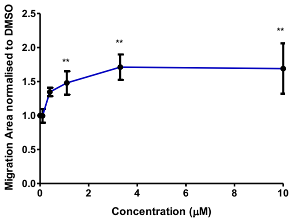

**GSK346294A**<br><br>Intended Target:<br>**PLK1/PLK2**	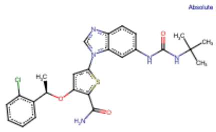	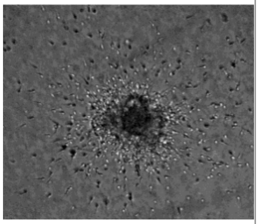	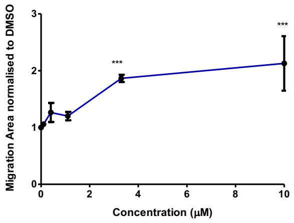
**GSK1024304A**<br><br>Intended Target:<br>**PLK1/PLK2**	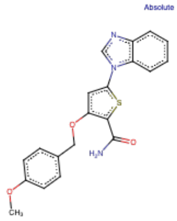	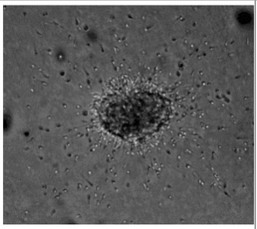	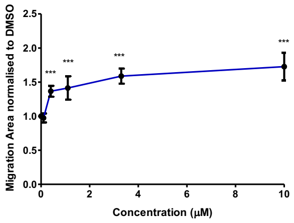
**GSK259178A**<br><br>Intended Target:<br>**EGFR/ERBB2**	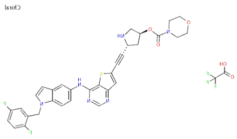	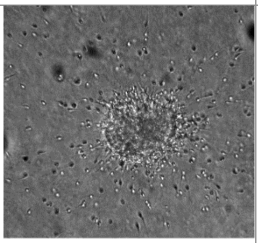	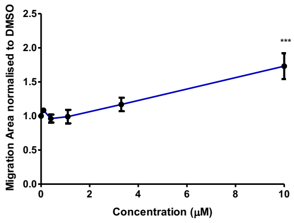
**SB-733416**<br><br>Intended Target:<br>**MSK-1**	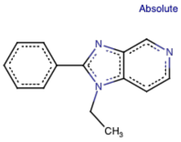	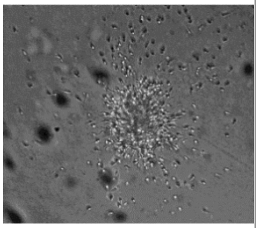	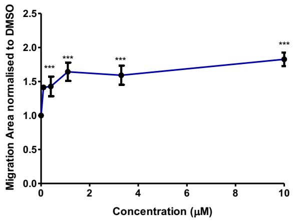
**GW607049C**<br><br>Intended Target:<br>**TIE2/VEGFR2**	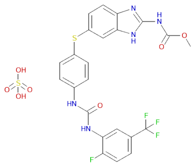	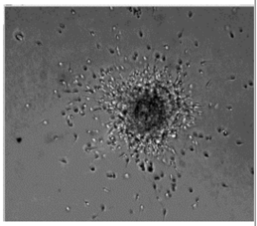	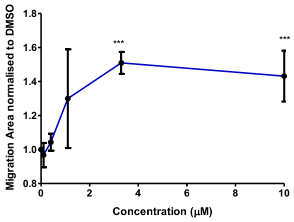
**GW627512B**<br><br>Intended Target:<br>**VEGFR2**	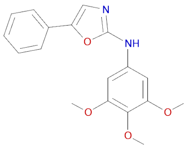	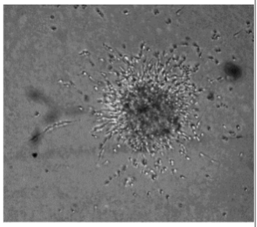	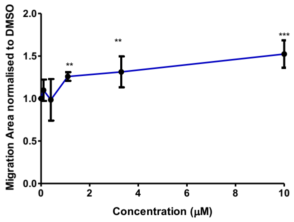
**GSK2336394A**<br><br>Intended Target:<br>**PIK3CB**	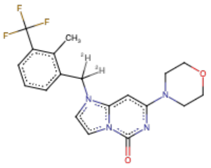	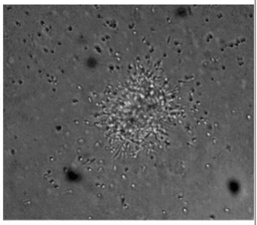	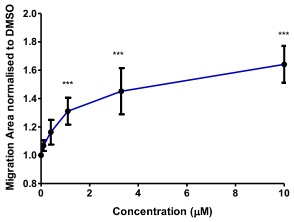

**GW300653X**<br><br>Intended Target:<br>**CDK2**	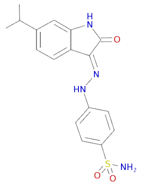	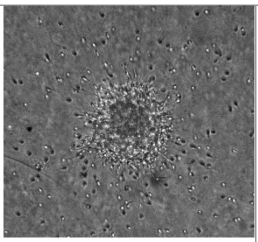	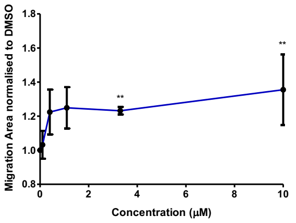

Table S5. Kinase inhibitors that decreased migration of neuroblasts from 3D spheroids. Compounds are listed together with their target (if known), chemical structure, representative image of migration at 24 hours, and distance of migration measured compared to the DMSO control for 5 different concentrations of the compound.

Compound	Chemical Structure	Migration Image	Results
**SB-251505**<br><br>Intended Target:<br>**p38a**	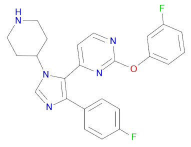	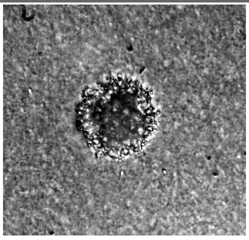	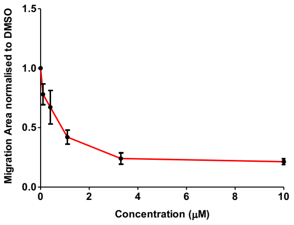
**SB-220455**<br><br>Intended Target:<br>**p38a**	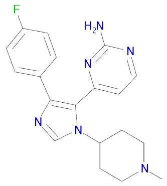	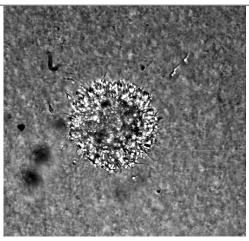	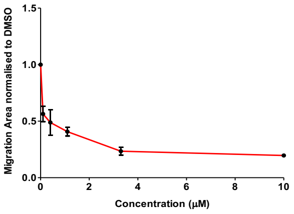
**SB-253228**<br><br>Intended Target:<br>**p38a**	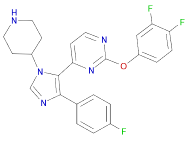	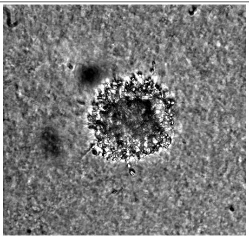	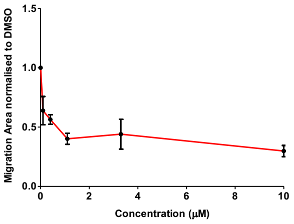
**SB-226879**<br><br>Intended Target:<br>**p38a**	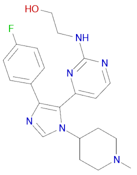	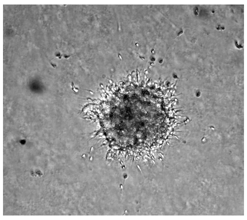	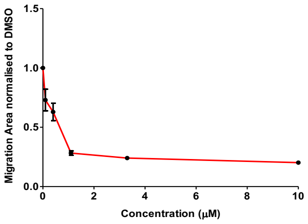
**SB-239272**<br><br>Intended Target:<br>**p38a**	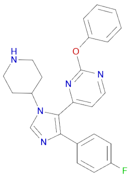	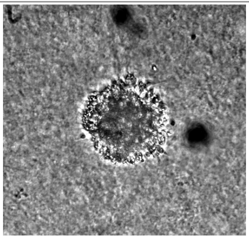	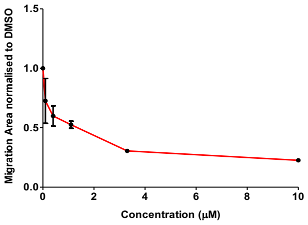
**SB-219980**<br><br>Intended Target:<br><br>**P38a/p38b**	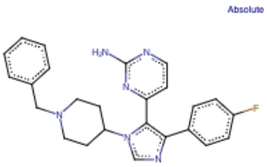	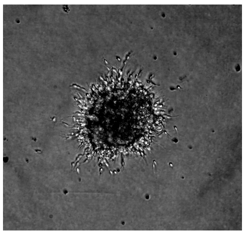	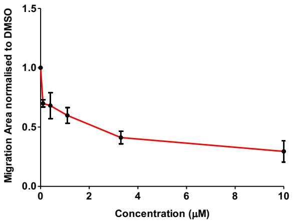

**SB-253226**<br><br>Intended Target:<br>**p38α**	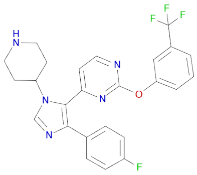	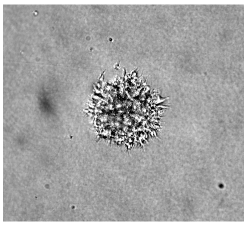	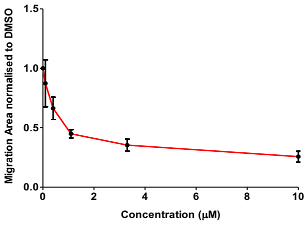
**SB-222903**<br><br>Intended Target:<br><br>**P38a/p38b**	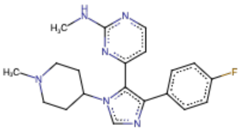	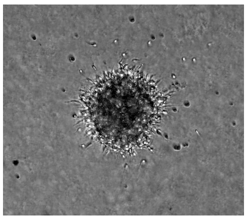	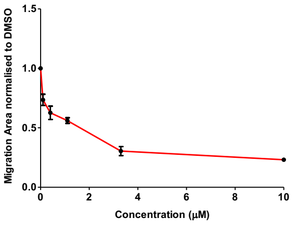
**SB-219952**<br><br>Intended Target:<br><br>**P38a/p38b**	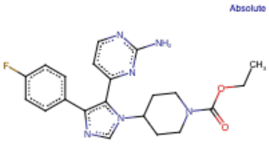	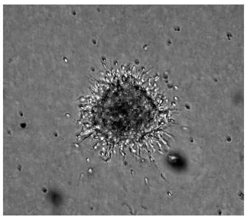	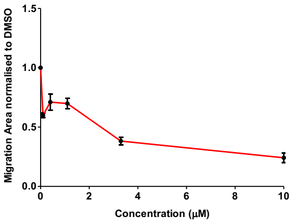
**SB-226605**<br><br>Intended Target:<br><br>**P38a/p38b**	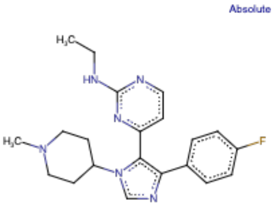	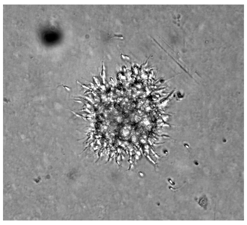	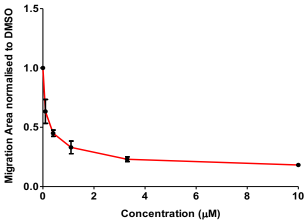
**GW833373X**<br><br>Intended Target:<br>**GSK3b**	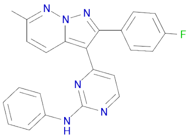	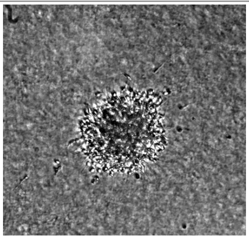	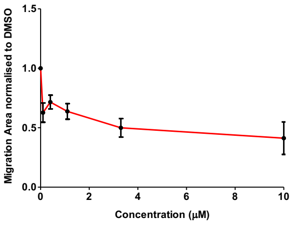
**SB-742034-AC**<br><br>Intended Target:<br><br>**GSK3a/GSK3b**	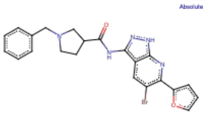	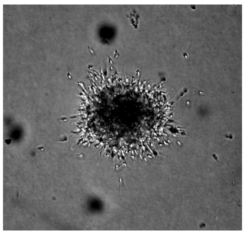	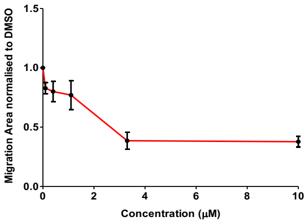
**SB-678557-A**<br><br>Intended Target:<br>**GSK3a/GSK3b**	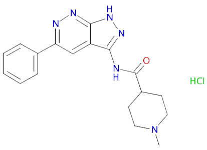	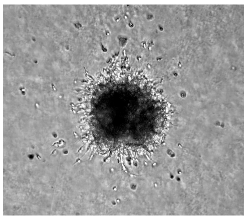	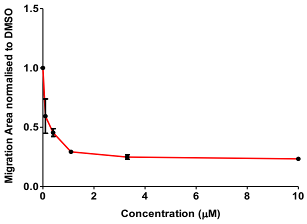

**GW784752X**<br><br>Intended Target:<br><br>**GSK3b**	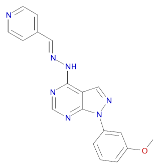	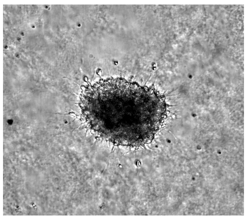	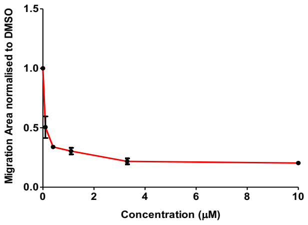
**SB-627772-A**<br><br>Intended Target:<br><br>**GSK3a/GSK3b**	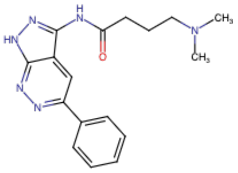	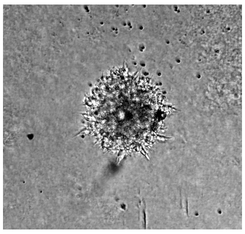	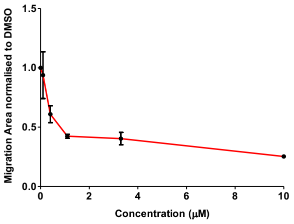
**GW829906X**<br><br>Intended Target:<br>**GSK3b**	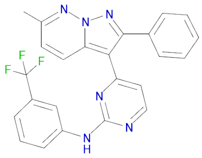	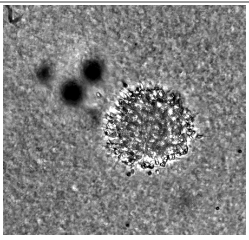	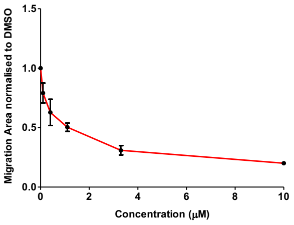
**GSK2358994A**<br><br>Intended Target:<br><br>**PI3K**	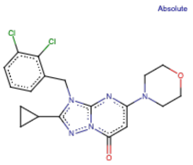	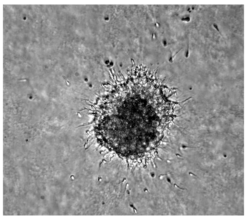	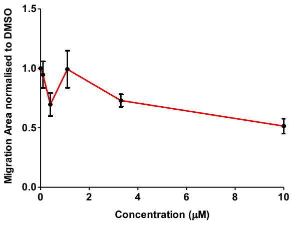
**GSK2286295A**<br><br>Intended Target:<br><br>**PI3K**	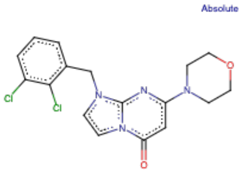	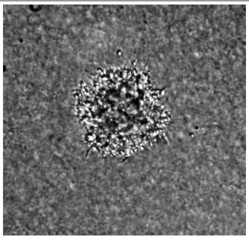	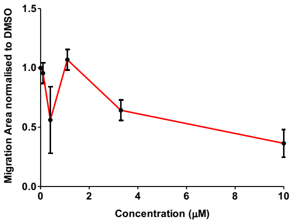
**GSK2373690A**<br><br>Intended Target:<br><br>**PI3K**	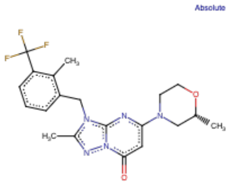	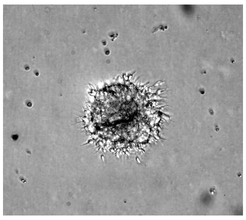	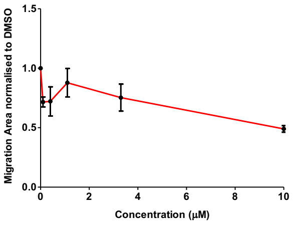
**GSK1389063A**<br><br>Intended Target:<br><br>**Akt**	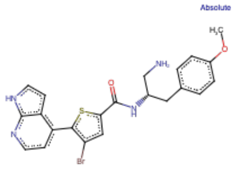	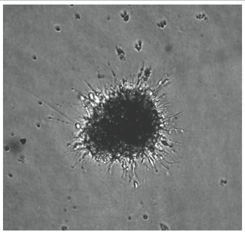	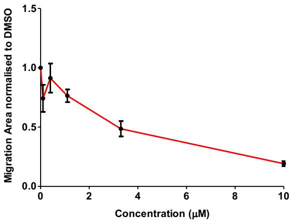

**GSK1000163A**<br><br>Intended Target:<br>**Akt3**	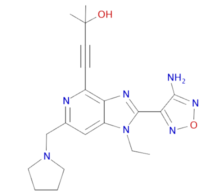	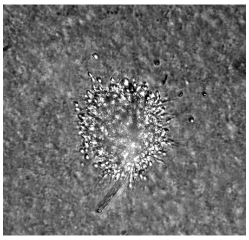	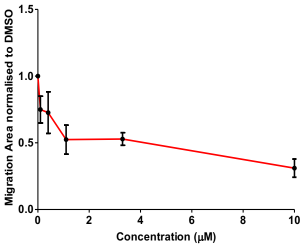
**GSK1383281A**<br><br>Intended Target:<br><br>**Akt**	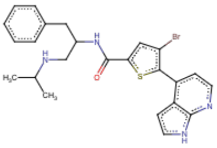	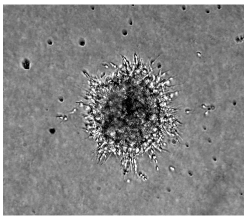	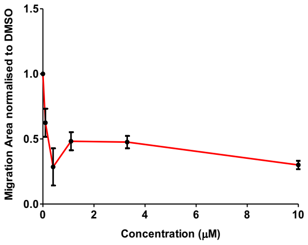
**GSK1383280A**<br><br>Intended Target:<br><br>**Akt**	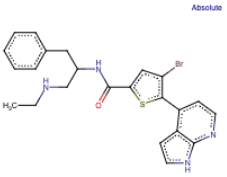	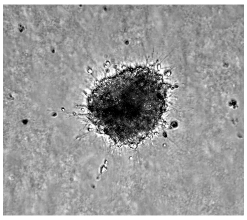	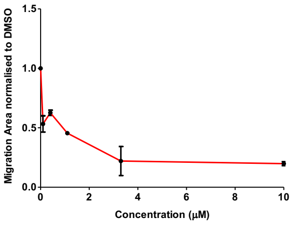
**GW856804X**<br><br>Intended Target:<br><br>**TIE2/VEGFR2**	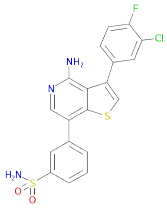	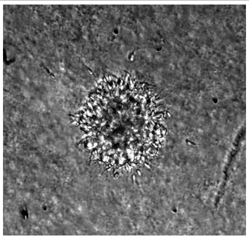	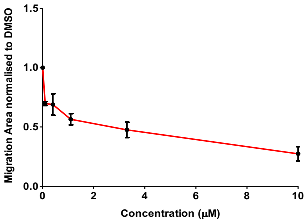
**GW787226A**<br><br>Intended Target:<br><br>**VEGFR**	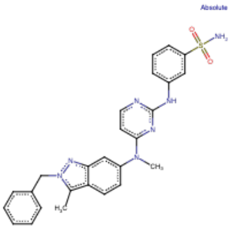	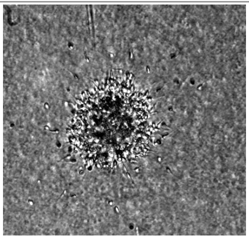	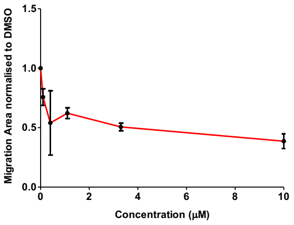
**GSK1819799A**<br><br>Intended Target:<br>**IGF1R**	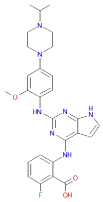	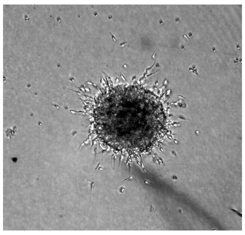	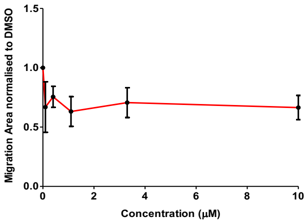
**GW276655X**<br><br>Intended Target:<br>**CDK2**	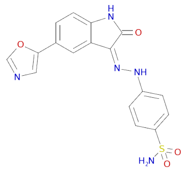	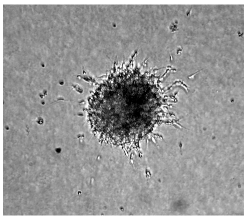	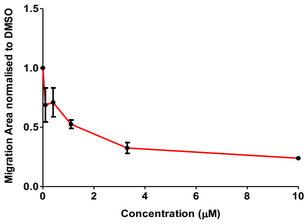

<table>
<tr><td>GSK301362A

Intended Target:

IKK</td><td>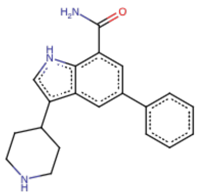</td><td>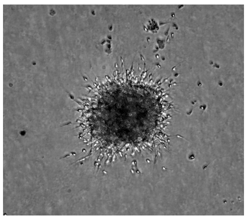</td><td>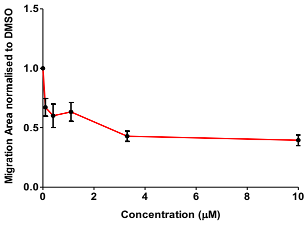</td></tr>
<tr><td>GW813349X

Intended Target:

PLK1/PLK2</td><td>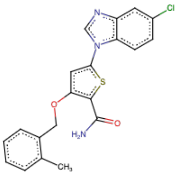</td><td>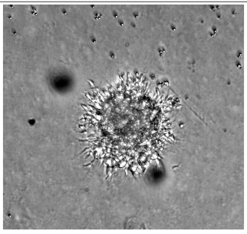</td><td>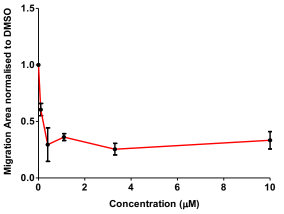</td></tr>
<tr><td>GSK1440913A

Intended Target:

Unknown</td><td>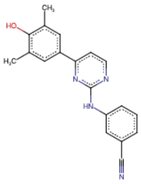</td><td>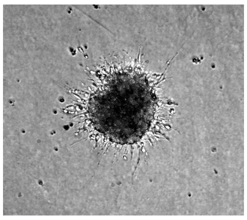</td><td>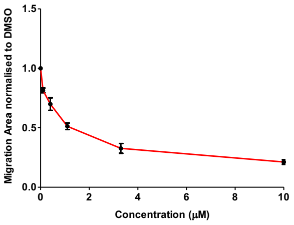</td></tr>
<tr><td>SB-477794-AAA

Intended Target:

Unknown</td><td>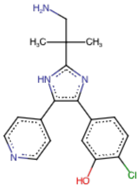</td><td>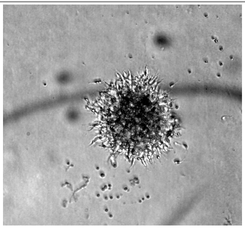</td><td>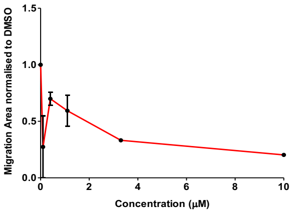</td></tr>
<tr><td>GW494601A

Intended Target:

Unknown</td><td>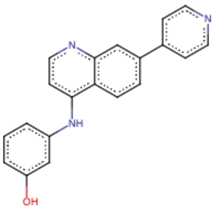</td><td>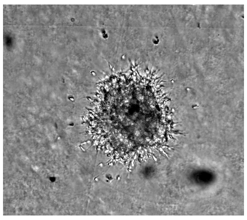</td><td>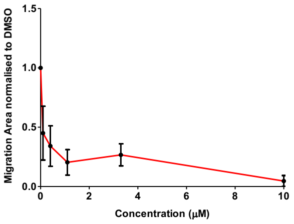</td></tr>
<tr><td>GSK2181306A

Intended Target:

Unknown</td><td>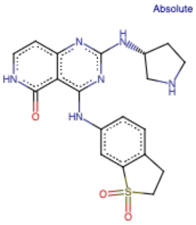</td><td>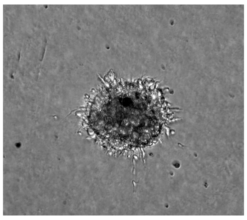</td><td>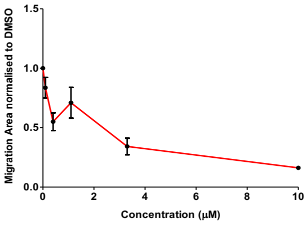</td></tr>
<tr><td>GSK1379874A

Intended Target:

Unknown</td><td>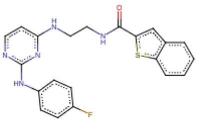</td><td>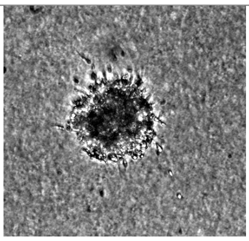</td><td>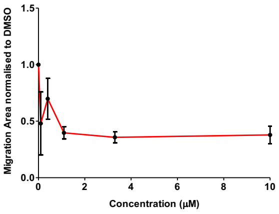</td></tr>
</table>

**GSK1379722A**<br><br>Intended Target:<br><br>**Unknown**	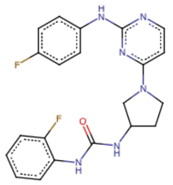	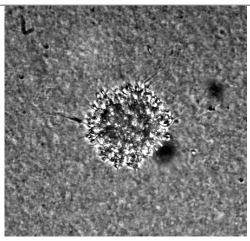	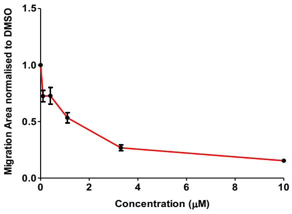
**GW560116X**<br><br>Intended Target:<br><br>**Unknown**	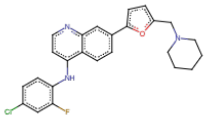	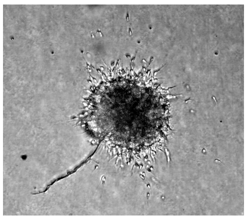	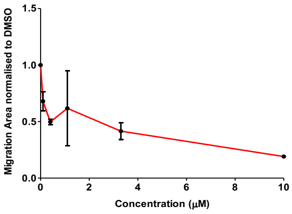
