# Supplementary material for: A Semi-automated and Scalable 3D Spheroid Assay to Study Neuroblast Migration
Source: Stem Cell Reports. 2020 Aug 6;15(3):789–802. doi: 10.1016/j.stemcr.2020.07.012 (PMC7486343; doi:10.1016/j.stemcr.2020.07.012)
Supplement: Document S2. Article plus Supplemental Information [file mmc2.pdf]

Stem Cell Reports
Resource
OPEN ACCESS

A Semi-automated and Scalable 3D Spheroid Assay to Study Neuroblast Migration

Martin Ducker,[1] Valerie Millar,[2] Daniel Ebner,[2] and Francis G. Szele[1,*]
[1]Department of Physiology, Anatomy and Genetics, University of Oxford, South Parks Road, Oxford OX1 3QX, UK
[2]Target Discovery Institute, Nuffield Department of Medicine, University of Oxford, Oxford, UK
*Correspondence: francis.szele@dpag.ox.ac.uk
https://doi.org/10.1016/j.stemcr.2020.07.012

SUMMARY

The subventricular zone of the mammalian brain is the major source of adult born neurons. These neuroblasts normally migrate long distances to the olfactory bulbs but can be re-routed to locations of injury and promote neuroregeneration. Mechanistic understanding and pharmacological targets regulating neuroblast migration is sparse. Furthermore, lack of migration assays limits development of pharmaceutical interventions targeting neuroblast recruitment. We therefore developed a physiologically relevant 3D neuroblast spheroid migration assay that permits the investigation of large numbers of interventions. To verify the assay, 1,012 kinase inhibitors were screened for their effects on migration. Several induced significant increases or decreases in migration. *MuSK* and *PIK3CB* were selected as putative targets and their knockdown validated increased neuroblast migration. Thus, compounds identified through this assay system could be explored for their potential in augmenting neuroblast recruitment to sites of injury for neuroregeneration, or for decreasing malignant invasion.

INTRODUCTION

Neurogenesis is not a single event but a complex multistage process encompassing the proliferation of neural stem cells, their survival, migration, differentiation, and functional integration into the developed neuronal circuitry (Ming and Song, 2005). The rodent subventricular zone (SVZ) lies next to ependymal cells lining the wall of the lateral ventricles. Adjacent to the ependymal cells are quiescent stem cells that occasionally divide symmetrically to produce two transit amplifying progenitors, which divide several times before giving rise to highly motile neuroblasts. Neuroblasts characteristically express the polysialylated form of the neural cell adhesion molecule and the early neuronal marker doublecortin (DCX) (Alvarez-Buylla and Garcia-Verdugo, 2002). Neuroblasts in the rodent brain initiate the migratory route by establishing chains of migration along the rostral migratory stream (RMS), the route to the olfactory bulb (OB). The cells detach from the RMS in the OB, migrate radially to the outer layers, and terminally differentiate into different subtypes of interneurons, with six independent cell types characterized, dependent on their origin along the axes of the SVZ (Merkle et al., 2007). A few molecules, such as galectin-3 (Comte et al., 2011), DCX (Koizumi et al., 2006), and prokineticin (Ng et al., 2005) are known to mediate RMS neuroblast migration. Yet, overall little is known about mechanisms regulating migration compared with other functions of the SVZ.

Pharmacologically modulating the migration of neuroblasts has the potential to improve the prognosis of multiple neurological disorders (Chang et al., 2016; Young et al., 2011). Research into the process of neuronal migration and its regulatory mechanisms has evolved due to advances in molecular genetic technologies, allowing for the effects of individual genes on migration to be interrogated. This has relied on the development of assay platforms for neuronal migration, which have included organotypic slice cultures (Stoppini et al., 1991), transwell migration assays (Kramer et al., 2013), *ex vivo* explant assays (Leong et al., 2011), hanging drop assays (Falenta et al., 2013; Sonego et al., 2013), and 3D tumor spheroid assays (Vinci et al., 2015).

There is great potential in using phenotypic screening techniques to uncover mechanisms regulating neuroblast migration, but to date few successful studies have been published. Existing models for studying neuroblast migration have not been suitable to use in a high-throughput screen (HTS) due to the cost, labour-intensive design, or lack of reproducibility. There is considerable momentum in the drug discovery field to move from the reductionist 2D *in vitro* HTS platforms using immortalized cell lines to more physiologically relevant 3D models using primary cell types and 3D scaffolds for high-content screening. These models are far better mimics of complex tissue and adhere far better to a set of principles to facilitate the definition and development of disease-relevant assays (Hulkower and Herber, 2011). Building on established assay techniques, and taking these principles into consideration, we set out to develop a screening platform that was both physiologically relevant and highly reproducible.

Protein kinases have a vast array of roles in cellular activity, and have been strongly implicated in neuroblast migration. Several receptor tyrosine kinases are expressed by

This is an open access article under the CC BY license (http://creativecommons.org/licenses/by/4.0/).

neuroblasts and have been shown to modulate migration, including the IGF1R, Eph receptors EPHA4 and EPHB1–3, PDGFR, and VEGFR (Hurtado-Chong et al., 2009; Ishii et al., 2008; Kim et al., 2010; Mani et al., 2009; Todd et al., 2017). Some of these were surprising as they had been associated with regulating proliferation. The EGFR, or ERBB1, is necessary for driving proliferation and is a marker of mitotic progenitors in the SVZ, but it is also negatively correlated with neuroblast motility (Kim et al., 2009). A large collection of intracellular kinases mediate neuronal migration by phosphorylating key components of signaling pathways, such as CDK5, GSK3β, MAPK-upstream protein kinase, members of c-Jun N-terminal kinase family, and extracellular signal-regulated kinase (Kawauchi, 2014; Simo et al., 2007; Hirai et al., 2002; Konno et al., 2005). However, small-molecule compounds that stimulate neural progenitor migration *in vivo* is still an open challenge in drug discovery.

Our main aim was the development of a neuroblast migration assay that is more physiologically relevant and therefore a better tool to investigate large numbers of interventions to identify new drug targets and starting molecules for drug development. Compounds that increase migration of neuroblasts could have therapeutic potential in stroke, traumatic brain injury, or neurodegenerative disorders; and compounds that decrease migration may be useful as anti-invasive treatments for brain cancer. Therefore, a reliable assay to identify such compounds could be a powerful tool in neuroscience research. The Published Kinase Inhibitor Set (PKIS1 and PKIS2), an annotated set of approximately 900 small-molecule kinase inhibitors, was developed by GlaxoSmithKline due to the abundance of uncharacterized kinases (Drewry et al., 2014). The PKIS library has previously been used to investigate the functions of kinases in various cell types and diseases, including in neuroregeneration (Al-Ali et al., 2015). Using the PKIS, we validated the potential for our 3D spheroid migration assay to identify compounds that significantly increase or decrease neuroblast migration.

RESULTS

3D RMS Spheroid Migration Assay

Figure 1 shows the overall spheroid migration assay protocol. Neuroblast migration was studied by embedding the spheroids in Matrigel, which was previously reported to support SVZ cell migration *in vitro* (Azzarelli et al., 2017; Dizon et al., 2006) (Supplemental Results). Images of the spheroid we taken at 2, 24, and 48 h using an IN Cell Analyzer 6000 high-content image capture and analysis system (Figure 1). Figures S1–S3 show further details of the assay development.

Analysis of the frequency distribution in spheroid size indicated a Gaussian distribution, which was supported by a passed D'Agostino-Pearson omnibus normality test ($p = 0.1813$). The mean spheroid size was 27,437 ± 1,073 μm^2 confirming that the Greiner ULA plates used can produce large quantities of reproducible RMS spheroids. The area containing migrating neuroblasts (Figure 1E) only very rarely extended out of the imaged field, averting the need for upper thresholds. Moreover, excellent optical properties of our spheroid-containing wells make them compatible with various types of readout devices, such as standard and lens-free microscopes, high-content imaging systems, and microplate readers.

Application of Vital Dyes to 3D RMS Spheroids

Cell viability and general spheroid health were measured using propidium iodide and Hoechst (Figure 2). The Hoechst dye was imaged using the standard DAPI filter set (361 nm/497 nm) and propidium iodide can be imaged using the standard Texas Red filter set (535 nm/617 nm). Both of these dyes bind to DNA, but while Hoechst is cell permeable, PI cannot cross the membrane of live cells. Therefore, the combination is useful to differentiate apoptotic and healthy cells in a culture. CellTracker Green was also shown to effectively label migrating spheroid cells (Figure S4).

Image Acquisition

Uniform spheroids were established reproducibly and were robust enough for time-lapse microscopy. Imaging was primarily completed using the GE IN Cell Analyzer 6000, high-content imager (GE Healthcare). This system comprises both a bright-field and a line-scanning laser confocal microscope making it ideal for quick 3D cell imaging, in a high-content image format. A protocol was established on the IN Cell acquisition software that imaged the center of each well of the Greiner ULA plate. The starting plane was set by the microscope's built-in "software autofocus" function and nine optical sections were imaged in the z plane at intervals of 25 μm, which amounted to a total depth of 200 μm. Before starting the imaging, a trial scan was completed to check the position of the spheroid in each image. If any spheroid was dramatically off center or shifted in the z plane, the acquisition field was manually adjusted for that well. All scans included a bright-field image, taken above the well using an orange bright-field diode and a dsRed emission filter at an exposure of 0.2 s. When fluorescence was used, the exposure was set based on the individual signal level of that experiment. A single scan of the entire plate took 15 min on bright field and 25 min when using fluorescence channels. Images were taken every 30 min for 24 h (Figure 3).

Imaging was also carried out on three other systems, demonstrating versatility: (1) a Motic AE2000 inverted

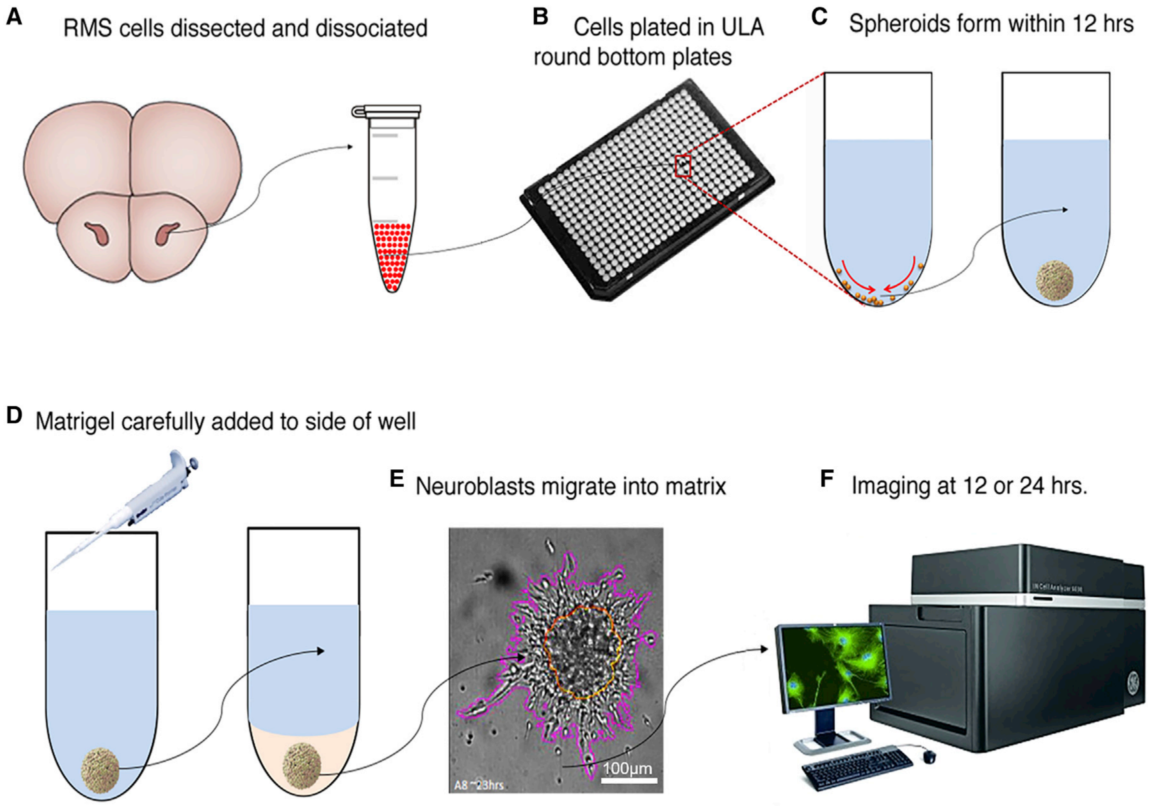

Figure 1. Schematic of Assay Developed for Neuroblast Migration

(A) The RMS (dark pink) is dissected from slices and single-cell dissociated.

(B and C) (B) Cells are plated in round bottom plates and (C) cells sink to bottom and form spheroids.

(D and E) (D) Matrigel is added to the spheroids and (E) neuroblasts emigrate from them in 3D. The image shows the spheroid (encircled in yellow) at 24 h with the emigrated cells manually outlined in purple to determine emigration surface area. Scale bar, 100 μm.

(F) Migration is imaged and measured at various time points.

microscope with Moticam 580 digital camera, (2) an Essen Bioscience Incucyte, and (3) an EVOS FL Auto. For the Essen Bioscience Incucyte, the 10× objective was used, imaging in the center of the plate with the software's built-in autofocus.

Manual Image Analysis

To manually quantify migration of cells from spheroids, individual bright-field image stacks were loaded into ImageJ and a maximum intensity projection created (Figure 4). Unlike explants and hanging drop aggregates, we found that cell migration from spheroids is relatively evenly distributed and continued for up to 24 h after plating (Figure S5). This property was exploited to provide a quick and simple quantification method. An ellipse was drawn around the spheroid such that it satisfied the following criteria: (1) it was centered on the spheroid, (2) it intersected at least six cells deemed to have migrated from the spheroid, and (3) it was round (circularity >0.8). The area was then calculated and saved as a "region of interest." In some instances, the "Find Edges" function was applied to assist in visualizing individual cells. When there were a few single cells that had migrated individually from the spheroid these were ignored so as not to skew the analysis (Figure 4). Any wells that were empty or in which the number of spheroids was greater than 1 were excluded. If the spheroid became misshapen or there was debris in the wells, they were excluded from analysis. Using these criteria, we excluded 4.2% of the wells.

Manual analysis of fluorescent nucleofected spheroids was completed by analysis of the average of the eight maximally migrated cells. The fluorescent channel image stacks were loaded into ImageJ and a maximum intensity projection created. The line function was then used to measure the distance from the spheroid edge to the ten cells that appeared furthest away making sure to save each as a region of interest. Only the top 8 of these measures were used for quantification.

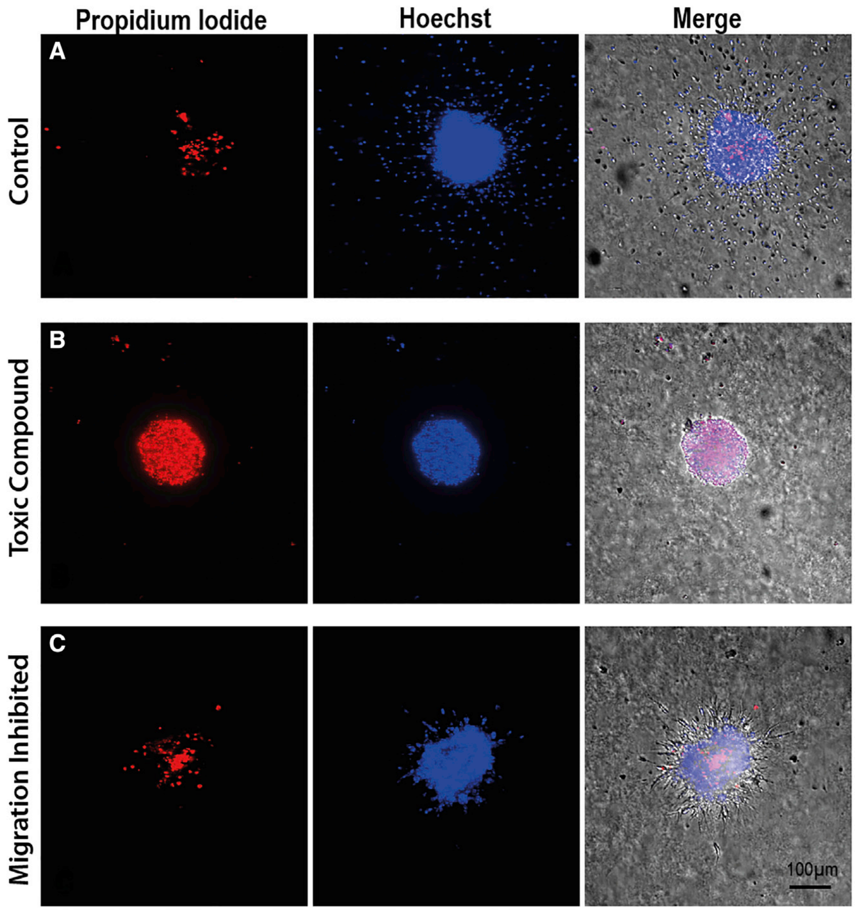

Figure 2. Spheroids Labeled with Fluorescent Dyes

(A) Control neurosphere shows few propidium iodide+ (PI+) cells (dead, red) but many Hoechst+ cells (live, blue) in the core and that have emigrated.

(B) A toxic compound (GW406731X) causes robust PI staining. Most cells died before they could emigrate.

(C) A migration inhibitor (GSK1389063A) allows robust survival but little emigration compared with the control spheroid. The right-hand column shows merge of two fluorescent channels and bright field. Scale bar, 100 μm.

Automated Image Analysis

Automated image analysis was carried out using a custom protocol developed in Developer Toolbox 1.9.2, GE IN Cell Analyzer 6000, high-content imager (Figure 5). To analyze collective cell migration from the spheroid, extended focus images were created from each bright-field image stack. A macro was written to apply the following functions to each image: Smooth > Find Edges > Dilation > Fill Holes > Erosion > Segment > Sieve. This resulted in segmentation of the spheroid and halo of migrated cells ready for quantification. To analyze the number and location of individual cells, fluorescence channels (Hoechst/propidium iodide) were used. First, extended focus projections were created for each image stack and the contrast enhanced. A macro was written to apply the following functions to each image: Segment > Fill Holes > Sieve. The Hoechst and propidium iodide images were then compared with identify live cells. With the central spheroid, halo, and all dead and alive cells segmented it is now possible extract features and quantify the migration. The area of the migration halo was calculated by subtracting the area of the spheroid core from the entire contiguous spheroid area. The halo area is a good measure of the extent of collective and chain migration from the spheroid. Similar to manual image analysis, two main exclusion criteria were used. The first was to omit wells where zero, two, or more spheroids were found and the second was to remove wells within which the spheroids were artificially misshaped due the incorporation of a fiber or other artifact. The upper adjacent value of the spheroid weighted moment of inertia, a measure of roundness, was used to identify misshapen spheroids. We also excluded cases of incorrect bright-field segmentation of the spheroid itself. Using these criteria a total of 8.5% of wells were excluded.

Validation of the 3D Spheroid Assay through a Kinase Inhibitor Screen of SVZ Neuroblast Migration

PKIS1 and PKIS2 were openly available in screening quantities from the Structural Genomics Consortium, University of Oxford. Both libraries were requested at www.sgc-unc.org and chemical structures and pharmacological activity profiles for the compounds found at https://www.ebi.ac.uk/chembldb/extra/PKIS/compounds.html.

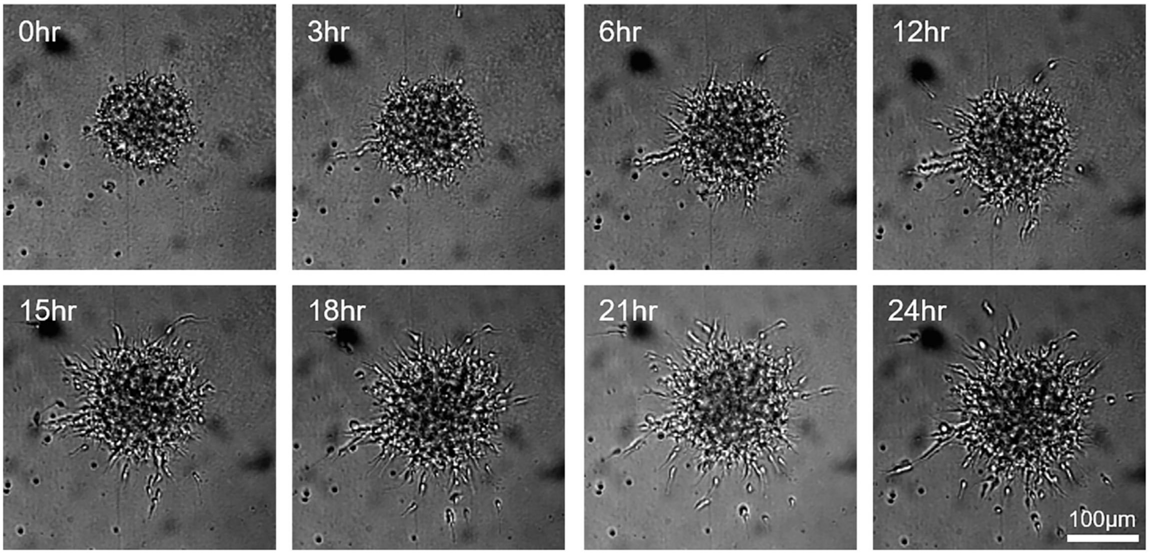

Figure 3. Time-Lapse Imaging of Spheroid Migration Using the GE IN Cell

The IN Cell imaging platform took images over a 24-h period from plating of a spheroid in Matrigel. Migration of neuroblasts out of the spheroid could already be observed at 3 h and continued throughout the duration of the time lapse. Scale bar, 100 μm.

Six 96-well plates of 3D RMS spheroids, with each well containing 4,000 cells, were treated with 10-μM compound or DMSO as a control and returned to the incubator for 2 h to incubate. Plates were then imaged at 2 h (T = 0) and 24 h with bright field only. Two hours before the 48-h endpoint, the live/dead assay was added to each well and the plates were assessed with automated imaging of bright field and fluorescence.

Triplicate wells for each compound were normalized against the DMSO controls, and cutoffs for "high migration" or "low migration" compounds introduced, as those producing a migration area at least 4 standard deviations greater or lesser, respectively, from the mean of the controls. For the low-migration compounds, toxicity was excluded with the live/dead assay. This experiment yielded 24 compounds that elicited high migration areas in the screen ranging from 1.7× to 3.0× that of controls, and 36 compounds that decreased migration, with areas ranging from 0.17× to 0.35× that of controls (Tables S1–S3). In total, 181 compounds were deemed toxic based on qualitative evaluation of cell death compared with controls.

The 60 compounds selected as putative modulators of migration were then triaged into a dose-response confirmation experiment. Each compound was tested in triplicate at concentrations up to 10 μM, and dose-response curves were made to identify the concentration that produces the greatest migration phenotype (Tables S4 and S5). The intended targets for which the compounds were designed are also listed in Tables S4 and S5. Table S4 shows the 21 compounds out of the 24 tested that significantly increased migration at the maximum 10 μM concentration. Representative images of spheroid migration and the compound structures are indicated. The profile of the dose-response curves varied across the hit compounds with some causing significant increases in migration at the lower concentrations (GW607049C, GW459135A, GSK2336394A, GW579362A, and GSK1024304A), while others increased migration only at the highest concentration (GW693881A and GW854278X). The initial screen was performed blind: the primary kinase targets of the small-molecule inhibitor hits were unknown at the time of treatments and active compounds were selected solely based on migration area. Table S5 shows the compounds that significantly reduced migration area in comparison with DMSO. All 36 compounds tested significantly reduced neuroblast migration at the highest concentration of 10 μM.

The lists of putative hits selected from each analysis method were then compared Figure 6. Please see Supplemental Results for a detailed description of criteria for hit selection in Figure 6. Several receptor tyrosine kinase targets were selected as inducing robust migration when inhibited. These included EGFR, ERBB2, ERBB4, EPHA2, TRKA, TRKC, PDGFR, TIE2, FLT3, and KIT. Other kinases selected included four members of the Polo-like kinase (PLK) family: PLK1, PLK2, PLK3, and LOK; NimA-related protein kinase 9; hormonally upregulated Neu-associated kinase; muscle-specific kinase (MuSK); and Aurora C kinase. Tyrosine kinase targets that reduced migration when inhibited included GSK3α, GSK3β, MAPK14/P38α,

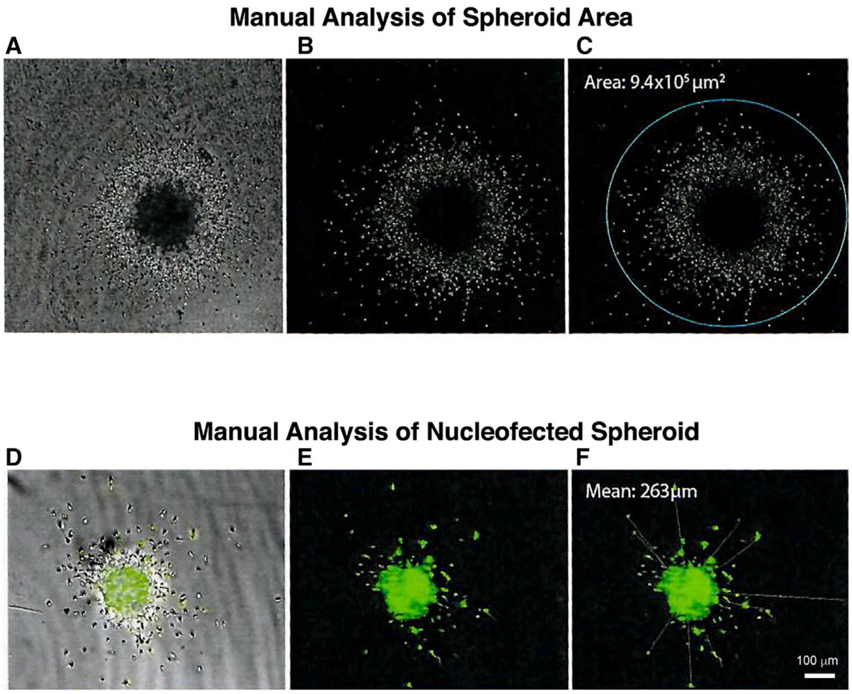

Figure 4. Images Showing the Workflow for Manually Quantifying Spheroid Images

(A) Brightfield image of spheroid and emigrated cells.

(B) A maximum intensity projection was created.

(C) The area of an elipse encircling the migration was calculated. The "Find Edges" function was used as needed to improve the signal-to-noise ratio.

(D) Overlap of brightfield and flouresence imaging of nucloefected cells.

(E) Fluorescence imaging of nucleofected cells.

(F) For manual analysis of nucleofected fluorescent cells, the average distance of the eight maximally migrated cells was measured. Scale bar, 100 microns.

IGF1R, IR, CDK2, protein kinase D1, casein kinase 1, and dual-specificity Yak 1-related kinase 1B. Details of the active compound's binding ratios and relative kinase affinities are provided in Table S1. Figure S6 shows STRING representations of kinase targets that increased and decreased migration in the PKIS screens.

Confirmation Studies Inhibiting MuSK and PIK3CB Promote the Migration of Neuroblast

We selected two less-well-studied kinases that significantly increased neuroblast migration in the spheroid screen when inhibited. The kinase MuSK was selected based on analysis of the affinity matrices and phosphatidylinositol-4,5-bisphosphate 3-kinase catalytic subunit beta isoform (PIK3CB) was selected because it was the only indicated binding partner of GSK2336394A, a compound validated to increase migration. Knockdowns (KD) for each kinase were generated with short hairpin RNAs (shRNAs) and verified by qPCR (Figure S7E). Migration of neuroblasts expressing plasmids for *pGIPZ* (GFP controls), *MuSK* KD and *PIK3CB* KD were measured. KD of both *MuSK* and *PIK3CB* significantly increased the average distance cells migrated compared with control cells (Figures 7 and S7) Figure S7, as predicted from our results. Interrogation of the Allen Brain Atlas also shows that *PIK3CB*, and *MuSK* and its ligand *LRP4*, are specifically highly expressed in the ventricular region (Figure 7C), further supporting their role in neurogenesis.

DISCUSSION

The results presented here detail the establishment of an *in vitro* phenotypic assay to study the migration of SVZ neuroblasts. Whereas several *in vitro* migration assays have been developed, to our knowledge none have been coupled to drug-screening approaches. An evolution of the hanging drop assay, 3D spheroids are generated from cells dissected from the mouse RMS in a robust and standardized way. The assay has been designed to be easy to implement and cost-effective using ULA round-bottomed plates. The spheroid assay is compatible with the vital dyes: CellTracker, propidium iodide, and Hoechst, as well as with cell nucleofection of KD constructs. It could therefore be used for this physiologically relevant higher-throughput assay and also to study mechanisms regulating migration and potentially other cellular behaviors. Whereas several *in vitro* migration assays have been developed, to our knowledge none have been coupled to drug-screening approaches. These results demonstrate the utility of the 3D spheroid migration assay to assess small-molecule regulation of SVZ neuroblast migration. Twenty-one compounds significantly increased migration of neuroblasts and 36 compounds reduced their migration. Confirmation studies of two kinase pathways were performed amongst previously uncharacterized kinases that may have regulatory roles in neuroblast migration.

The biggest source of variation was from the initial RMS dissection. In addition to varying the starting cell

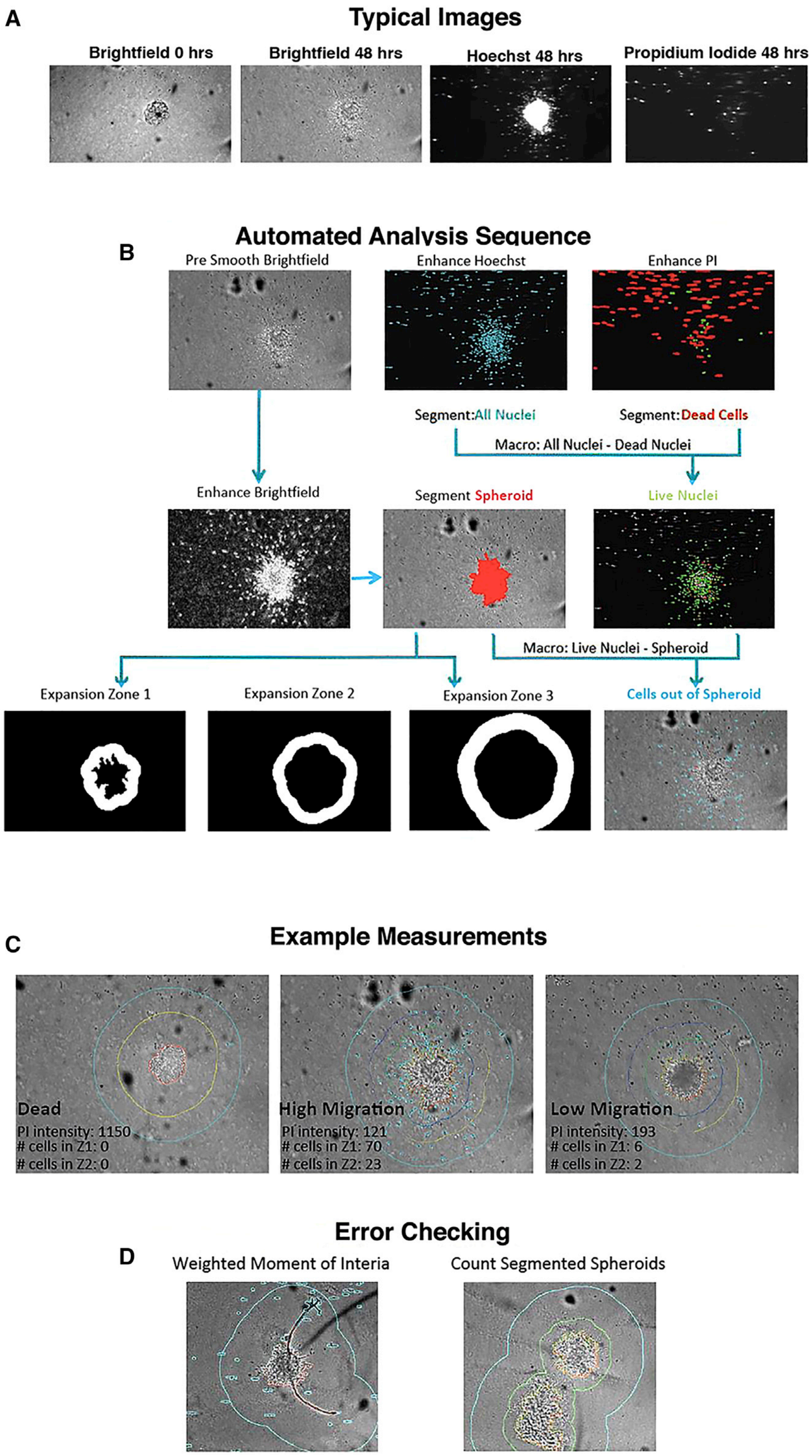

Figure 5. Workflow of Automated Spheroid Image Analysis

(A) Examples of typical bright-field, Hoechst (live stain) and propidium iodide (dead stain) images used in our analysis.

(B) The automated analysis sequence describes the imaging sequence and the processing carried out by the analysis software.

(C) Neuroblast numbers within increasing radius zones from the spheroid were measured by the software to determine migration extent.

(D) Errors affecting the counts were checked automatically.

population, imperfect dissection occasionally resulted in increased cell death, and thus smaller, less healthy spheroids with increased debris. One potential improvement could be to use transgenic mice, such as the DCX-GFP mouse where the RMS is labeled with a fluorescent protein (Nam et al., 2007). In fact, the early development of this

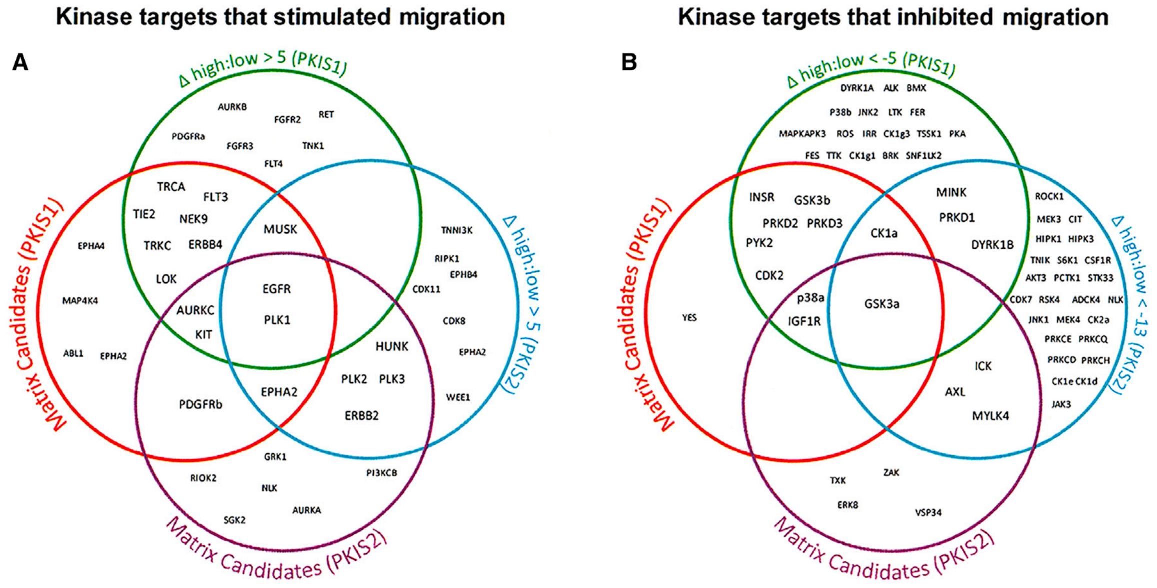

Figure 6. Comparison of Kinase Targets Selected by Analysis of the PKIS1 and PKIS2 Datasets

Venn diagrams of the putative kinase targets selected by the analysis of the kinase targets for the "hit" compounds and the relative kinase affinity to compounds increasing (A) or decreasing (B) migration for both PKIS1 and PKIS2.

assay was completed with one such mouse strain, the GAD65-GFP mouse (De Marchis et al., 2004; Nam et al., 2007), which made the dissection process far easier due to much more accurate visualization of the SVZ while also reducing preparation time, reducing cellular debris, and making imaging and quantification much easier. The spheroid migration assay has been developed to study neuroblasts. However, there is no reason why it cannot be applied to study different migratory cell types. For example, the SVZ also gives rise to oligodendrocyte precursor cells and visualisation of migration of these cells could be accomplished with OLIG2-GFP mice.

Automated image analysis has many advantages. It can substantially cut down analysis time and permit quantification that is technically difficult and/or laborious to do manually. It can also eliminate the inherent variability of manual scoring because computer measurements are not subject to external factors, such as human fatigue, illumination, or ambient noise. However, care must be taken at all steps of a high-content screen to avoid inconsistent imaging, artifacts, or contamination from foreign debris as this will severely compromise the image analysis results (Buchser et al., 2004). The automated analysis toolset developed in this article can quantify multiple parameters and can be optimized to quantify more if needed. It is important to consider that migration is a multistep process. The initiation, maintenance and termination of motility, speed, directionality, and percent of cells migrating can all affect overall migration. Limiting the time series to one cell cycle can to some extent control for proliferation but the results from any screen will need to be further investigated to understand more precisely what underlies the phenotype.

It is important to keep in mind the complexity of the *in vivo* environment dictating neuroblast migration compared with the reductionist approach of *in vitro* assays. *In vivo*, cell migration is modulated by a complex cellular and extracellular matrix architecture and a combination of short- as well as long-range chemoattractant and chemorepellent molecules. It is impossible to perfectly replicate, *in vitro*, the complex network of cells, structures, and signaling molecules that cells interact with *in vivo*. Indeed, although the neuroblasts radiating from the spheroids maintain the ability to migrate in a similar way to their *in vivo* counterparts, displaying chain migration and saltatory dynamics, they lack interactions with other RMS components, such as vasculature. It is widely accepted, in addition, that cells growing in a monolayer on plastic dishes have little in common with the complex 3D multicellular organization found in living organisms (Duval et al., 2017; Haycock, 2011). For this reason, it is believed that their form of migration is not equivalent to the chain migration typically observed *in vivo*. Our 3D spheroid assay is likely to more accurately reflect *in vivo* conditions then do monolayers. Explants are small (<1 mm^2) chunks of SVZ or RMS tissue that are embedded in an extracellular matrix, such as Matrigel. Neuroblasts contained within the explant are able to migrate out radially in 3D allowing the

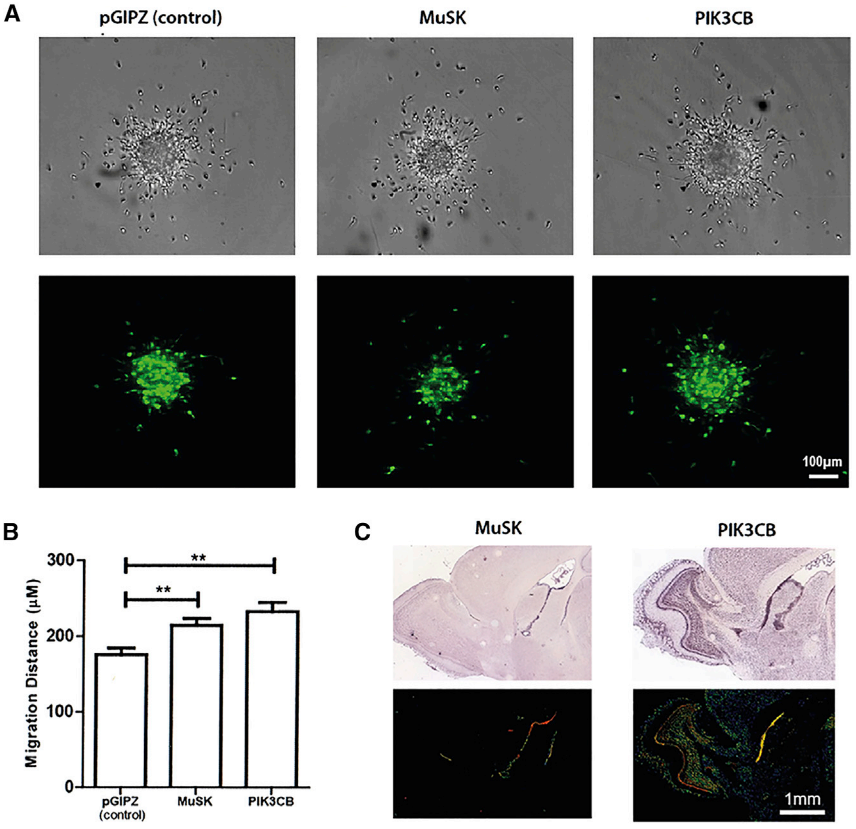

Figure 7. Knockdown of *MuSK* and *PIK3CB* Increased Migration of Neuroblasts from 3D Spheroids

(A) Representative images of RMS spheroids nucleofected with shRNA plasmids targeting *MuSK* and *PIK3CB*. Scale bar, 100 μm

(B) Quantification of the distance migrated by nucleofected neuroblasts shows that knockdown of both *MuSK* and *PIK3CB* increased the average migration distance.

(C) Images showing the expression of *MuSK*, *LRP4*, and *PIK3CB* in sagittal mouse brain sections (taken from Allen Brain Atlas). Scale bar, 1 mm.

measurement of multiple parameters, such as speed and directionality (Comte et al., 2011). Explants retain the complex cellular architecture of the brain and recapitulate both single-cell and chain migration (Comte et al., 2007). Leong et al. (2011) utilized explants to demonstrate that the ROCK1 inhibitor Y27632 disrupted chain formation. However, every explant is inherently different, leading to an unavoidably high sample-to-sample variation and they are thus not suitable for HTS. Another limitation of explants is that because the tissue remains intact, genetic manipulations are almost impossible without the use of previous *in vivo* work. Two separate studies used an embryonic organotypic brain slice culture, which identified the Src family kinase inhibitor PP2, the PKC inhibitors BIM1 and Ro318220, and 11 novel nonannotated compounds as disruptors of cortical radial migration (Jossin et al., 2003; Zhou et al., 2007). *In vitro* slice assays are even more naturalistic and have been successfully used to study RMS migration (James et al., 2011; Kim et al., 2009; Nam et al., 2007); however, they are impractical for HTS formats as only a few sagittal slices containing the RMS can be generated per animal.

The development of this 3D spheroid neuroblast migration assay is a useful tool for identifying pathways and small molecules that mediate neuroblast migration. The higher-throughput assay with the PKIS library validated the utility of the assay, and two hits, MuSK and PIK3CB, were further confirmed by KD of these genes. These data can be used in the future to develop therapeutic strategies for inducing emigration of RMS neuroblasts for injury or reducing infiltration in cancer from the RMS. Use of the PKIS library for neuroblast migration is a good example of re-purposing libraries, it was previously used to screen for compounds that could impact necroptosis (Hildebrand et al., 2014) and the sleeping sickness protozoan (Amata et al., 2016). More similar to our goals, PKIS was used in a high-content screen to find targets that could promote neurite outgrowth in primary neuronal cultures as a model for axon regrowth after injury to the brain (Al-Ali et al., 2015). Kinases that promoted or repressed neurite outgrowth were identified. A top hit for increasing neurite outgrowth, which showed polypharmacology, was tested *in vivo* in a mouse model of spinal cord injury and the compound promoted growth of corticospinal axons (Al-Ali et al., 2015). This study demonstrates the importance of developing physiologically relevant screens for neuroregeneration so that promising compounds *in vitro* can be reliably translated to *in vivo* trials (Al-Ali et al., 2015).

Kinases are key mediators of cell migration and invasion processes in brain cancer, via regulation of mesenchymal genes and rearrangement of cytoskeletal components. Cyclin-dependent kinase 5 (CDK5) modulates neuroblast migration through phosphorylation of cytoskeletal proteins. CDK5 deletion is associated with impairment in chain formation, speed, directionality, and extension of leading processes of neuroblasts (Hirota et al., 2007; Kawauchi, 2014). The cell-polarity factors glycogen synthase kinase-3β and protein kinase Cζ are needed for centrosome reorientation (Higginbotham et al., 2006). Inhibition of the PI3KAkt pathways is known to disrupt neuroblast migration and regulates neuroblast migration in stroke (Katakowski et al., 2003; Meng et al., 2010). Despite the roles of a number of protein kinases in migration being well studied, the mechanisms by which each protein kinase is involved in neuronal migration remains far from complete. Therapeutic strategies targeting kinases has been a major focus of drugs for tumorigenesis and metastasis and our assay can be incorporated into this important effort. The phenotypic assay also revealed compounds that blocked migration. These data can be used in the future to provide potential therapeutic strategies for cancer or in research into the mechanisms of neuroblast migration in the RMS. The kinase targets of the compounds that inhibited migration are yet to be further validated experimentally. Nevertheless, initial analysis of the affinity matrices provides confidence in the assay by identifying pathways known to be critical to neuroblast migration and also indicates the role of some lesser-studied kinases (Supplemental Discussion).

The large diversity in chemical structures of hit compounds suggests that neuroblast migration may be promoted through modulating a variety of target kinases and corresponding host networks. Additive effects may therefore be achieved by co-treating spheroids with hits that act via distinct biological targets. Applying machine-learning algorithms may also help to illuminate the most relevant biological targets and systems. One potential option would be to use Support Vector Machines. These are computer-based learning algorithms that recognize patterns given examples belonging to each of two categories and build a model that can classify new examples into either category. Thus, they can be used to predict kinase inhibition profiles that will have maximal biological effect.

In conclusion, we have developed an *in vitro* neuroblast migration assay coupled to a bespoke screening approach, the combination of which offers several distinct advantages. It uses primary cells rather than transformed cell lines and thus it likely better represents neuroblast *in vivo* behavior. The spheroid 3D organization was chosen as it is more naturalistic than monolayer cultures. Our proof-of-principle work shows broad applicability of the technique and also that it is relatively fast, easy, and cheap and thus accessible to most laboratories interested in mechanism discovery. Finally, automation of cell imaging and quantification renders our approach suitable for pharmacological screens of small-molecule modulators.

EXPERIMENTAL PROCEDURES

Culturing 3D RMS Spheroids

P3-P5 CD1 wild-type mice pups were culled and their brains removed and sliced into 300-μm sections on a microtome. To dissect out the RMS tissue, the OB and the anterior-most two to three sections containing the RMS were collected in ice-cold Hanks' balanced salt solution (HBSS) (Gibco 10888-022) (Figure S1). Under a dissection microscope and using 27G syringe needles the RMS was carefully dissected and transferred into a 2-mL Eppendorf tube on ice. The RMS was identified as the translucent, region at the center of the OB sections which are cell dense and thus appear dark in Nissl stains (Figure S1). As the RMS is followed back into the more posterior sections, it elongates in a C shape. This was also collected until the ventricle begins to open up. We did not collect RMS tissue from the lateral ventricle region as it contains ciliated ependymal cells, which we noticed could prevent correct spheroid formation. The experiments were performed in accordance with the UK Animals (Scientific Procedures) 1986 Act, UK Home Office. All animal work was approved by the UK Home Office, License #30/2496, and the University of Oxford Department of Physiology, Anatomy and Genetics Departmental Ethical Review Committee.

The dissected RMS tissue was spun down briefly using a bench top centrifuge and the HBSS carefully removed. The tissue was then resuspended in 2 mL Accutase (Thermo Fisher A1110501) and carefully dissociated by gently triturating with a P200 Pipetman. It was very important that all cells were dissociated from one another. The Accutase was diluted out by adding 2 mL of the cells to 8 mL of NB-A+ medium (Neurobasal-A) (Gibco 10888-022) supplemented with 1% Glutamax (Gibco 35050-038) and 2% B27 (Gibco 17504). The cell suspension was transferred through a 40-μm cell strainer (Corning 352340) into a 50-mL Falcon tube and washed with a further 10-mL of NB-A+. The cell suspension was spun down at 300 × *g* for 5 min, resuspended in 1 mL of NB-A+ medium and counted using a hemocytometer. Typically, we collected approximately 500,000 cells per mouse. The cells were diluted to a concentration of 50,000 cells per ml of NB-A+ and 5,000 cells were added to each well of a 96-well ultra-low adhesion round bottom plate (Greiner 650970). The culture plate was then sealed with a gas-permeable seal (4TI 044850) and incubated overnight at 37°C, 5% CO_2, during which spheroids formed. It was critical to not spin the plate down as this trapped cell debris in the center of the spheroid.

The spheroid plate was removed from the incubator and left to cool to room temperature. Matrigel was diluted to 50% in ice-cold NB-A+ medium. The spheroid plate was then briefly chilled in ice and the seal removed. Matrigel (25 μL) was then carefully trickled down the side of each well using a multichannel pipette

and the plate returned to ice for the Matrigel to settle to the bottom of the well. The plate was then re-sealed and left for 3 min to warm to room temperature before replacing in the incubator for the Matrigel to set.

Preparation of shRNA Plasmid DNA

Plasmid DNA was prepared as per the EndoFree Maxi Kit (QIAGEN, 12,362). In brief, stock cultures from the Dharmacon mouse shRNA library (cat. no. RMM5829) were cultured overnight at 37°C in 3 mL Luria-Bertani (LB) broth supplemented with ampicillin. The following day the cell suspension was plated overnight on LB agarose supplemented with ampicillin. The next day, three single colonies were picked per plate and transferred to 3 mL of LB broth supplemented with ampicillin for 4 h before being transferred to 250 mL of LB broth supplemented with ampicillin and left to shake at 37°C overnight. The following morning the cultures were processed as per the QIAGEN kit protocol apart from the following change to the final stage. Once the DNA pellet had been precipitated out using isopropanol, it was redissolved in 500 μL pure water and transferred to a 2-mL Eppendorf tube. Ethanol (1,400 μL, 100%) was added, mixed well, and spun down at maximum speed for 5 min on a desk top microcentrifuge. The ethanol was carefully removed and the DNA pellet allowed to dry completely. The DNA pellet was resuspended in 50 μL pure water and the concentration measured using a NanoDrop. Table S6 shows the shRNA constructs used for kinase KDs. Table S7 shows the primers used to verify the KDs.

Reverse Transcription

Complimentary DNA (cDNA) was synthesized using SuperScript III Reverse Transcriptase. One microliter of 250 ng/μL random hexamers (N8080127) and 1 μL of 10 mM dNTP mix (Thermo Fisher R0191) was added to 1 μg of DNase-treated RNA, and made up to 13 μL with nuclease-free H20 (Sigma-Aldrich, Gillingham, UK). The mix was incubated at 65°C for 5 min to denature RNA secondary structure and then incubated on ice for 1 min. The mix was made up to 20 μL with 4 μL 5× first strand buffer (Life Technologies), 5 mM dithiothreitol (18080-044), 40 units RNaseOUT (10777), 1 μL (200 units) SuperScript III Reverse Transcriptase (Invitrogen, 18080-044) and 3 μL of nuclease-free H_2O. Samples were incubated in a PCR machine on an RT protocol: 25°C for 5 min, 55°C for 1 h (optimal temperature for Superscript III), and 70°C for 15 min (termination of the reaction). Samples were diluted in 100 μL nuclease-free H_2O for qPCR.

Primer Design

PCR primer pairs were designed to amplify an amplicon of approximately 100 base pairs. DNA sequences for genes of interest were obtained from the University of California, Santa Cruz, genome browser (http://genome.ucsc.edu) and primers designed using Integrated DNA Technology (IDT) software (http://www.idtdna.com/primerquest/home/index). GC content was kept between 40% and 60%, and primers were designed with an annealing temperature between 55°C and 65°C with only 1°C between the pair. They were designed to have minimal self-complementarity and no complementarity to the other primer and were tested for single-peak melt curves using uMelt software (https://www.dna.utah.edu/umelt/umelt.html). RT-PCR primers were designed to span large exon boundaries to prevent the amplification of any residual genomic DNA. The GAPDH primer pair used to test for genomic DNA contamination did not span exon boundaries. Oligonucleotides were synthesized by IDT. They were resuspended in nuclease-free H_2O to a stock concentration of 100 μM and used at a working concentration of 2 μM.

RT-PCR

The reaction consisted of 33 ng template nucleic acid (4 μL), 10 μL 2× SYBR Green PCR Master Mix, 0.2 μM primer mix (3 μL), and nuclease-free H_2O to make a total reaction volume of 20 μL. All PCR reactions were carried out with one negative control for each primer pair, where H_2O was used as the template. RT-PCR reactions were carried out on a Chromo4 Real-Time PCR detector (Bio-Rad, Bath, UK) using the following parameters: initial denaturation 95°C for 15 min, denaturation 95°C for 30 s, annealing 60°C for 30 s, extension 72°C for 30 s × 45 cycles. Fluorescence was recorded at the end of each cycle. A melt curve was carried out from 60°C to 95°C with a 10-s hold and a plate read at every 1°C increment. Each reaction was carried out with a technical replicate and with three biological replicates. Relative transcript quantification used the cycle threshold (CT) values for the ΔΔCT method to determine relative expression of the target gene to confirm KD. The expression of the gene of interest was calculated by normalizing against the housekeeper gene, and two-tailed, independent t tests were performed in Prism 6 (GraphPad) to test for level of KD of the gene, with significance determined as *$p < 0.05$, **$p < 0.01$, ***$p < 0.001$, ****$p < 0.0001$.

Postnatal Electroporation

In vivo SVZ electroporation was carried as in previous work (Al-Dalahmah et al., 2020; Sun et al., 2018). In brief, plasmids used for postnatal electroporation were prepared with EndoFree Plasmid Maxi Kits as above. Before use, 1% Fast Green in PBS (0.22 μm filtered) was mixed with the plasmid solution at 1:10 for visualizing injection. P0–P1 pups were deeply anesthetized by hypothermia on wet ice. Plasmid solution (~2 μL, 5 μg/μL) was injected into the right lateral ventricle via a pulled glass capillary. Very brief, electrical current was passed across the snouts (−100 V, 50 ms ON with 850-ms intervals for 5 cycles) (BTX ECM 830) by tweezer electrodes coated with conductive gel. After surgery, pups were kept in a 37°C warming box for recovery and then returned to their cage. The pups were monitored for the following 4 days to check for adverse reactions to the procedure and on the 5th day the pups were culled and the electroporated hemispheres removed for spheroid preparation.

Nucleofection of Primary RMS Cells

Cells from the RMS were nucleofected via a modified version of Lonza protocol VPG-1001. In brief, the RMS was dissected out as described in the main text, dissociated, counted, and split across 2 to 4 different 15-mL Falcon tubes containing 1×10^6 cells and 5 mL NB-A+ medium. The cells were spun down at 300 × g for 5 min, the supernatant removed and then very carefully

resuspended in 50% of the standard nucleofection solution diluted in HBSS minus calcium and magnesium (41 μL of Nucleofector Solution + 9 μL of supplement + 50 μL of HBSS) and 5 μg DNA before being transferred into the certified cuvette. Nucleofector program G-13 was used instead of the recommended O-005. Pre-warmed culture medium (500 μL) was added into the cuvette and the sample was then transferred by the supplied pipette into 1 mL medium and centrifuged at 300 × *g* for 5 min. Cells were then suspended with 4 mL fresh medium, plated in a single well per condition in a 6-well plate and left to recover at 37°C. After 4 h the cells were collected and used as normal for the remainder of the spheroid assay.

PKIS High-Content Imaging

We used the IN Cell Analyzer 6000 high-content imager for image acquisition. This system comprises both bright field and a line-scanning laser confocal microscope making it ideal for quick 3D cell imaging, albeit at a slightly poorer resolution than standalone point-scanning confocal microscopes. A protocol was established on the IN Cell acquisition software that imaged the center of each well of the Greiner ULA plate. The starting plane was set by the microscope's in-built "software autofind" function and nine Z slices were imaged at 25 μm per well, which amounted to a total section of 200 μm. Before starting the imaging experiment a trial scan was completed to check the position of the spheroid in each image. Should any spheroid be dramatically off center or shifted in the Z plane the acquisition field was manually set for that well. All images were taken with the 10× 0.45NA objective. The IN Cell has four lasers (UV, blue, green, and red) and four capture filters (DAPI, GFP, dsRed, and Cy5). Images were taken above the well using an orange bright-field diode and dsRed emission filter at an exposure of 0.2 s. When fluorescence was used the exposure was set based on the individual signal level of that experiment. To scan each plate, it took around 15 min on bright field only and around 25 min when using fluorescence.

SUPPLEMENTAL INFORMATION

Supplemental Information can be found online at https://doi.org/10.1016/j.stemcr.2020.07.012.

AUTHOR CONTRIBUTIONS

M.D., D.E., and F.S. carried out experimental design. M.D. and V.M. carried out the experiments. M.D., V.M., and F.S. carried out data analysis. M.D., V.M., D.E., and F.S. wrote the paper.

ACKNOWLEDGMENTS

The kinase inhibitor set PKIS and PKIS2 were provided the SGC-UNC and include kinase inhibitors donated by GlaxoSmithKline, Pfizer, and Takeda. Funding for the SGC-UNC was provided by The Eshelman Institute for Innovation, UNC Lineberger Comprehensive Cancer Center, PharmAlliance and NIH (1R44TR001916-02, 1R01CA218442-01, and U24DK116204-01). The SGC is a registered charity (no. 1097737) that receives funds from AbbVie, Bayer Pharma AG, Boehringer Ingelheim, Canada Foundation for Innovation, Eshelman Institute for Innovation, Genome Canada, Innovative Medicines Initiative (EU/EFPIA) (ULTRA-DD grant no. 115766), Janssen, Merck KGaA Darmstadt Germany, MSD, Novartis Pharma AG, Ontario Ministry of Economic Development and Innovation, Pfizer, Takeda, and Wellcome (106169/ZZ14/Z).

Received: January 15, 2020
Revised: July 10, 2020
Accepted: July 11, 2020
Published: August 6, 2020

REFERENCES

Al-Ali, H., Lee, D.H., Danzi, M.C., Nassif, H., Gautam, P., Wennerberg, K., Zuercher, B., Drewry, D.H., Lee, J.K., Lemmon, V.P., et al. (2015). Rational polypharmacology: systematically identifying and engaging multiple drug targets to promote axon growth. ACS Chem. Biol. *10*, 1939–1951.

Al-Dalahmah, O., Campos Soares, L., Nicholson, J., Draijer, S., Mundim, M., Lu, V.M., Sun, B., Tyler, T., Adorjan, I., O'Neill, E., et al. (2020). Galectin-3 modulates postnatal subventricular zone gliogenesis. Glia *68*, 435–450.

Alvarez-Buylla, A., and Garcia-Verdugo, J.M. (2002). Neurogenesis in adult subventricular zone. J. Neurosci. *22*, 629–634.

Amata, E., Xi, H., Colmenarejo, G., Gonzalez-Diaz, R., Cordon-Obras, C., Berlanga, M., Manzano, P., Erath, J., Roncal, N.E., Lee, P.J., et al. (2016). Identification of "preferred" human kinase inhibitors for sleeping sickness lead discovery. Are some kinases better than others for inhibitor repurposing? ACS Infect. Dis. *2*, 180–186.

Azzarelli, R., Oleari, R., Lettieri, A., Andre, V., and Cariboni, A. (2017). In vitro, ex vivo and in vivo techniques to study neuronal migration in the developing cerebral cortex. Brain Sci. *7*. https://doi.org/10.3390/brainsci7050048.

Buchser, W., Collins, M., Garyantes, T., Guha, R., Haney, S., Lemmon, V., Li, Z., and Trask, O.J. (2004). Assay development guidelines for image-based high content screening, high content analysis and high content imaging. In Assay Guidance Manual, G.S. Sittampalam, A. Grossman, K. Brimacombe, M. Arkin, D. Auld, C.P. Austin, J. Baell, B. Bejcek, J.M.M. Caaveiro, and T.D.Y. Chung, et al., eds. (Eli Lilly & Company and the National Center for Advancing Translational Sciences). https://www.ncbi.nlm.nih.gov/books/NBK100913/pdf/Bookshelf_NBK100913.pdf.

Chang, E.H., Adorjan, I., Mundim, M.V., Sun, B., Dizon, M.L., and Szele, F.G. (2016). Traumatic brain injury activation of the adult subventricular zone neurogenic niche. Front Neurosci. *10*, 332.

Comte, I., Tran, P.B., and Szele, F.G. (2007). Techniques and strategies to analyze neural progenitor cell migration. Curr. Pharm. Biotechnol. *8*, 177–185.

Comte, I., Kim, Y., Young, C.C., van der Harg, J.M., Hockberger, P., Bolam, P.J., Poirier, F., and Szele, F.G. (2011). Galectin-3 maintains cell motility from the subventricular zone to the olfactory bulb. J. Cell Sci. *124*, 2438–2447.

De Marchis, S., Temoney, S., Erdelyi, F., Bovetti, S., Bovolin, P., Szabo, G., and Puche, A.C. (2004). GABAergic phenotypic differentiation of a subpopulation of subventricular derived migrating progenitors. Eur. J. Neurosci. *20*, 1307–1317.

Dizon, M.L., Shin, L., Sundholm-Peters, N.L., Kang, E., and Szele, F.G. (2006). Subventricular zone cells remain stable in vitro after brain injury. Neuroscience *142*, 717–725.

Drewry, D.H., Willson, T.M., and Zuercher, W.J. (2014). Seeding collaborations to advance kinase science with the GSK published kinase inhibitor set (PKIS). Curr. Top Med. Chem. *14*, 340–342.

Duval, K., Grover, H., Han, L.H., Mou, Y., Pegoraro, A.F., Fredberg, J., and Chen, Z. (2017). Modeling physiological events in 2D vs. 3D cell culture. Physiology (Bethesda) *32*, 266–277.

Falenta, K., Gajendra, S., Sonego, M., Doherty, P., and Lalli, G. (2013). Nucleofection of rodent neuroblasts to study neuroblast migration in vitro. J. Vis. Exp., e50989. https://doi.org/10.3791/50989.

Haycock, J.W. (2011). 3D cell culture: a review of current approaches and techniques. Methods Mol. Biol. *695*, 1–15.

Higginbotham, H., Tanaka, T., Brinkman, B.C., and Gleeson, J.G. (2006). GSK3beta and PKCzeta function in centrosome localization and process stabilization during Slit-mediated neuronal repolarization. Mol. Cell. Neurosci. *32*, 118–132.

Hildebrand, J.M., Tanzer, M.C., Lucet, I.S., Young, S.N., Spall, S.K., Sharma, P., Pierotti, C., Garnier, J.M., Dobson, R.C., Webb, A.I., et al. (2014). Activation of the pseudokinase MLKL unleashes the four-helix bundle domain to induce membrane localization and necroptotic cell death. Proc. Natl. Acad. Sci. U S A *111*, 15072–15077.

Hirai, S., Kawaguchi, A., Hirasawa, R., Baba, M., Ohnishi, T., and Ohno, S. (2002). MAPK-upstream protein kinase (MUK) regulates the radial migration of immature neurons in telencephalon of mouse embryo. Development *129*, 4483–4495.

Hirota, Y., Ohshima, T., Kaneko, N., Ikeda, M., Iwasato, T., Kulkarni, A.B., Mikoshiba, K., Okano, H., and Sawamoto, K. (2007). Cyclin-dependent kinase 5 is required for control of neuroblast migration in the postnatal subventricular zone. J. Neurosci. *27*, 12829–12838.

Hulkower, K.I., and Herber, R.L. (2011). Cell migration and invasion assays as tools for drug discovery. Pharmaceutics *3*, 107–124.

Hurtado-Chong, A., Yusta-Boyo, M.J., Vergano-Vera, E., Bulfone, A., de Pablo, F., and Vicario-Abejon, C. (2009). IGF-I promotes neuronal migration and positioning in the olfactory bulb and the exit of neuroblasts from the subventricular zone. Eur. J. Neurosci. *30*, 742–755.

Ishii, Y., Matsumoto, Y., Watanabe, R., Elmi, M., Fujimori, T., Nissen, J., Cao, Y., Nabeshima, Y., Sasahara, M., and Funa, K. (2008). Characterization of neuroprogenitor cells expressing the PDGF beta-receptor within the subventricular zone of postnatal mice. Mol. Cell. Neurosci. *37*, 507–518.

James, R., Kim, Y., Hockberger, P.E., and Szele, F.G. (2011). Subventricular zone cell migration: lessons from quantitative two-photon microscopy. Front Neurosci. *5*, 30.

Jossin, Y., Bar, I., Ignatova, N., Tissir, F., De Rouvroit, C.L., and Goffinet, A.M. (2003). The reelin signaling pathway: some recent developments. Cereb. Cortex *13*, 627–633.

Katakowski, M., Zhang, Z.G., Chen, J., Zhang, R., Wang, Y., Jiang, H., Zhang, L., Robin, A., Li, Y., and Chopp, M. (2003). Phosphoinositide 3-kinase promotes adult subventricular neuroblast migration after stroke. J. Neurosci. Res. *74*, 494–501.

Kawauchi, T. (2014). Cdk5 regulates multiple cellular events in neural development, function and disease. Dev. Growth Differ. *56*, 335–348.

Kim, Y., Comte, I., Szabo, G., Hockberger, P., and Szele, F.G. (2009). Adult mouse subventricular zone stem and progenitor cells are sessile and epidermal growth factor receptor negatively regulates neuroblast migration. PLoS One *4*, e8122.

Kim, Y., Wang, W.Z., Comte, I., Pastrana, E., Tran, P.B., Brown, J., Miller, R.J., Doetsch, F., Molnar, Z., and Szele, F.G. (2010). Dopamine stimulation of postnatal murine subventricular zone neurogenesis via the D3 receptor. J. Neurochem. *114*, 750–760.

Koizumi, H., Higginbotham, H., Poon, T., Tanaka, T., Brinkman, B.C., and Gleeson, J.G. (2006). Doublecortin maintains bipolar shape and nuclear translocation during migration in the adult forebrain. Nat. Neurosci. *9*, 779–786.

Konno, D., Yoshimura, S., Hori, K., Maruoka, H., and Sobue, K. (2005). Involvement of the phosphatidylinositol 3-kinase/rac1 and cdc42 pathways in radial migration of cortical neurons. J. Biol. Chem. *280*, 5082–5088.

Kramer, N., Walzl, A., Unger, C., Rosner, M., Krupitza, G., Hengstschlager, M., and Dolznig, H. (2013). In vitro cell migration and invasion assays. Mutat. Res. *752*, 10–24.

Leong, S.Y., Faux, C.H., Turbic, A., Dixon, K.J., and Turnley, A.M. (2011). The rho kinase pathway regulates mouse adult neural precursor cell migration. Stem Cells *29*, 332–343.

Mani, N., Khaibullina, A., Krum, J.M., and Rosenstein, J.M. (2009). Vascular endothelial growth factor enhances migration of astroglial cells in subventricular zone neurosphere cultures. J. Neurosci. Res. *88*, 248–257.

Meng, X., Arocena, M., Penninger, J., Gage, F.H., Zhao, M., and Song, B. (2010). PI3K mediated electrotaxis of embryonic and adult neural progenitor cells in the presence of growth factors. Exp. Neurol. *227*, 210–217.

Merkle, F.T., Mirzadeh, Z., and Alvarez-Buylla, A. (2007). Mosaic organization of neural stem cells in the adult brain. Science *317*, 381–384.

Ming, G.L., and Song, H. (2005). Adult neurogenesis in the mammalian central nervous system. Annu. Rev. Neurosci. *28*, 223–250.

Nam, S.C., Kim, Y., Dryanovski, D., Walker, A., Goings, G., Woolfrey, K., Kang, S.S., Chu, C., Chenn, A., Erdelyi, F., et al. (2007). Dynamic features of postnatal subventricular zone cell motility: a two-photon time-lapse study. J. Comp. Neurol. *505*, 190–208.

Ng, K.L., Li, J.D., Cheng, M.Y., Leslie, F.M., Lee, A.G., and Zhou, Q.Y. (2005). Dependence of olfactory bulb neurogenesis on prokineticin 2 signaling. Science *308*, 1923–1927.

Simo, S., Pujadas, L., Segura, M.F., La Torre, A., Del Rio, J.A., Urena, J.M., Comella, J.X., and Soriano, E. (2007). Reelin induces the detachment of postnatal subventricular zone cells and the expression of the Egr-1 through Erk1/2 activation. Cereb. Cortex *17*, 294–303.

Sonego, M., Gajendra, S., Parsons, M., Ma, Y., Hobbs, C., Zentar, M.P., Williams, G., Machesky, L.M., Doherty, P., and Lalli, G.

(2013). Fascin regulates the migration of subventricular zone-derived neuroblasts in the postnatal brain. J. Neurosci. *33*, 12171–12185.

Stoppini, L., Buchs, P.A., and Muller, D. (1991). A simple method for organotypic cultures of nervous tissue. J. Neurosci. Methods *37*, 173–182.

Sun, B., Chang, E., Gerhartl, A., and Szele, F.G. (2018). Polycomb protein Eed is required for neurogenesis and cortical injury activation in the subventricular zone. Cereb. Cortex *28*, 1369–1382.

Todd, K.L., Baker, K.L., Eastman, M.B., Kolling, F.W., Trausch, A.G., Nelson, C.E., and Conover, J.C. (2017). EphA4 regulates neuroblast and astrocyte organization in a neurogenic niche. J. Neurosci. *37*, 3331–3341.

Vinci, M., Box, C., and Eccles, S.A. (2015). Three-dimensional (3D) tumor spheroid invasion assay. J. Vis. Exp., e52686. https://doi.org/10.3791/52686.

Young, C.C., Brooks, K.J., Buchan, A.M., and Szele, F.G. (2011). Cellular and molecular determinants of stroke-induced changes in subventricular zone cell migration. Antioxid. Redox Signal. *14*, 1877–1888.

Zhou, L., Jossin, Y., and Goffinet, A.M. (2007). Identification of small molecules that interfere with radial neuronal migration and early cortical plate development. Cereb. Cortex *17*, 211–220.

Stem Cell Reports, Volume *15*

Supplemental Information

A Semi-automated and Scalable 3D Spheroid Assay to Study Neuroblast Migration

Martin Ducker, Valerie Millar, Daniel Ebner, and Francis G. Szele

Supplemental Methods

The following shRNA plasmids were selected from the Dharmacon GIPZ Lentiviral Mouse shRNA Library (RMM5829).

Table S6. shRNA constructs used for kinase gene knockdown

Reference	Gene Target	Vector	Sense Sequence
Abl1#1	*Abl1*	pGIPZ	CTCTTATAAATGACATGTA
Abl1#2	*Abl1*	pGIPZ	GAACCACCATTCTACATAA
Hunk#1	*Hunk*	pGIPZ	CAAAGAGATCTTTCTTTAT
Mertk#1	*Mertk*	pGIPZ	CTGTTATATTCCCGATTAA
Mertk#2	*Mertk*	pGIPZ	CGGAACATAAAAATGTCAA
MuSK#1	*MuSK*	pGIPZ	GGAGCTTAAGGATCCATAA
Nek9#2	*Nek9*	pGIPZ	AGATGTTTACCCAACTACA
Pik3cb#1	*Pik3cb*	pGIPZ	CTCACATCGGTCAAAGATA
Pik3cb#2	*Pik3cb*	pGIPZ	CTGGAGAACTTGGAAGATA

Table S7 Table of primers used for confirmation of shRNA knockdown

Gene	Forward Sequence	Reverse Sequence	Source
Plk1	CTTCGCCAAATGCTTCGAGAT	TAGGCTGCGGTGAATTGAGAT	Life Technologies
Riok2	TAAGCTGTTCAACAATCCCTCC	GCTGCTTGGTAAACACATTGG	Life Technologies
Abl1	CAGCAGCCTGGAAAAGTTCTT	CCCTGCCCCTTTGATAAAATGC	Life Technologies
Hunk	GTGCTGACGGGAGAAAAGGTA	GGGTGTCGGATCATCTGCTG	Life Technologies
Mertk	CCTAACCGTACCTGGTCTGAC	GGGAGGGGATTACTTTGATGTTG	Life Technologies
MuSK	TACAGAGGGGAGGTGTGTGAT	TCCCGGTAGGAGGTGTTGAA	Life Technologies
Nek9	TACGAGCGACACTGCGATTC	ACGCGGATGGGGATGTAGT	Life Technologies
Pik3cb	CTATGGCAGACAACCTTGACAT	CTTCCCGAGGTACTTCCAACT	Life Technologies

DNAse treatment of RNA To remove residual genomic DNA contamination, all RNA samples were treated with TURBO DNA- freeTM (Invitrogen AM1907). 2µg of extracted RNA was combined with 1µl of TURBO DNase, 2.5µl of TURBO DNase Buffer and made up

to 25µl with nuclease-free $H_2 0$. The sample was incubated for 50 min at 37°C. The DNase was subsequently inactivated by incubation with 6µl of DNase Inactivation Reagent at RT for 5 min, vortexing every 30 seconds. The sample was then centrifuged at 13,000 RPM for 90 seconds and the supernatant was transferred to a new Eppendorf tube and stored at -80°C.

Supplemental Results

The RMS was dissected (Figure S1) and spheroids generated as described in the Supplemental Results. The assay was initially trialled with GAD65-GFP mice (Figure S2A). Neuroblasts migrating out from the spheroids are able to self-organise into chains closely recapitulating *in vivo* migration (Figure S2B), a critical feature in a physiologically relevant migration assay. Spheroids ranging from 600 to 10,000 cells were generated and all resulted in robust migration (Figure S3A). In subsequent work we systematically eliminated factors confounding image analysis (Figure S3B).

The use of GAD65-GFP or similar transgenic mice is recommended for anyone new to RMS dissections to improve dissection accuracy, consistency and speed. The RMS is more translucent and distinguishable from surrounding tissue and most researchers are able to make this distinction with short training times. We estimate that 75-85% of cells in our samples are neuroblasts. Because we generate a stock of single cells, mix them and aliquoted into each well there is little variation in the extent of migration from control neurospheres.

GAD65-GFP cells form spheroids

To confirm that the Greiner ULA plate system could be applied to RMS cells, we set up a pilot experiment in which the RMS was dissected from GAD65-GFP mice (Figure S2A). This is a convenient mouse strain for this work because the majority of neuroblasts in the RMS express GFP making dissecting and subsequent imaging relatively straightforward. As well, we have extensively studied GAD65-GFP neuroblast migration in slices with 2-photon microscopy (Nam et al., 2007). Two P4 mice pups were used, which provided sufficient cells for culturing 5,000 cells in each of the middle 20 wells of a 96 well plate. The cells naturally reaggregated overnight and formed well-defined and uniform spheroids. In the pilot experiment one well had to be excluded due to debris, nevertheless 19 uniform spheroids with an average 2D surface area of 15,603±214 μm^2 (Figure S2A). Thus, spheroid are expected to be normally distributed with variances in initial sizes expected to arise only due to statistical fluctuation.

Neuroblast migration from 3D RMS spheroids recapitulates *in vivo* migration and is distinct from neurosphere migration

A critical feature in developing an SVZ neuroblast migration assay is that it recapitulates in vivo features such as chain migration that occurs *in vivo*. It is argued that chain migration is orchestrated by SVZ glial tubes, however, as Figure S2B illustrates, neuroblasts radiating out from the spheroids were able to self-organise into chains. As evidenced by the GAD65-GFP expression in Figure S2A, RMS spheroids were predominantly composed of

neuroblasts. It was observed that neuroblasts routinely changed directions while migrating and alternated between pauses and bursts of fast migration, which is consistent with previously described behaviors of neuroblasts migrating in slices (James et al., 2011). Almost all cells emerged from the spheroid in chains and then continued to migrate as individual cells, often following the path of previous neuroblasts. As a means of rapid quantification of images, a relatively simple segmentation of the radiation area was quantified using the InCell analysis Software (Methods) (Figure S5). Thresholding enabled the segmentation of both the central spheroid and the halo of cells that has migrated out of the spheroid in chains. The number of single cells could easily be counted for either the entire image or within a given area. To quantify distance of migration it is possible to expand the area of the spheroid by set distances and quantify the number of cells that occupy that area. Repeating this several times creates defined zones that can provide data on the frequency distribution of the distance migrated by the neuroblasts. The spacing of these bins can be set depending on the requirements of the experiment. However, it is worth considering how to plan to compare across the sample set. The ratio of the area of the halo to the number or area occupied by single cells may also be used as an indicator of conditions that modulate collective or chain migration. Although single neuroblasts could be discerned at the end of the experiment, it was not possible to capture time-lapse single cell migration using this method, however, single cell tracking may be an option for future time-lapse experiments.

It is important to emphasize here that the 3D RMS spheroids are distinct from SVZ neurospheres used in the field for ascertaining SVZ stem cell properties, such as self-renewal, proliferation and fate choice. Neurospheres have a heterogeneous cell composition that varies depending on age, seeding density, culture conditions, and passage number. Nevertheless, the predominant cell type is generally considered to be the rapidly dividing transit amplifying cells (Gil-Perotin et al., 2013). Dcx+ neuroblasts are only found in very few numbers during the self-renewal phase of neurosphere cultures. This concurs with the consistent finding from our group that the radial migration from neurospheres is almost entirely of glial cells. Moreover, glial migration is amoebic in nature and does not display the mixture of chain and single cell migration evident in both the explant assay and now the spheroid assay.

Assay Optimisation

Once it had been determined that RMS spheroid formation was robust it was important to optimise a few criteria. The first of these was spheroid size or cell number per well. The greater the cell number the more continuous the radiating cell halo becomes, making image quantification more consistent. Moreover, a greater number of cells permits greater complexity in 3D cellular architecture. However, the transfer of nutrients into the spheroid centre gradually decreases with spheroid size, potentially causing variations in cell viability or metabolism. In SVZ neurospheres this is known to lead to the formation of hollow necrotic cores once the diameter exceeds ~150µm (Ge et al., 2012). In addition, monitoring cell migration with videomicroscopy is limited by the field size and camera aperture, thus smaller spheroids may enable migration to be tracked over longer periods of time. A balance is therefore required to retain physiological relevance, while permitting high content screening.

Figure S3A shows spheroids plated at cell densities between 600 and 40,000 cells per well. Imaging at 24 hours shows that the spheroids with over 10,000 cells at the start of the assay contain a lot of migrating cells, but the cells with highest levels of migration do not remain within the field of image, and therefore cannot be reliably measured. The largest spheroids also develop necrotic centres due to the lack of nutrients reaching the spheroid core. However, the spheroids tested with less than 2,500 cells do not have sufficiently large numbers of cells to migrate from the spheroid, and therefore do not make a reliable high throughput assay. The best compromise under the experimental conditions and equipment available were spheroids of 5,000 cells.

Matrigel is a soluble basement membrane biomaterial extracted from Engelbreth-Holm-Swarm mouse tumours; it contains several molecules including laminin, collagen IV, heparan sulfate proteoglycans, and entactin/nidogen-1, 2. It is liquid below 4°C but sets to a gel above 10°C. We used Growth Factor Reduced Matrigel (BD 356231). Importantly, Matrigel is viscous; it has a protein concentration of 8-12mg/ml and will sink in water. Matrigel™ is relatively expensive, therefore we next sought to determine whether the volume of Matrigel could be reduced. The concentration of Matrigel used is known to strongly influence neuroblast migration so we were reluctant to vary this dramatically (Ward and Rao, 2005). However, experience from explant assays indicates that neuroblasts grow well *in vitro* in drop volumes down to ~10µl. When pipetted carefully with a single tip pipette we confirmed that the volume of Matrigel used could be reduced to 20µl. However, when attempted with a multichannel pipette this volume proved too restrictive as roughly 10% of the spheres failed to embed correctly. The smaller volume of Matrigel displaced the spheroid from the centre of the well, instead of enveloping it, resulting in the spheroids being displaced to the side (Figure S3B). While this may not necessarily effect invasion it hindered both imaging and analysis as steep sides of the U bottom create image aberrations (Figure S3B). We hypothesised that this was due to the leading edge of the Matrigel beginning to polymerise as it reaches the bottom of the well. To overcome this, the culture plate with the spheroids was cooled on ice prior to embedding and the Matrigel volume was increased to 25µl. This resolved the problem of spheroid centring but introduced a second complication. Using ice resulted in watermarks on the bottom of the plate that introduced large artefacts into the brightfield image background (Figure S3B). These proved very difficult to prevent via simple cleaning measures. Wiping with alcohol wipes was either too mild to remove the mark or too harsh; either creating scratches on the plate surface or dislodging the Matrigel plug. To cool the spheroid plate, we therefore used a large reusable icepack recycled from a consumable delivery covered in a single sheet of tissue paper.

Another strategy considered was miniaturisation to a 384 well format. The smaller volumes and tighter gradient of curvature of the U-bottomed 384 well plates (Corning) allowed the volume of Matrigel per well to be reduced to just 10µl. However, because of the increased well curvature, large spherical aberrations were created making them unsuitable for migration assays that require a large image field (Figure S3B). This, in addition to the increased plate cost and technical difficulty, confirmed the 96 well Greiner ULA plate with 25µl 50% Matrigel as the best format.

The other most pertinent issues to overcome in the experimental set up are represented in Figure S3B. The media composition needed to be optimised to sustain dense spheroid formations while minimising potential variants. Spheroids formed in standard neurobasal

media supplemented with B27 "complete media". There was no need for additional supplementation with serum or growth factors. However, culture medium with low insulin (5nM) did not support spheroid formation. Debris in the cultures was important to minimise as this disrupted spheroid formation and integrity. Debris came in two forms 1) contamination (large debris), and 2) Cellular debris. This assay will test the experimenter's aseptic technique. Any contamination that falls in the well will be focused into the centre and incorporated in the spheroid. Arm sleeves are highly recommended to prevent fibre contamination from lab coats. Cellular debris were cells that had not reached the centre of the well and were not incorporated in the spheroid. They were not necessarily dead and could retain the ability to migrate. This did not create a huge obstacle to manual image analysis where the experimenter can be more discerning but for automation it could be a problem. The amount of debris is largely dependent on the accuracy and speed of the dissection. Very little debris is experienced when using a fluorescently labelled mouse line, such as the GAD65-GFP or DCX-GFP strains. However, when using wild type tissue, a degree of contamination was unavoidable (Figure S3B). In some instances where the SVZ was accidently dissected, ciliated ependymal cells could also disrupt spheroid formation entirely. The best strategy to overcome this is to improve dissection technique. If dissecting more than four brains in one sitting it is also advisable to add in a recovery period after the dissection to let the cells recover and limit the addition of dead or dying cells to the ULA plate. Even with these measures it was impossible to form a spheroid with absolutely no debris. For very high-quality images, it is advised to pick the spheroids out of the well and wash prior to embedding.

Live Dead Assay

The combination of Hoechst 33342 (ThermoFisher 62249) and Propidium iodide (PI) (Invitrogen P3566) was used to assess the viability/cytotoxicity in the spheroid assay. A concentrated stock of Live/Dead reagent was prepared by adding 5µg/ml Propidium Iodide solution and 10µg/ml Hoechst to NB-A+. Two hours prior to the final timepoint of the assay the plates were removed from the incubator, the seal carefully removed and 10µl of Live/Dead reagent added to each well. The plate was returned to the incubator for the remaining time period and assayed as normal. Both dyes worked well in RMS spheroids, however the compact spheroid mass created dye gradients necessitating staining time to be increased from 10 minutes to 60 minutes to reach equilibrium. The response of the spheroid bodies and the migrating cells to screened compounds can be used to distinguish between compounds that inhibit migration vs those that are cytotoxic (Figure 4). However, due to the cytotoxicity of the vital dyes themselves this assay should only be used as an endpoint.

CellTracker Green

CellTracker Green is a more stable form of the thiol-reactive Fluorescent Probe Calcein-AM that is suitable for long-term cell labelling (Kim et al., 2009). It passes freely through cell membranes, but once inside the cell, is transformed into fluorescent cell-impermeant reaction product. To avoid uneven dye transfer into the compact spheroid mass, the dye was added to the dissected cells prior to spheroid formation. CellTracker stained all cells in the spheroid with no obvious detriment to spheroid formation or neuroblast migration (Figure S4). Single cells could easily be identified, thus providing the option to use high content analysis software to count and measure the dynamics of single neuroblasts.

Published Kinase Inhibitor Set (PKIS) migration assay

The PKIS1 is a collection of 367 compounds that are split across 5x96 well plates and the PKIS2 is a collection of 645 compounds that are split across 7x96 well plates. We used 4,000 cells per well rather than 5,000 to increase the number of compounds that could be included. We split the experiment into 4 separate rounds where each round screened 3 plates in duplicate. Six 96 well plates of 3D RMS spheroids were prepared overnight as described above. Each spheroid contained 4,000 cells and was suspended in 80µl of Neurobasal. The compound libraries were diluted to a 5X (50µM) working concentration in Neurobasal. Columns 1 and 2 were used as DMSO controls. 20µl of working concentration compound was added to each well of the spheroid plates and they were returned to the incubator for 2 hrs to incubate. The drugs were added manually with a StarLab ErgoOne multichannel pipettor. To embed the spheroids in Matrigel the culture plates were cooled on an icepack for ~5 minutes and then 25µl of ice cold 50% Matrigel was carefully added down the side of the well. The plates were then sealed with a gas permeable, optically clear seal (4tittude) and returned to the incubator to set for 2hrs. Plates were then imaged at 2 hours (T=0) and 24 hours with brightfield microscopy. 2hrs prior to the 48hr endpoint the Live/Dead assay was added to each well and the plates were imaged on brightfield, UV and 488. Figures S8 and S9 show compounds, migration images and concentration response curves for kinase inhibitors that increased and decreased migration, respectively.

Analysis of the binding targets of the small molecules indicates potential genes that regulate neuroblast migration.

The PKIS is comprised of inhibitors that function via competitive binding with the cofactor ATP. Because the ATP binding pocket is conserved but not identical across the kinome, many of the PKIS compounds have some degree of promiscuity in their kinase activity. Consequently, the kinase for which a compound was originally prepared might not be the only kinase inhibited, and importantly the original kinase may not be a contributor to the phenotype resulting from compound treatment. Both the PKIS1 and PKIS2 have been characterised to try to provide insights into their likely molecular targets. The PKIS1 was analysed using the Nanosyn enzyme assay panel to determine the binding affinity to 224 recombinant human kinases (Elkins et al., 2016) and the PKIS was analysed using the KINOMEscan competition binding assay against 468 kinases. The results for both provide a percentage of kinase bound the inhibitor compared to DMSO (Drewry et al., 2017). It is important to note that neither panel is exhaustive so will not identify all potential interactions and that there are many kinases characterised for PKIS2 that are absent from the PKIS1 data set. It is also important to note that affinity is not necessarily correlated with potency.

We sought to select the most likely candidate kinase targets from the compounds screened for confirmation studies. The primary method used was to select candidate hit kinases from the affinity matrix for compounds that were validated to increase and decrease migration. To account for the promiscuous polypharmacology only hits in which there were 2 or fewer kinases with an affinity greater than 90 were analysed. The top two kinase hits were then selected for each compound as potential regulators of migration. The second approach we used was to analyse the distribution of binding affinities across the entire data set to identify kinase targets that are concentrated in the higher and lower migration groups. The rationale for this is that the results from the Nanosyn and KINOMEscan profiles do not necessarily

perfectly correlate with inhibition efficiency, so simply screening for hits greater than 90 will indiscriminately mask targets that have low affinity. Conversely, there may be kinases that have a consistently high affinity that are picked up in the screen, but do not actually have any functional activity. The cut offs used were if the migration area was >1.5x control, compounds were allocated to the high migrating pool and if it was <0.5x control, the compounds were allocated to the low migrating pool. All toxic compounds were removed from the analysis. The average affinity of each kinase to all compounds in the two categories were then calculated, compared and ranked. The kinases with a Δ high:low above 5 were considered as potential hits, where inhibition may increase or decrease migration for both PKIS1 and PKIS2 (Table S1). The kinases with a Δ high:low less than -5 were also considered as potential hits where inhibition decreased migration for PKIS1 (Table S1). For PKIS2 the number of putative hits was >200 using -5 as the threshold, and so it was adjusted to -13 (Table S1). The lists of putative hits selected from each analysis method were then compared.

STRING is an accumulated database of known and predicted protein-protein interactions, including both direct (physical) and indirect (functional) associations (Szklarczyk et al., 2017). It was a useful tool to utilise for this screen, to interrogate the kinases that are likely central to the molecular mechanisms behind migration. Proteins that were hits in the screen and have known associations with other proteins that were also hits could be important in future experiments that investigate the migration process. Figure S6A shows the STRING connections between the kinases that, when inhibited, increased migration. These kinases therefore may well normally inhibit migration in the brain. The stem cell growth factor receptor Kit may be an interesting kinase to investigate further as it links two different protein pathway hubs. PLK1, PLK2 and PLK3 were all hits for increasing migration and have known interactions with other proteins that produced the same phenotype and could have important roles in regulating migration. EGFR, the most common target of the compounds that increased migration, has many interactions with other proteins that significantly altered migration, including Kit.

Figure S6B shows kinases that decreased migration and the known interactions between these kinases from the accumulated STRING databases (Snel et al., 2000). MAPK14, an alias for P38A, which was the most common target of compounds that decreased migration is central to the STRING protein interaction map. It is closely associated with MAPK11, an alias for P38B, which was also a significant target for decreasing migration, and both proteins have many interactions in the network. The number of kinases that decreased migration that are closely associated with one another suggests that there may be a common mechanism in this process.

Optimising nucleofection for the spheroid migration assay

The ability to genetically manipulate cells of interest is useful in modern screening, and to demonstrate the wider application of the 3D RMS spheroid assay, we included a method to successfully nucleofect RMS cells prior to formation of 3D spheroids. Initially the Nucleofection programme O-005 was used as per the Lonza protocol (VPG-1001). However, this protocol did not yield compact, round spheroids (Figure S7A). Instead many small spheroids formed, which all collected at the bottom of the well. (A gelatinous substance in the well appeared to prevent complete unification.) We next used setting A-33, another

programme designed for nucleofection of mouse primary neural cells. After nucleofection, cells were healthy and when washed, dissociated and re-plated could re-aggregate and form spheroids. However, when these secondary spheroids were embedded the subsequent migration was limited (Figure S7B).

Another technique used to study neuroblast migration is *in vivo* electroporation. In this technique high concentration plasmid DNA is injected directly into the lateral ventricle of P0-P2 pups and then a pulse electric current is applied across the skull. This robustly labels SVZ stem cells with the plasmid and also therefore their progeny, the neuroblasts (Boutin et al., 2008). We therefore attempted to electroporate *in vivo* the pGIPZ plasmid 5 days prior to dissection. Using this method, spheroids formed correctly but the number of GFP+ cells contained within each spheroid was too low in these experiments to quantify (Figure S7C). Nevertheless most of our other in vivo electroporation experiments have resulted in many hundreds to thousands of labelled neuroblasts in the RMS and OB (Sun et al., 2018), and thus it is likely that this approach could be further optimised for Spheroid migration assays.

Therefore, two modifications were made to the protocol from the Lalli group (Falenta et al., 2013). Firstly, the Nucleofection Solution was diluted in Hanks Balanced Saline Solution minus calcium and magnesium. The rationale was that the lipophilic nature of the reagent may be disrupting membrane integrity. Secondly the nucleofection programme was changed to G-13, which has a reduced electric pulse power. With these modifications the nucleofection of dissociated neuroblasts did not disrupt spheroid formation and the efficiency was sufficiently high (~50%) to quantify migration (Figure S7D). This was confirmed with knockdown of *MuSK* and *Pik3cb* (Figure S7E-H).

Supplemental Discussion

Inhibition of phosphatidylinositol-4,5-bisphosphate 3-kinase catalytic subunit beta isoform (PIK3CB) was found to significantly increase neuroblast migration. PIK3CB is one of the four catalytic subunits of PI3K. Interestingly although in non-neuronal cells these catalytic subunits may have partially redundant functions, there is increasing evidence that in neurons their roles are more specialised, and confined to distinct receptor-dependent pathways. For instance, while the key mediator of IGF1R signalling is PIK3CA, PIK3CB is the major catalytic isoform downstream of the ERBB4 (Law et al., 2012). Activation of PIK3CB by ERBB4 is orchestrated via Yes-associated protein (YAP), the nuclear effector of Hippo signalling (Yan et al., 2017). It is therefore tempting to consider the opposing function of IGF1R and the ERBBs in neuroblast migration may involve differential activation of PIK3CA and PIK3CB. PIK3CB is also the predominant subunit associated with GPCRs (Guillermet-Guibert et al., 2008). The phosphatase and tensin homologue (PTEN), a negative regulator of PI3K activity, was shown to preferentially bind to PIK3CB compared to other PI3K catalytic subunits. PTEN deletion has been shown to disrupt neuroblast migration and, like PIK3CB, has been linked to autism (Gregorian et al., 2009; Zhu et al., 2012). However, the specific regulation of PIK3CB by PTEN in the SVZ remains unstudied.

Genetic validation with shRNA knockdown further supported a function of Muscle-specific tyrosine kinase receptor (MuSK) in neuroblast migration. MuSK is a receptor tyrosine kinase

that is mainly expressed in the muscle and plays an essential role in neuromuscular junction (NMJ) formation (Garcia-Osta et al., 2006). This function requires the interaction of MuSK with the heparan-sulfate proteoglycan Agrin and Low-density lipoprotein receptor-related protein 4 (LRP4). The role of MuSK in the central nervous system is poorly understood, however it has been reported to have a role in memory consolidation (Garcia-Osta et al., 2006; Sun et al., 2016). According to the Allen Brain Atlas MuSK, LRP4 and Agrin expression is evident in the SVZ and hippocampus and MuSK is concentrated at excitatory synapses, marked by coexpression of PSD-95 (Garcia-Osta et al., 2006; Ksiazek et al., 2007). Mice and zebrafish deficient in MuSK have defects in neural crest migration (Banerjee et al., 2011). In the absence of MuSK, streaming neural crest cells, which are normally confined to the central region of each somite, are dispersed and present throughout the somite. Specific knockdown of Agrin in the SVZ by postnatal electroporation disrupted integration, morphological differentiation and survival of new olfactory bulb interneurons (Burk et al., 2012).

The mitogen-activated protein (MAP) kinase family are well-documented regulators of migration in various cell types (Huang et al., 2004). Several are indicated by the screen as potential mediators of neuroblast migration, including MAPK8/Jnk1, MAPK9/Jnk2, MAPK14/p38a and MAPK11/p38b. Of these p38a can be found within the top hits selected by all methods used to interrogate the screen data set. The role of p38α in the SVZ remains incompletely understood but evidence supports a role in migration. It is highly expressed in the brain from E10 and is retained in the SVZ and choroid plexus of the adult brain (Sato et al., 2008). Pharmacological inhibition has been shown to decrease migration and a cell permeable p38 enhances migration *in vitro* without affecting cell survival or differentiation (Hamanoue et al., 2016). p38 also correlates with poor prognosis in several types of cancers, including glioblastoma (Goldsmith et al., 2018). p38 inhibitors have, therefore, attracted significant attention for use in chemotherapy, with at least 22 different compounds being investigated in Phase I/II. Yet despite their potential, high toxicity and off-target effects have severely limited their therapeutic value and none have reached Phase III. It is therefore of significant interest to identify the downstream effectors of p38. One possibility proposed by the screen data set in MAPK-activated protein kinase 3 (MAPKAPK3). This is a known substrate of p38 that is essential for migration of smooth muscle and endothelial cells (Hedges et al., 1999; Rousseau et al., 1997).

SI References

Banerjee, S., Gordon, L., Donn, T.M., Berti, C., Moens, C.B., Burden, S.J., and Granato, M. (2011). A novel role for MuSK and non-canonical Wnt signaling during segmental neural crest cell migration. Development *138*, 3287-3296.

Boutin, C., Diestel, S., Desoeuvre, A., Tiveron, M.C., and Cremer, H. (2008). Efficient in vivo electroporation of the postnatal rodent forebrain. PLoS ONE *3*, e1883.

Burk, K., Desoeuvre, A., Boutin, C., Smith, M.A., Kroger, S., Bosio, A., Tiveron, M.C., and Cremer, H. (2012). Agrin-signaling is necessary for the integration of newly generated neurons in the adult olfactory bulb. J Neurosci *32*, 3759-3764.

Drewry, D.H., Wells, C.I., Andrews, D.M., Angell, R., Al-Ali, H., Axtman, A.D., Capuzzi, S.J., Elkins, J.M., Ettmayer, P., Frederiksen, M.*, et al.* (2017). Progress towards a public chemogenomic set for protein kinases and a call for contributions. PLoS One *12*, e0181585.

Elkins, J.M., Fedele, V., Szklarz, M., Abdul Azeez, K.R., Salah, E., Mikolajczyk, J., Romanov, S., Sepetov, N., Huang, X.P., Roth, B.L.*, et al.* (2016). Comprehensive characterization of the Published Kinase Inhibitor Set. Nat Biotechnol *34*, 95-103.

Falenta, K., Gajendra, S., Sonego, M., Doherty, P., and Lalli, G. (2013). Nucleofection of Rodent Neuroblasts to Study Neuroblast Migration In vitro. J Vis Exp.

Garcia-Osta, A., Tsokas, P., Pollonini, G., Landau, E.M., Blitzer, R., and Alberini, C.M. (2006). MuSK expressed in the brain mediates cholinergic responses, synaptic plasticity, and memory formation. J Neurosci *26*, 7919-7932.

Ge, C., Yang, Q., Zhao, G., Yu, H., Kirkwood, K.L., and Franceschi, R.T. (2012). Interactions between extracellular signal-regulated kinase 1/2 and p38 MAP kinase pathways in the control of RUNX2 phosphorylation and transcriptional activity. J Bone Miner Res *27*, 538-551.

Gil-Perotin, S., Duran-Moreno, M., Cebrian-Silla, A., Ramirez, M., Garcia-Belda, P., and Garcia-Verdugo, J.M. (2013). Adult neural stem cells from the subventricular zone: a review of the neurosphere assay. Anat Rec (Hoboken) *296*, 1435-1452.

Goldsmith, C.S., Kim, S.M., Karunarathna, N., Neuendorff, N., Toussaint, L.G., Earnest, D.J., and Bell-Pedersen, D. (2018). Inhibition of p38 MAPK activity leads to cell type-specific effects on the molecular circadian clock and time-dependent reduction of glioma cell invasiveness. BMC Cancer *18*, 43.

Gregorian, C., Nakashima, J., Le Belle, J., Ohab, J., Kim, R., Liu, A., Smith, K.B., Groszer, M., Garcia, A.D., Sofroniew, M.V.*, et al.* (2009). Pten deletion in adult neural stem/progenitor cells enhances constitutive neurogenesis. J Neurosci *29*, 1874-1886.

Guillermet-Guibert, J., Bjorklof, K., Salpekar, A., Gonella, C., Ramadani, F., Bilancio, A., Meek, S., Smith, A.J., Okkenhaug, K., and Vanhaesebroeck, B. (2008). The p110beta isoform of phosphoinositide 3-kinase signals downstream of G protein-coupled receptors and is functionally redundant with p110gamma. Proc Natl Acad Sci U S A *105*, 8292-8297.

Hamanoue, M., Morioka, K., Ohsawa, I., Ohsawa, K., Kobayashi, M., Tsuburaya, K., Akasaka, Y., Mikami, T., Ogata, T., and Takamatsu, K. (2016). Cell-permeable p38 MAP kinase promotes migration of adult neural stem/progenitor cells. Scientific reports *6*, 24279.

Hedges, J.C., Dechert, M.A., Yamboliev, I.A., Martin, J.L., Hickey, E., Weber, L.A., and Gerthoffer, W.T. (1999). A role for p38(MAPK)/HSP27 pathway in smooth muscle cell migration. J Biol Chem *274*, 24211-24219.

Huang, C., Jacobson, K., and Schaller, M.D. (2004). MAP kinases and cell migration. J Cell Sci *117*, 4619-4628.

James, R., Kim, Y., Hockberger, P.E., and Szele, F.G. (2011). Subventricular zone cell migration: lessons from quantitative two-photon microscopy. Front Neurosci *5*, 30.

Kim, Y., Comte, I., Szabo, G., Hockberger, P., and Szele, F.G. (2009). Adult mouse subventricular zone stem and progenitor cells are sessile and epidermal growth factor receptor negatively regulates neuroblast migration. PLoS One *4*, e8122.

Ksiazek, I., Burkhardt, C., Lin, S., Seddik, R., Maj, M., Bezakova, G., Jucker, M., Arber, S., Caroni, P., Sanes, J.R.*, et al.* (2007). Synapse loss in cortex of agrin-deficient mice after genetic rescue of perinatal death. J Neurosci *27*, 7183-7195.

Law, A.J., Wang, Y., Sei, Y., O'Donnell, P., Piantadosi, P., Papaleo, F., Straub, R.E., Huang, W., Thomas, C.J., Vakkalanka, R.*, et al.* (2012). Neuregulin 1-ErbB4-PI3K signaling in schizophrenia and phosphoinositide 3-kinase-p110delta inhibition as a potential therapeutic strategy. Proc Natl Acad Sci U S A *109*, 12165-12170.

Nam, S.C., Kim, Y., Dryanovski, D., Walker, A., Goings, G., Woolfrey, K., Kang, S.S., Chu, C., Chenn, A., Erdelyi, F.*, et al.* (2007). Dynamic features of postnatal subventricular zone cell motility: A two-photon time-lapse study. J Comp Neurol *505*, 190-208.

Rousseau, S., Houle, F., Landry, J., and Huot, J. (1997). p38 MAP kinase activation by vascular endothelial growth factor mediates actin reorganization and cell migration in human endothelial cells. Oncogene *15*, 2169-2177.

Sato, K., Hamanoue, M., and Takamatsu, K. (2008). Inhibitors of p38 mitogen-activated protein kinase enhance proliferation of mouse neural stem cells. J Neurosci Res *86*, 2179-2189.

Snel, B., Lehmann, G., Bork, P., and Huynen, M.A. (2000). STRING: a web-server to retrieve and display the repeatedly occurring neighbourhood of a gene. Nucleic Acids Res *28*, 3442-3444.

Sun, B., Chang, E., Gerhartl, A., and Szele, F.G. (2018). Polycomb Protein Eed is Required for Neurogenesis and Cortical Injury Activation in the Subventricular Zone. Cereb Cortex *28*, 1369-1382.

Sun, X.D., Li, L., Liu, F., Huang, Z.H., Bean, J.C., Jiao, H.F., Barik, A., Kim, S.M., Wu, H., Shen, C.*, et al.* (2016). Lrp4 in astrocytes modulates glutamatergic transmission. Nat Neurosci *19*, 1010-1018.

Szklarczyk, D., Morris, J.H., Cook, H., Kuhn, M., Wyder, S., Simonovic, M., Santos, A., Doncheva, N.T., Roth, A., Bork, P.*, et al.* (2017). The STRING database in 2017: quality-controlled protein-protein association networks, made broadly accessible. Nucleic Acids Res *45*, D362-D368.

Ward, M.E., and Rao, Y. (2005). Investigations of neuronal migration in the central nervous system. Methods Mol Biol *294*, 137-156.

Yan, F., Tan, X., Wan, W., Dixon, B.J., Fan, R., Enkhjargal, B., Li, Q., Zhang, J., Chen, G., and Zhang, J.H. (2017). ErbB4 protects against neuronal apoptosis via activation of YAP/PIK3CB signaling pathway in a rat model of subarachnoid hemorrhage. Exp Neurol *297*, 92-100.

Zhu, G., Chow, L.M., Bayazitov, I.T., Tong, Y., Gilbertson, R.J., Zakharenko, S.S., Solecki, D.J., and Baker, S.J. (2012). Pten deletion causes mTorc1-dependent ectopic neuroblast differentiation without causing uniform migration defects. Development *139*, 3422-3431.

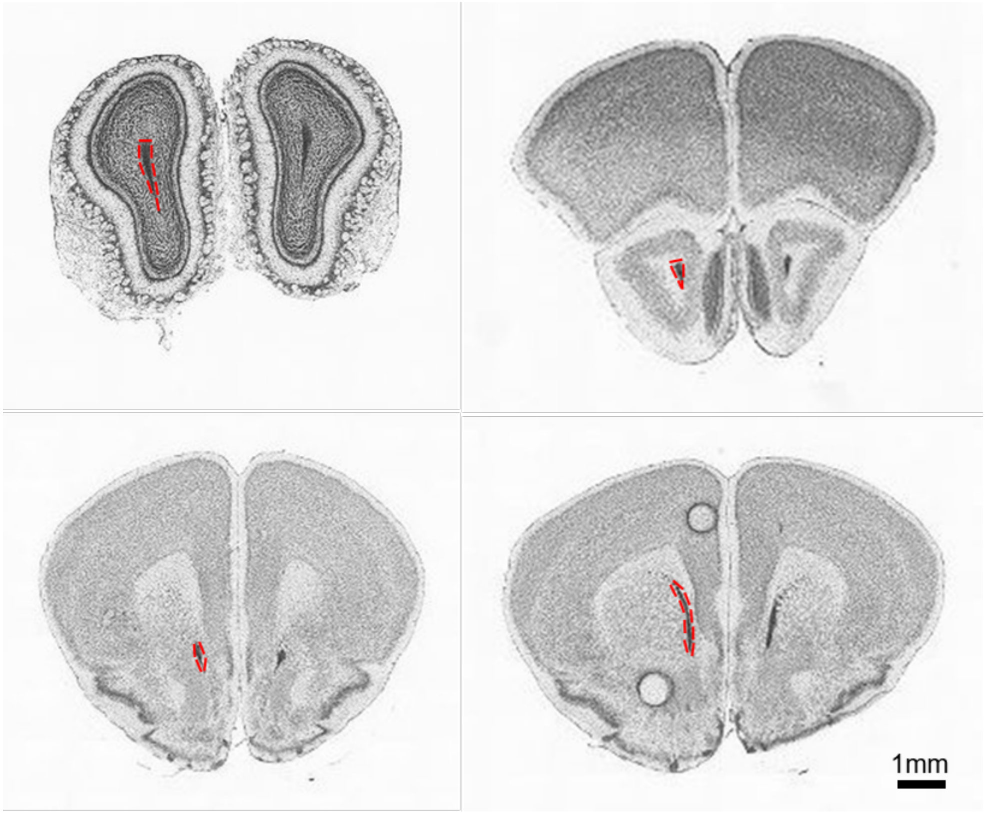

Figure S1: Diagrammatic representation of the regions dissected for the RMS spheroid assay. Nissl stain of representative coronal sections. The RMS outlined in red is 'speckled' in appearance and can be traced back from the centre of the olfactory bulb caudally to the lateral ventricle in a tear drop shape. We avoided dissecting out the anterior commissure which is white in appearance and could be mistaken for the RMS. The rostral half of the olfactory bulb was also excluded as it contains fewer migrating neuroblasts. It was also important not to dissect the SVZ once the ventricle appears to avoid contamination with ciliated ependymal cells, as these may obstruct spheroid formation. Scale bar = 1 mm.

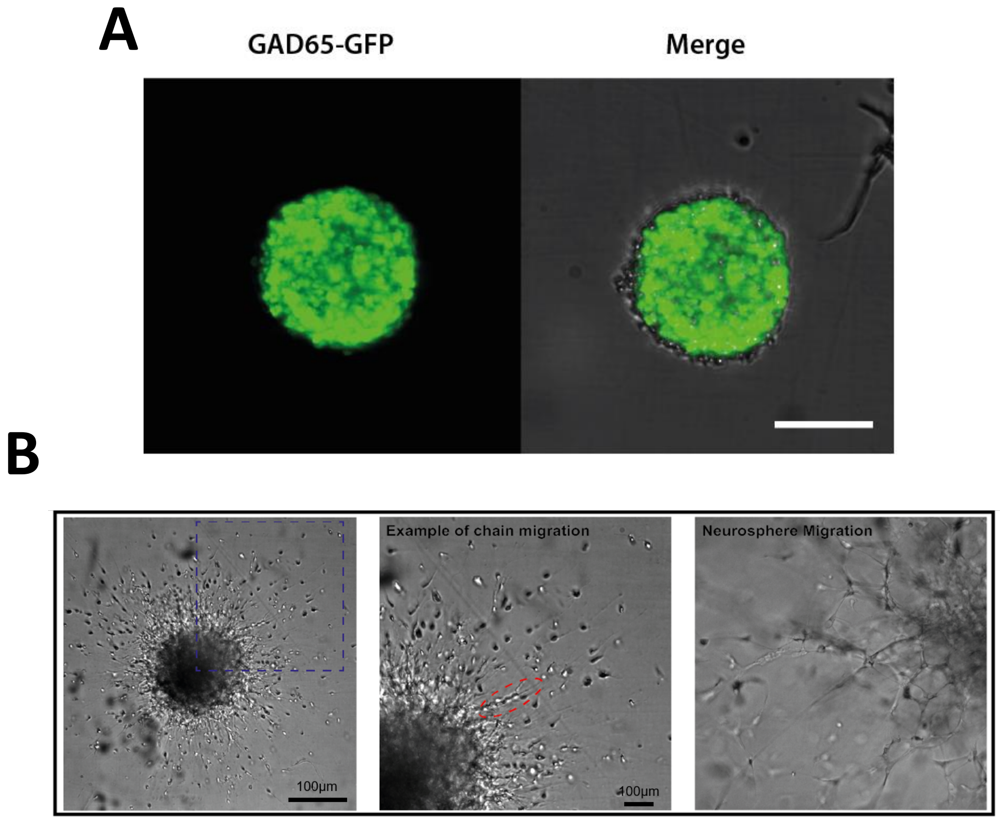

Figure S2.

A: Development of the 3D SVZ spheroid assay using Gad65-GFP mice. Spheroid formation occurred within 24 hours and spheroid shape and size was determined by imaging GFP-labelled cells once transferred onto Matrigel. Right panel shows GFP fluorescence overlap with brightfield. Scale bar = 100 μm.

B: Magnified image of the chain and single cell migration of neuroblasts from an RMS spheroid. Left and central image show cells migrating out as chains from an RMS spheroid. Neuroblasts self-organise into chains to radiate out from the spheroid before continuing as single cells. Scale bars = 100 μm. The right image shows the amoebic glial migration that radiates from a neurosphere as a comparison.

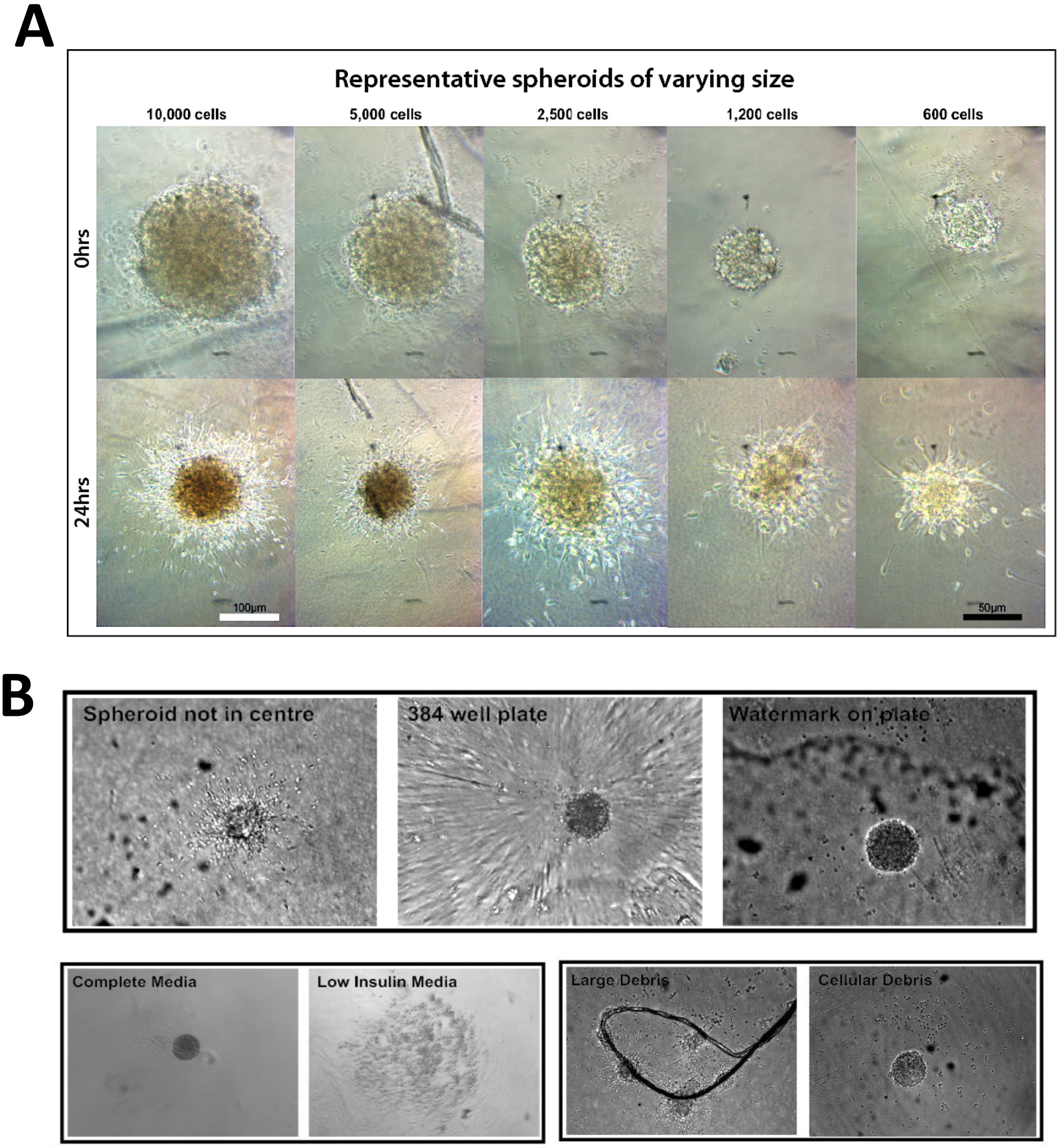

Figure S3.
A: Spheroid size optimisation. Spheroids of between 600 and 10,000 cells were tested for suitability in the 3D assay with imaging at 0 and 24 hours. Larger spheroids showed more migration after 24 hours, but were too large to measure cells migrating the greatest distances. Therefore, intermediate sized spheroids were selected for high throughput screening. The white (100 µm) scale bar on the 5,000 and 10,000 cells reflects a change from a 20x to 10x objective. The black (50 µm) scale bar applies to all other images.
B: Optimising spheroid assay for media composition, debris levels, spheroid positioning and plate suitability. The success of the spheroid assay was dependent on a number of components to ensure spheroid integrity and imaging resolution. These included assessment of the impact on imaging of: spheroids not centred, 384 well plates, watermark on plate, complete media, low insulin media, large debris and cellular debris.

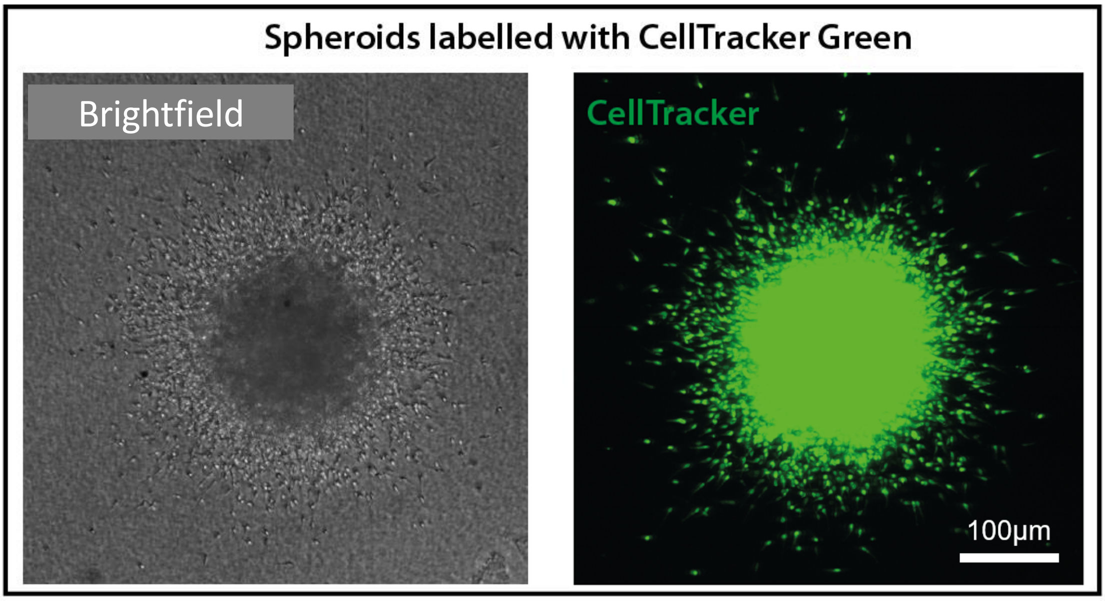

Figure S4. Spheroids labelled with fluorescence dyes. CellTracker Green allows for easy visualisation of spheroids and migrating neuroblasts. Scale bar = 100 µm.

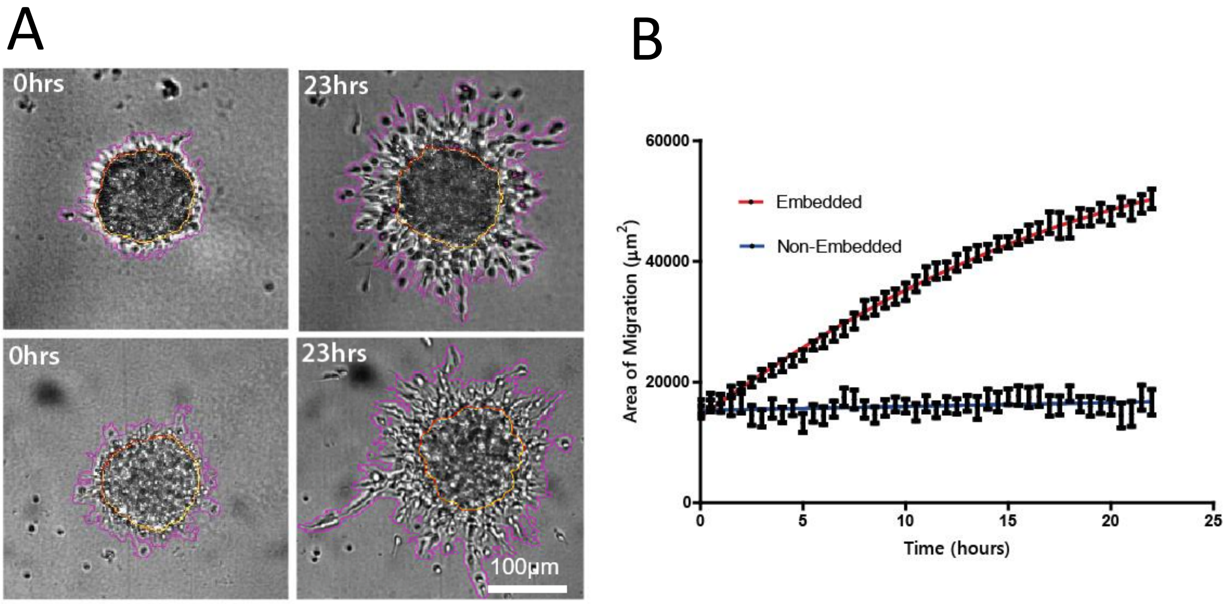

Figure S5: **Segmentation**.
A: Two examples of segmentation of migration area (purple line) around the spheroid (orange line) at 0hrs and 23hrs. Scale bar = 100 μm.
B: The graph shows how the area of migration around 10 spheroids embedded in Matrigel and 10 unembedded spheroids changes over 24 hours.

A

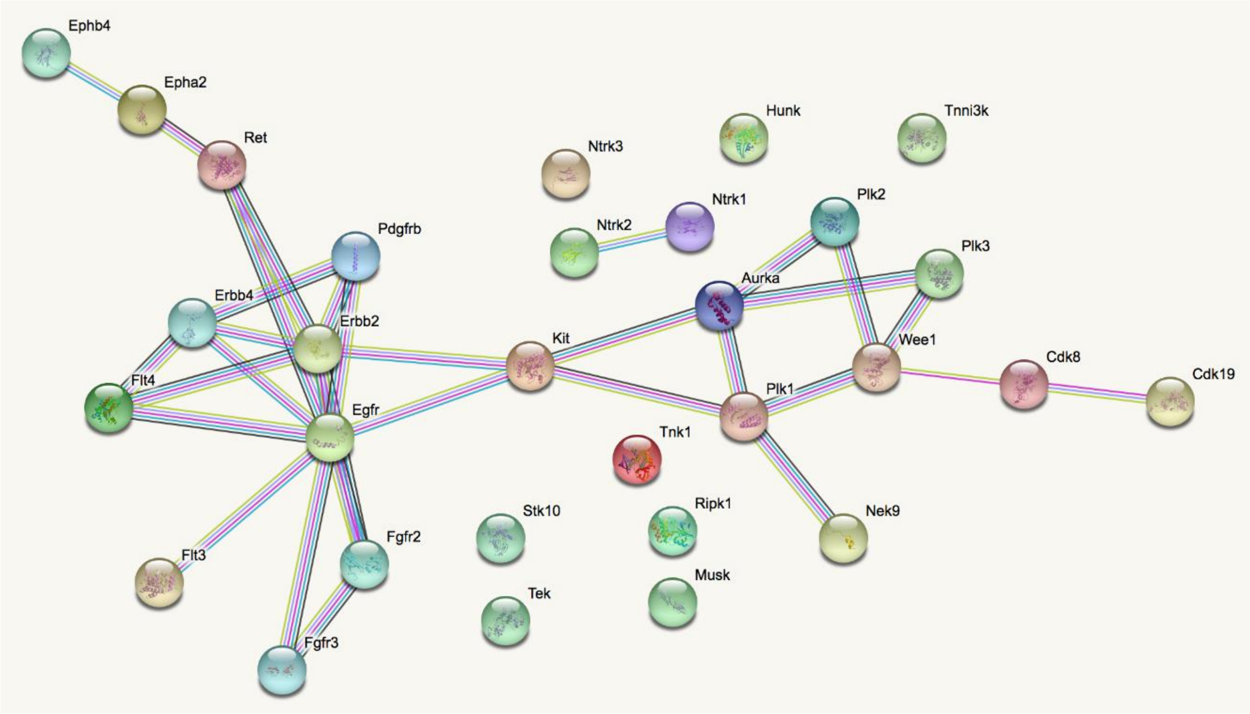

B

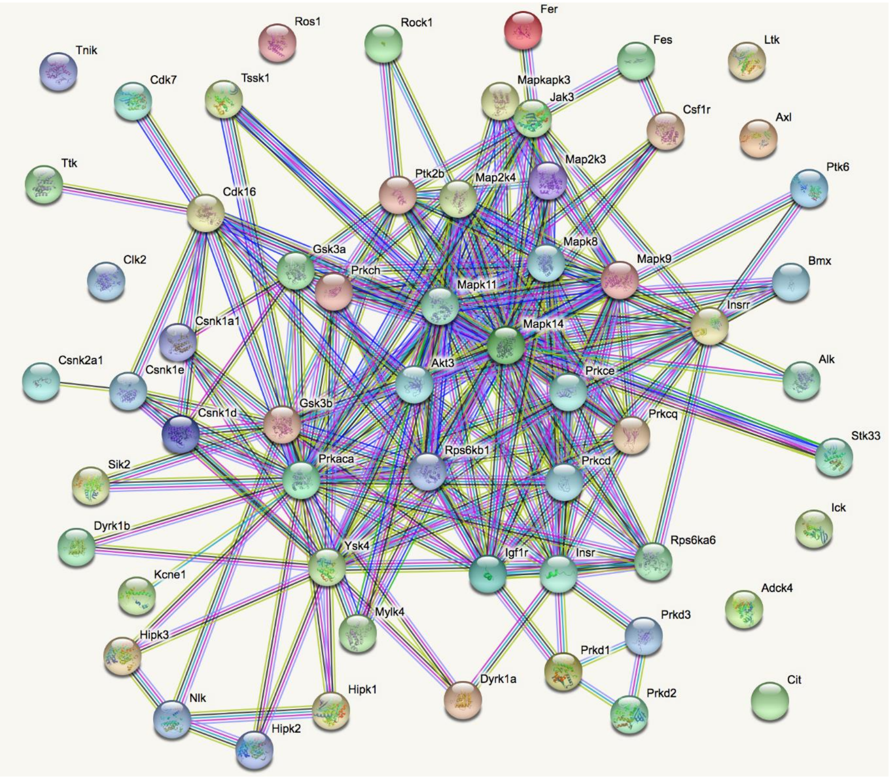

Figure S6

A: STRING representation of kinase targets that increased migration in the PKIS screens

B: STRING representation of kinase targets that decreased migration in the PKIS screens.

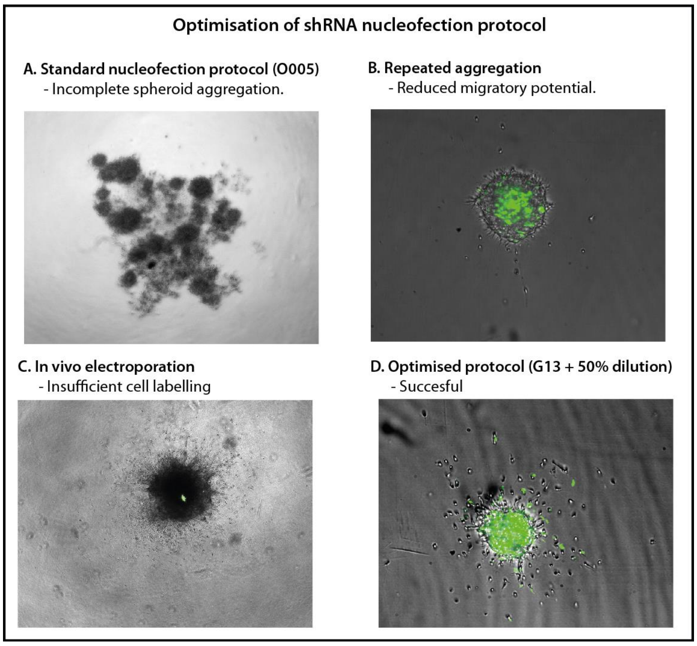

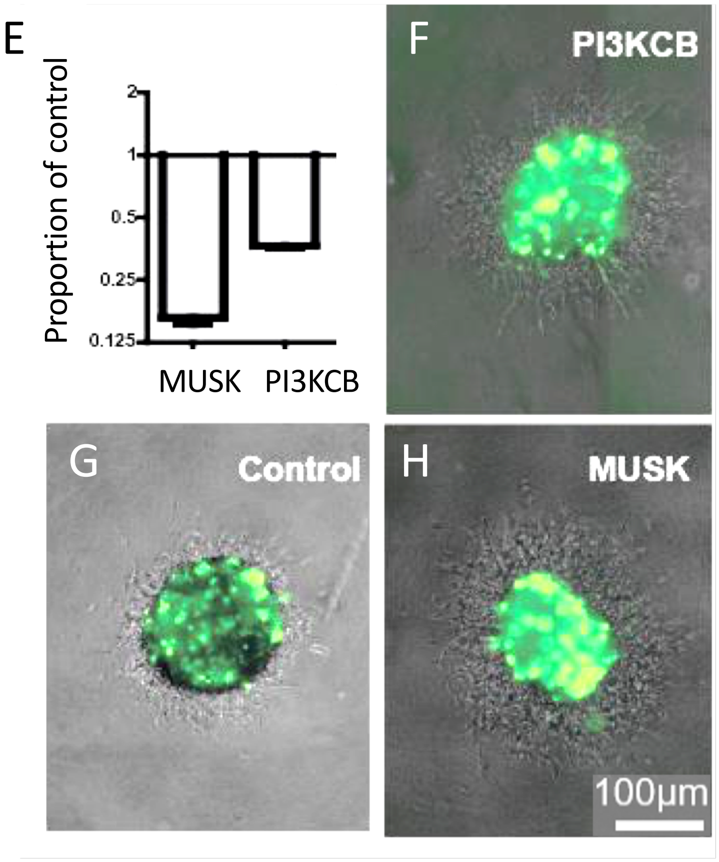

Figure S7

A-D: Optimization of spheroid nucleofection. A-C: Scenarios which were suboptimal. D: Scenario which worked well.

E-H: Knockdown of kinases MuSK and PIK3CB (E). Migration compared to controls increased after both MuSK (H) and PIK3CB KD (F). F-H scale bar = 100 µm.

Table S1. Top kinases selected from analysis of the expression ratio between high and low migrating conditions.

Kinases with highest Δ high:low

PKIS1

Gene	Mean Affinity		Δ high:low
	High	Low	
LOK	25.9	11.9	14.0
FLT3	21.9	8.1	13.8
TRKC	21.8	8.0	13.8
PDGFRα	26.6	13.2	13.4
AURORA-C	21.3	8.5	12.8
TRKB	20.4	7.8	12.6
KIT	18.2	7.4	10.8
TRKA	16.1	6.1	10.0
AURORA-A	18.8	10.9	7.9
EGFR	22.1	14.4	7.7
FGFR2	13.8	6.4	7.4
ERBB4	27.6	20.4	7.2
AURORA-B	15.7	8.5	7.1
RET	21.2	14.6	6.6
FLT4	16.5	10.0	6.5
MUSK	15.5	9.1	6.4
FGFR3	11.4	5.1	6.2
TIE2	16.5	10.4	6.1
NEK9	10.9	5.2	5.7
TNK1	20.3	15.2	5.1
PLK1	13.2	8.2	5.0

PKIS2

Gene	Mean Affinity		Δ high:low
	High	Low	
ERBB2	32.8	20.1	12.7
RIPK1	18.4	8.3	10.0
PLK2	17.2	7.8	9.4
PLK1	15.2	6.3	8.9
MUSK	16.3	7.4	8.9
CDK11	18.4	10.1	8.3
PLK3	13.8	6.7	7.1
EPHB4	16.6	10.0	6.6
CDK8	26.0	19.4	6.6
EGFR	36.8	30.5	6.3
EPHA2	18.1	12.3	5.8
TNNI3K	20.5	14.9	5.6
WEE1	10.5	5.5	5.0
HUNK	14.4	9.4	5.0

Kinases with lowest Δ high:low

PKIS1

Gene	Mean Affinity		Δ high:low
	High	Low	
P38α	11.6	46.3	-34.7
CK1a	4.5	27.8	-23.3
P38β	8.4	27.0	-18.6
PYK2	10.3	26.3	-16.1
JNK2	6.0	20.9	-14.9
INSR	4.6	19.1	-14.6
LTK	4.2	18.1	-14.0
IGF1R	2.7	15.5	-12.9
GSK3B	12.0	24.4	-12.5
FER	4.9	17.2	-12.3
PRKD2	4.6	16.9	-12.3
MAPKAI	4.9	16.9	-12.0
GSK3A	12.3	23.4	-11.1
ALK	5.2	16.1	-10.9
PRKD3	4.0	14.8	-10.8
PRKD1	4.8	15.4	-10.6
ROS	11.6	21.8	-10.2
DYRK1A	7.1	17.1	-10.0
IRR	1.5	11.4	-9.9
CK1-g3	3.1	11.9	-8.9
TSSK1	2.9	11.2	-8.3
DYRK1E	4.0	12.3	-8.3
PKA	2.7	10.7	-8.1
FES	2.6	10.2	-7.7
TTK	10.4	17.1	-6.7
CLK2	7.5	14.2	-6.7
BMX	6.3	12.6	-6.4
CK1-g1	3.2	8.9	-5.7
BRK	9.0	14.4	-5.4
SNF1LK	9.1	14.5	-5.3
MINK	19.2	24.5	-5.2

PKIS2

Gene	Mean Affinity		Δ high:low
	High	Low	
CSNK1A1	11.5	39.9	-28.4
CSNK1E	25.8	51.3	-25.5
CSNK1D	21.5	44.5	-23.0
MEK3	8.3	29.2	-20.9
CSNK1A1	7.2	27.6	-20.4
PRKCH	8.3	28.4	-20.1
HIPK1	8.1	27.1	-19.0
PRKCD	5.0	23.7	-18.7
YSK4	34.1	52.7	-18.6
ROCK1	16.0	33.5	-17.5
HIPK2	5.2	22.7	-17.5
DYRK1B	7.8	24.7	-16.9
HIPK3	12.2	28.9	-16.8
PRKCE	11.4	27.8	-16.4
TNIK	20.4	36.3	-15.9
S6K1	8.3	23.3	-15.0
CSF1R	18.7	33.5	-14.8
AKT3	4.5	19.2	-14.7
CSNK2A1	6.3	20.9	-14.5
MINK	25.8	40.2	-14.4
CIT	31.9	46.3	-14.4
JAK3	13.0	27.3	-14.3
GSK3A	14.2	28.4	-14.2
PCTK1	4.5	18.7	-14.2
MYLK4	13.5	27.6	-14.1
AXL	17.8	31.6	-13.9
STK33	10.3	24.1	-13.8
CDK7	9.9	23.7	-13.7
PKAC-alph	7.1	20.7	-13.6
RSK4	19.1	32.6	-13.5
ADCK4	21.0	34.5	-13.5
PRKCQ	12.7	26.2	-13.5
PRKD1	14.3	27.6	-13.3
MEK4	18.0	31.3	-13.3
ICK	10.8	24.1	-13.3
JNK1	21.7	34.9	-13.2
NLK	22.6	35.6	-13.0

Table S2. Compounds that significantly altered migration from PKIS1.

Compounds that stimulated migration (PKIS1)

Compound	x Control	>90	>80	ABL1	BRK	SRC	YES	EPHA2	EPHA4	DDR2	MUSK	TRKA	TRKC	RET	KDR	KIT	FLT3	PDGFRα	PDGFRβ	TIE2	EGFR	ERBB4	MAP4K4	LOK	PLK1	AURORA-C	NEK9
GW607049C	3.03	0	4	72	1	13	6	90	82	83	79	83	70	74	47	44	33	58	17	71	3		7	73	1	6	0
GW703087X	2.70	0	0	2	2	6	2	2	1	8	7	0	2	11	13	2	2	15	7	7	77	57	3	4	3	15	2
SB-735467	2.43	0	0	69	3	6	7	-2	4	2	5	4	7	4	19	5	4	15	8	7	4	12	70	9	2	7	6
GW300653X	2.39	0	0	7	1	14	9	-2	5	0	4	26	35	34	4	32	44	34	30	3	2	2	62	2	1	7	5
GSK317315A	2.27	1	3	1	-1	0	1	-4	0	-2	22	3	6	4	14	15	8	35	37	5	3	3	4	81	99	0	84
GW627512B	2.23	3	4	22	4	72	47	-1	1	9	14	23	25	16	48	99	91	70	74	5	14	-3	26	11	2	72	8
GSK237701A	2.22	5	9	58	4	41	18	7	27	12	78	12	63	52	72	27	25	100	95	42	3	14	19	88	97	51	92
GW627834A	2.04	0	0	10	3	20	18	1	8	3	3	13	25	4	11	80	62	17	26	2	2	9	24	4	2	6	5
GW804482X	1.99	1	1	1	1	-3	1	2	3	0	-4	-8	-2	7	3	-4	-4	14	8	3	2	13	18	39	92	4	8
GSK259178A	1.96	0	1	4	5	9	8	0	3	3	5	1	3	5	11	2	3	12	8	11	76	86	6	8	11	9	12
GW824645A	1.95	0	1	9	4	10	12	1	10	3	14	19	28	18	15	85	67	36	38	5	3	11	70	16	3	0	9
GW794607X	1.94	0	7	86	51	82	83	5	17	8	50	7	37	55	65	24	18	78	79	36	33	43	44	28	1	59	0
GW701427A	1.94	0	0	15	0	8	2	-5	5	49	3	3	4	14	13	19	6	39	16	7	-2	0	6	7	1	38	2
SB-437013	1.93	0	1	12	42	9	8	2	8	4	3	2	2	9	18	-1	2	10	4	83	3	23	8	78	4	15	0
GW683134A	1.91	7	19	94	4	37	22	83	87	80	83	85	90	84	72	92	71	85	58	82	-1	-5	5	77	1	82	7
GW693881A	1.90	0	1	5	6	7	9	-6	6	-1	6	3	2	12	12	2	2	16	9	9	77	69	14	12	4	-2	9
SB-633825	1.88	1	2	43	85	11	21	1	18	-5	6	1	4	22	48	5	1	24	13	79	6	44	15	95	4	13	18
GSK300014A	1.86	0	1	5	6	15	6	-1	10	6	9	7	4	7	9	8	7	14	11	11	84	77	10	13	8	9	9
GW780159X	1.83	0	0	8	11	14	18	1	15	-6	2	0	5	6	67	8	2	28	15	6	7	28	60	14	4	-2	12

Compounds that inhibited migration (PKIS1)

Compound	x Control	>90	>80	YES	PYK2	LTK	ALK	INSR	IGF1R	DDR2	PDGFRβ	ERBB4	CK1a	PRKD3	PRKD2	TSSK1	CDK2	CDK3	JNK2	P38α	GSK3A	GSK3B	CLK2	DYRK1A	TTK
GW784752X	0.34	6	19	73	16	6	8	7	5	9	81	21	11	9	9	5	77	49	11	16	90	89	35	72	19
SB-686709-A	0.33	0	2	0	1	0	2	1	-1	1	5	1	-1	0	0	-1	20	17	2	-1	90	89	3	61	2
GW811168X	0.32	5	10	57	6	2	-2	1	-5	8	78	8	6	-2	-3	-1	68	21	5	5	81	79	18	64	-19
GW827099X	0.30	5	15	97	44	6	8	18	3	83	70	95	73	48	52	3	4	-3	85	93	60	76	1	3	19
SB-253228	0.30	0	1	0	12	2	2	0	-5	2	11	5	15	6	4	3	-1	-1	4	84	3	3	0	0	14
SB-678557-A	0.30	0	2	3	15	5	7	5	4	1	8	24	4	5	6	7	6	6	6	5	86	84	2	34	15
SB-226879	0.30	0	1	9	18	5	5	3	5	3	21	12	73	11	15	7	-1	0	37	86	5	7	2	4	2
SB-253226	0.28	0	0	3	7	4	5	4	6	2	10	14	21	5	5	5	3	-2	6	71	3	2	0	4	15
GW833373X	0.28	2	10	92	48	3	6	8	5	83	68	89	71	81	87	0	1	3	81	89	45	67	2	5	6
GSK1000163A	0.28	0	0	6	-7	0	0	0	-1	6	12	0	-1	6	7	10	-1	-1	-2	0	22	19	1	2	5
GSK1392956A	0.27	3	7	12	97	89	70	117	93	1	5	16	13	21	17	85	4	-3	57	10	0	-1	83	13	82
SB-220025-A	0.27	1	2	16	4	1	2	5	4	0	26	9	86	26	38	-1	6	-1	7	91	4	6	2	4	0
SB-223133	0.27	2	2	40	2	1	3	0	-1	13	30	46	90	19	23	-2	-5	-1	53	93	6	14	1	6	0
GW829906X	0.26	2	6	92	59	3	4	25	5	47	85	79	59	51	57	4	3	-1	82	74	68	76	3	3	11
SB-220455	0.25	0	1	13	6	5	4	5	3	2	30	19	73	17	16	3	1	0	11	83	7	8	14	6	-8
SB-239272	0.24	0	1	-1	7	2	0	3	-4	2	6	15	31	2	4	2	-2	-2	14	86	-2	-2	-1	-2	6
GW276655X	0.24	1	3	12	20	3	0	7	-2	2	50	14	3	7	9	12	94	89	2	3	67	65	36	81	53
SB-251505	0.24	0	1	7	14	3	4	6	4	4	12	11	32	6	5	4	2	0	13	80	4	4	3	6	8
GSK1751853A	0.23	1	5	1	93	81	82	88	88	-1	5	60	11	8	9	32	2	-1	18	7	2	4	45	10	48
GSK2186269A	0.22	3	7	-2	90	90	85	91	98	1	3	6	2	4	4	19	-2	2	2	4	1	1	44	4	25

Table S3. Compounds that significantly altered migration from PKIS2.

Compounds that stimulated migration (PKIS2)

Compound	x Control	>90	>80	AURKA	CDC2L5	CDKL2	EGFR	ERBB2	ERK8	FLT4	GRK1	HUNK	KIT	MEK5	MYLK2	NLK	PDGFRB	PIK3CB	PLK1	PLK2	PLK3	RET	RIOK2	SGK2	SLK	SRPK1	YSK4
GSK306886	2.43	5	5	30	0	19	99	99	0	98	4	57	34	26	3	44	46	0	0	0	0	98	3	0	8	100	0
GW579362	2.40	75	124	100	0	41	55	17	18	100	2	8	100	100	32	98	100	0	0	0	4	100	66	0	99	99	99
GSK175726	2.40	3	4	90	0	32	38	22	0	0	100	0	40	49	11	17	21	40	0	3	1	10	92	0	8	0	0
GSK346294	2.37	2	3	8	0	5	32	15	0	1	0	0	20	18	0	19	22	5	100	96	87	24	0	10	59	20	3
GW466413	2.18	1	4	0	0	5	28	35	8	9	14	93	17	35	0	0	5	0	0	0	0	24	2	0	8	21	11
GSK2336394	2.15	1	1	0	0	22	28	20	0	3	0	6	14	1	0	0	15	98	0	0	0	14	6	1	22	37	26
SB-601273	2.11	138	164	62	41	100	88	93	0	96	6	55	100	100	98	99	100	30	26	27	52	100	29	2	99	50	99
GSK1024304	2.08	3	5	2	0	18	4	28	0	1	0	14	23	32	0	0	31	100	97	90	86	26	80	10	9	29	15
GW681170	2.05	32	38	78	69	98	12	22	95	79	0	7	100	51	0	30	100	5	0	0	0	100	0	1	36	25	87
SB-735464	2.03	0	2	18	23	57	0	21	32	0	14	0	33	31	77	86	67	4	0	37	13	0	40	0	11	36	82
SB-732932	2.00	4	9	13	47	95	0	0	92	0	0	0	56	50	97	46	80	8	2	24	0	0	16	4	0	45	95
GW854278	1.97	5	8	0	5	0	27	11	0	68	0	0	24	99	0	0	58	0	100	100	99	20	14	3	96	44	61
SB-733416	1.97	1	1	0	98	9	37	0	23	27	6	0	8	37	0	11	16	0	0	0	0	23	0	9	28	11	0
GW824645	1.95	3	4	4	0	0	0	4	3	0	23	58	90	70	9	0	96	0	0	20	23	0	0	100	0	0	0
GW683134	1.91	41	54	0	0	85	44	9	82	100	0	0	100	85	75	6	100	0	0	0	0	100	7	14	83	51	93
GW659009	1.81	14	22	0	0	15	100	93	10	0	0	0	17	98	0	0	36	0	0	0	30	0	53	4	0	37	3

Compounds that inhibited migration (PKIS2)

Compound	x Control	>90	>80	AMPK-alpha1	AXL	CDKL2	DAPK3	ERK8	FLT3	GSK3A	ICK	IGF1R	LYN	MYLK2	MYLK4	NIM1	PDGFRB	PHKG2	PIK3C2B	PIK3C2G	PIK3CB	PIK3CD	PIK3CG	PLK1	PLK2	PLK3	TXK	VPS34	ZAK	p38-alpha
GSK1440913	0.34	3	4	17	51	89	5	99	48	94	79	0	20	76	100	12	60	15	0	24	0	47	67	0	0	0	35	70	16	0
SB-738481	0.34	5	8	0	37	87	0	84	92	93	95	0	11	89	19	100	90	0	7	0	13	5	12	3	3	12	20	0	15	0
GSK2373693	0.33	5	6	0	4	11	95	26	0	0	15	10	0	6	2	11	65	0	99	89	0	100	94	0	0	0	0	97	15	66
GSK1660437	0.33	3	3	48	68	99	6	66	33	12	0	0	74	0	0	0	99	0	0	0	13	0	0	0	5	0	20	0	97	32
SB-711880	0.32	3	6	90	18	99	21	82	28	87	26	0	0	85	0	0	0	100	14	0	0	0	17	0	0	0	0	0	0	0
SB-229482	0.31	0	1	0	48	22	0	0	27	0	45	0	15	21	11	54	29	16	0	0	37	0	34	11	0	0	0	50	4	89
GW813349	0.30	3	4	0	2	13	0	9	8	17	0	9	33	0	24	5	0	0	24	17	0	0	24	97	92	93	9	84	0	0
GSK1287544	0.29	2	3	17	74	40	0	43	83	0	23	2	0	30	100	15	28	0	0	0	100	0	0	0	14	0	18	28	59	0
GSK1379874	0.28	3	3	0	10	5	0	0	0	0	0	0	0	0	0	33	26	0	0	0	0	0	0	0	0	0	100	16	100	20
GSK1383281	0.28	20	35	57	74	11	37	2	68	7	11	4	0	1	79	0	74	16	0	0	0	0	0	0	13	38	79	0	73	0
SB-219980	0.28	9	11	0	9	0	0	0	0	16	1	0	0	0	0	10	20	0	17	52	4	35	7	12	28	10	0	0	0	100
SB-742034-AC	0.27	1	6	9	13	69	17	2	7	98	22	0	2	89	25	0	30	4	16	13	2	0	4	3	24	5	0	0	0	13
GSK2286295	0.27	4	5	0	100	7	15	2	0	8	3	12	98	2	0	0	37	19	22	0	99	89	50	10	0	0	19	97	6	0
GW494601	0.26	36	53	0	21	2	15	0	28	23	0	13	86	24	0	0	100	0	13	2	0	22	0	30	0	0	98	34	84	50
GSK301362	0.26	7	10	6	47	30	40	0	47	0	14	0	4	40	99	15	67	0	0	0	0	6	2	10	34	0	0	0	0	0
GSK2358994	0.24	2	3	5	3	0	6	13	10	4	4	0	0	0	0	0	2	0	70	64	100	82	45	0	0	16	7	100	12	0
GW560116	0.24	7	9	0	0	2	25	0	0	0	0	24	0	27	5	0	95	0	0	0	0	0	0	0	0	0	23	22	0	36
GSK1581427	0.24	33	54	55	86	18	19	30	68	0	24	0	0	19	100	22	75	19	0	0	0	0	0	0	0	0	1	0	0	0
GSK2373690	0.23	2	2	0	0	24	8	0	0	13	1	41	0	2	100	0	14	0	0	0	97	65	21	0	0	0	3	59	9	0
GW787226	0.23	22	30	17	73	39	0	3	99	0	0	34	84	0	0	0	100	0	5	0	0	0	0	0	0	15	17	0	17	0
SB-219952	0.22	10	11	100	19	0	0	0	24	0	70	0	9	20	21	31	23	0	56	69	0	16	64	23	2	0	16	35	9	95
GSK986310C	0.22	12	29	39	59	28	59	0	23	24	16	0	34	7	94	18	80	44	0	0	0	0	3	1	0	0	0	56	0	0
SB-222903	0.21	6	7	0	0	0	25	6	0	3	0	0	0	0	0	0	17	0	0	0	0	0	0	0	0	4	0	0	0	99
SB-249175	0.21	14	18	1	54	0	0	2	39	0	65	0	12	0	19	44	8	0	23	1	0	18	45	9	24	23	10	24	15	99
GSK2276055	0.20	11	14	0	0	0	23	0	0	98	31	0	0	3	48	30	63	6	9	0	11	3	21	7	18	0	76	0	69	8
GSK1723980	0.20	84	99	74	74	99	0	98	66	74	32	0	98	0	0	0	100	0	7	0	0	0	0	0	0	0	93	0	99	27
SB-710363	0.19	18	27	0	37	97	32	90	82	99	99	0	23	79	31	0	32	0	0	0	0	0	2	0	10	0	0	0	23	0
GSK2008607	0.18	115	145	97	98	99	34	94	70	53	77	8	99	0	0	1	100	0	49	0	0	0	0	0	0	4	95	0	99	81
GSK1389063	0.18	67	100	91	95	40	18	51	98	0	64	0	0	52	99	13	96	24	0	0	52	2	0	0	27	27	30	35	0	0
GSK1383280	0.17	25	41	49	63	0	28	3	69	0	18	23	0	22	86	20	75	20	0	0	0	0	0	0	30	47	0	0	15	11
GSK2181306	0.16	127	170	89	100	44	100	5	61	98	68	19	7	78	60	67	100	94	12	23	100	34	86	31	5	0	26	88	0	0
GSK1379722	0.16	6	6	2	0	30	5	31	1	0	100	100	0	12	0	15	8	0	0	0	0	0	0	0	0	0	16	0	0	6

Table S4. Kinase inhibitors that increased migration of neuroblasts from 3D spheroids. Compounds are listed together with their target (if known), chemical structure, representative image of migration at 24hrs, and the extent of migration above the DMSO control for 5 different concentrations of the compound.

Compound	Chemical Structure	Migration Image	Results
**SB-735467**<br><br>Intended Target:<br><br>**GSK3b**	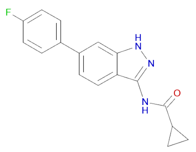	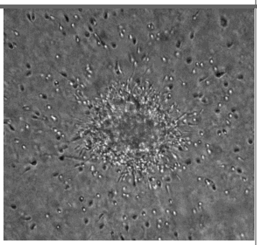	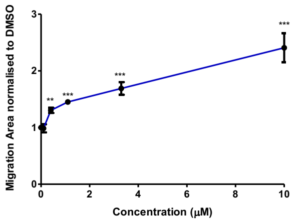
**GW701427A**<br><br>Intended Target:<br>**TIE2/VEGFR2**	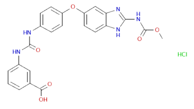	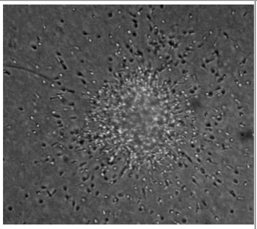	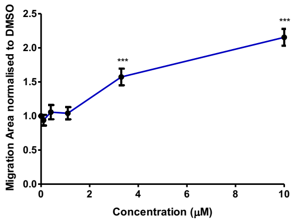
**GW693881A**<br><br>Intended Target:<br>**EGFR/ERBB2**	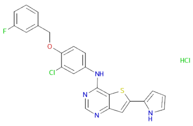	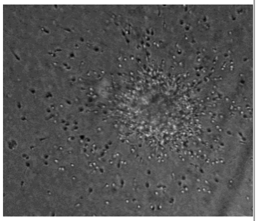	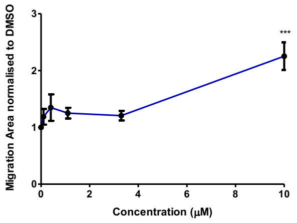
**GW681170A**<br><br>Intended Target:<br>**TIE2/VEGFR**	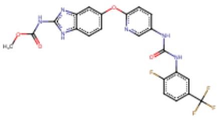	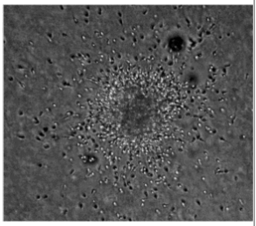	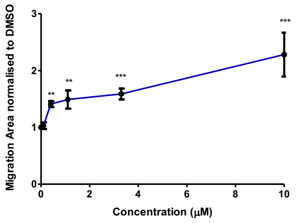
**SB-732932**<br><br>Intended Target:<br>**GSK3a/GSK3b**	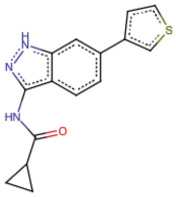	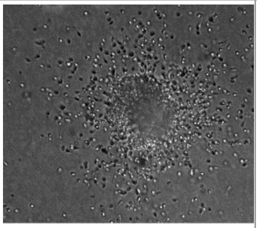	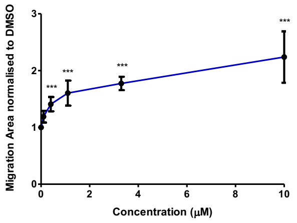
**GW854278X**<br><br>Intended Target:<br>**PLK1/PLK2**	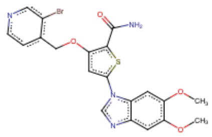	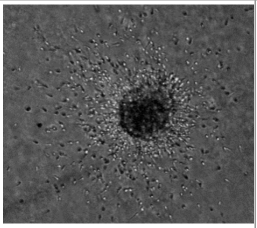	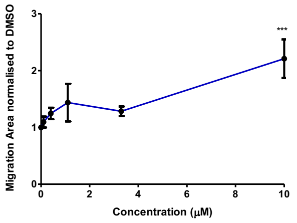

**GW869641X**<br><br>Intended Target:<br>**ALK5**	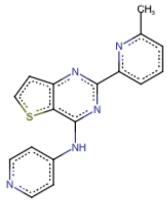	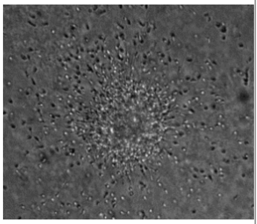	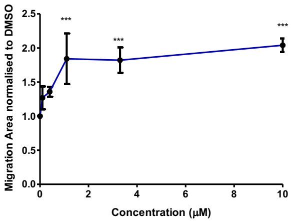
**GW579362A**<br><br>Intended Target:<br><br>**Unknown**	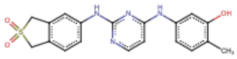	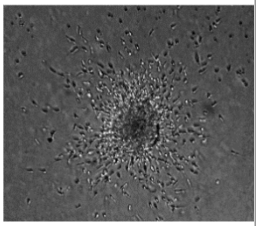	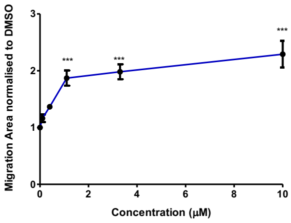
**GW659008A**<br><br>Intended Target:<br>**Unknown**	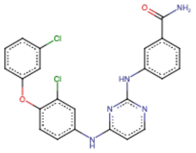	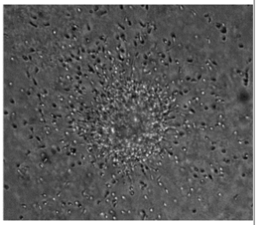	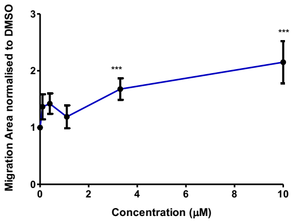
**GW459135A**<br><br>Intended Target:<br>**EGFR/ERBB2**	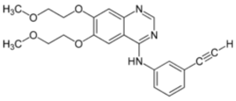	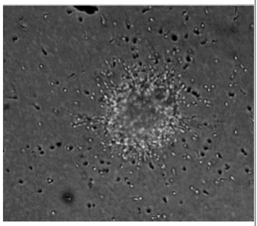	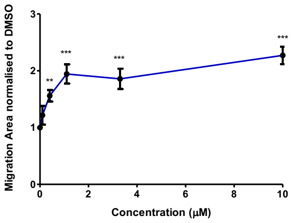
**GW703087X**<br><br>Intended Target:<br>**EGFR**	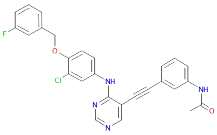	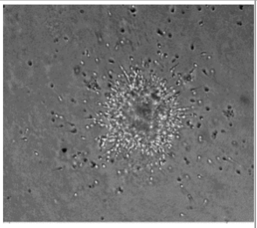	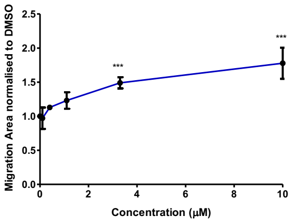
**GW707818B**<br><br>Intended Target:<br>**EGFR/ERBB2**	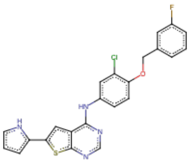	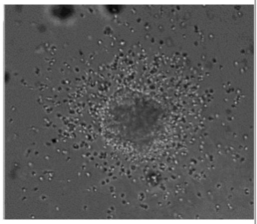	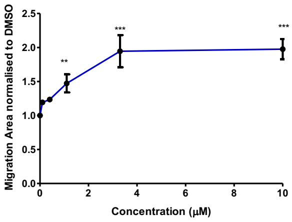
**GSK336313A**<br><br>Intended Target:<br>**Unknown**	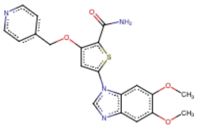	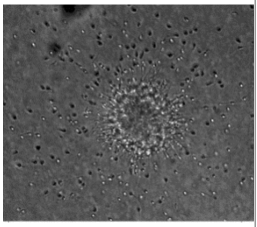	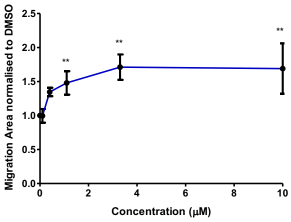

**GSK346294A**<br><br>Intended Target:<br>**PLK1/PLK2**	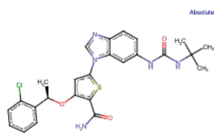	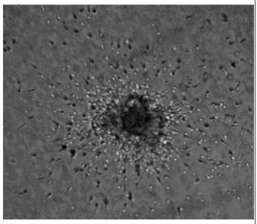	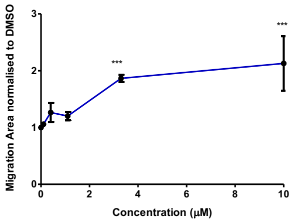
**GSK1024304A**<br><br>Intended Target:<br>**PLK1/PLK2**	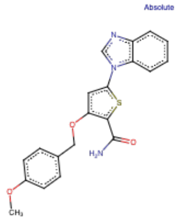	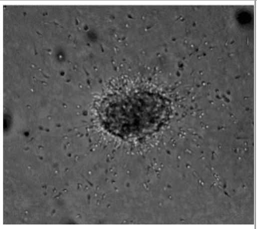	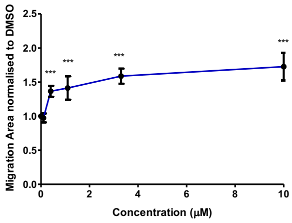
**GSK259178A**<br><br>Intended Target:<br>**EGFR/ERBB2**	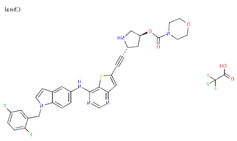	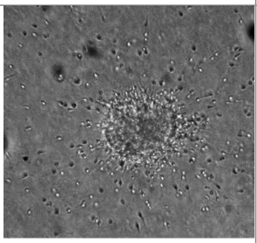	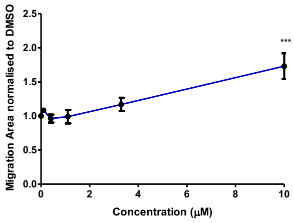
**SB-733416**<br><br>Intended Target:<br>**MSK-1**	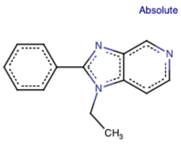	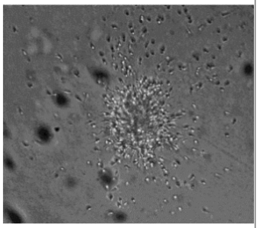	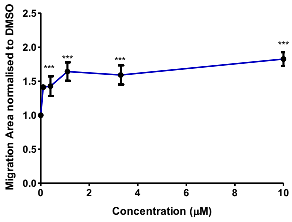
**GW607049C**<br><br>Intended Target:<br>**TIE2/VEGFR2**	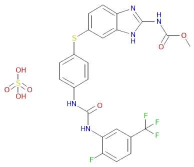	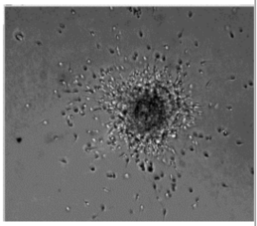	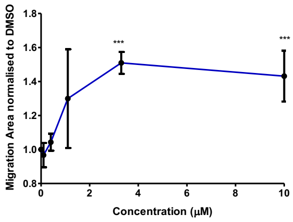
**GW627512B**<br><br>Intended Target:<br>**VEGFR2**	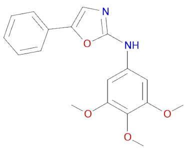	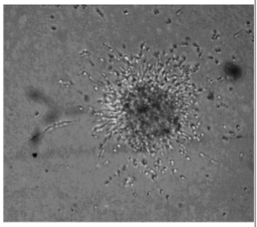	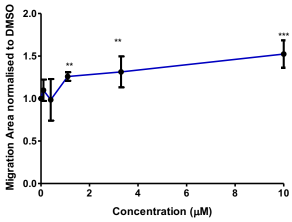
**GSK2336394A**<br><br>Intended Target:<br>**PIK3CB**	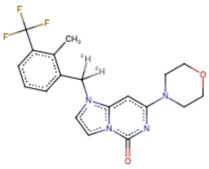	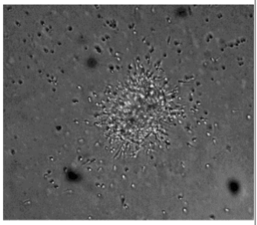	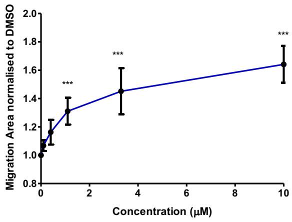

**GW300653X**<br><br>Intended Target:<br>**CDK2**	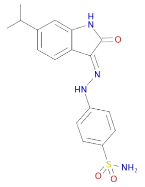	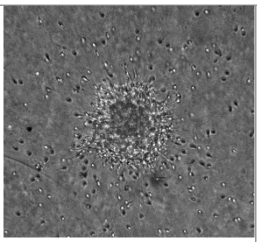	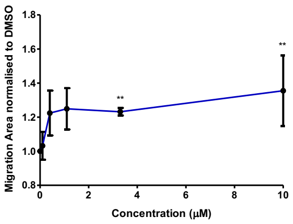

Table S5. Kinase inhibitors that decreased migration of neuroblasts from 3D spheroids. Compounds are listed together with their target (if known), chemical structure, representative image of migration at 24 hours, and distance of migration measured compared to the DMSO control for 5 different concentrations of the compound.

Compound	Chemical Structure	Migration Image	Results
**SB-251505**<br><br>Intended Target:<br>**p38a**	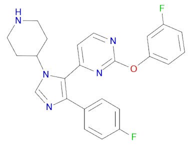	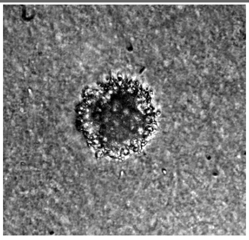	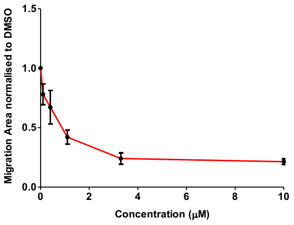
**SB-220455**<br><br>Intended Target:<br>**p38a**	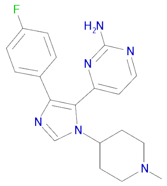	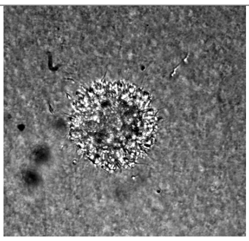	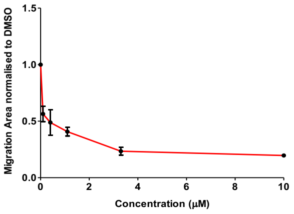
**SB-253228**<br><br>Intended Target:<br>**p38a**	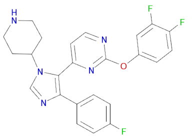	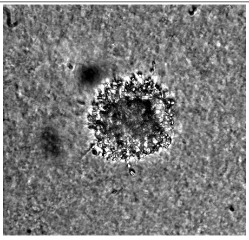	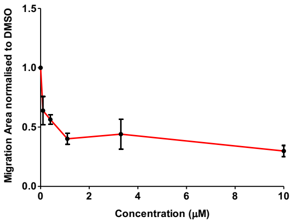
**SB-226879**<br><br>Intended Target:<br>**p38a**	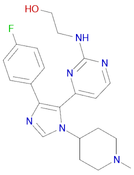	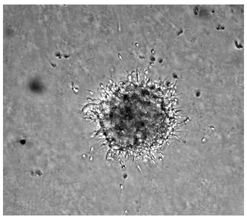	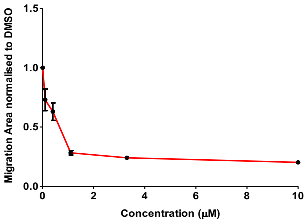
**SB-239272**<br><br>Intended Target:<br>**p38a**	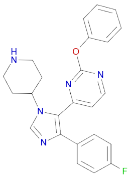	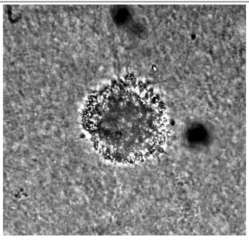	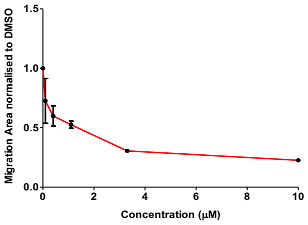
**SB-219980**<br><br>Intended Target:<br><br>**P38a/p38b**	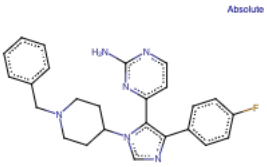	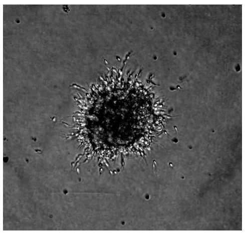	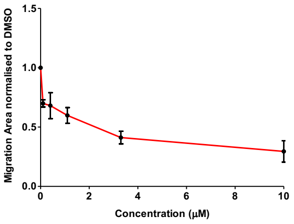

**SB-253226**<br><br>Intended Target:<br>**p38α**	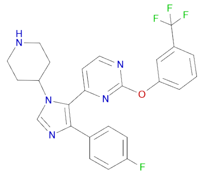	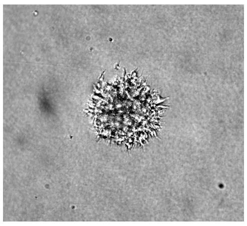	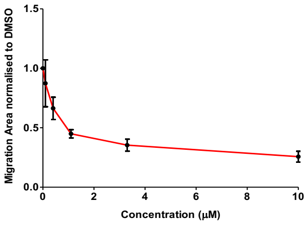
**SB-222903**<br><br>Intended Target:<br><br>**P38a/p38b**	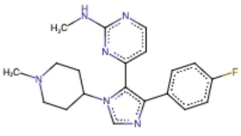	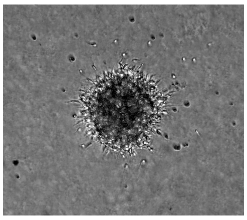	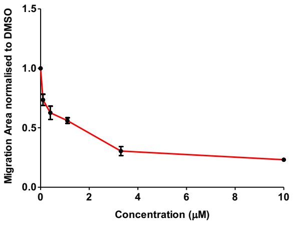
**SB-219952**<br><br>Intended Target:<br><br>**P38a/p38b**	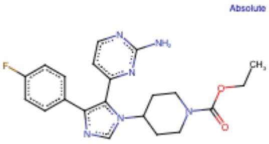	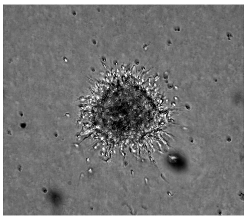	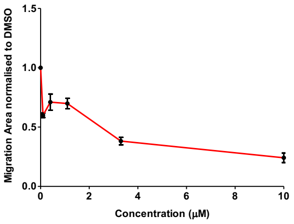
**SB-226605**<br><br>Intended Target:<br><br>**P38a/p38b**	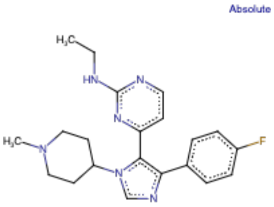	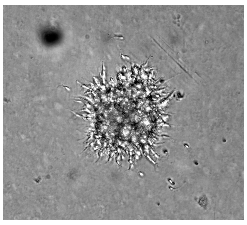	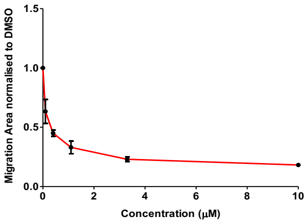
**GW833373X**<br><br>Intended Target:<br>**GSK3b**	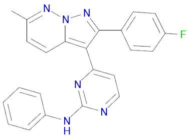	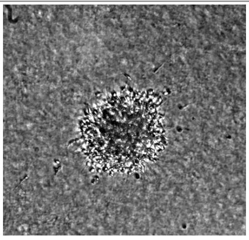	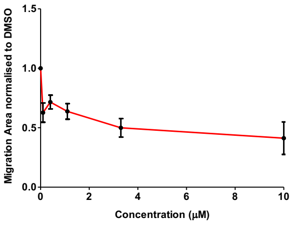
**SB-742034-AC**<br><br>Intended Target:<br><br>**GSK3a/GSK3b**	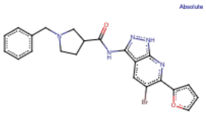	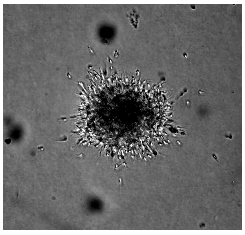	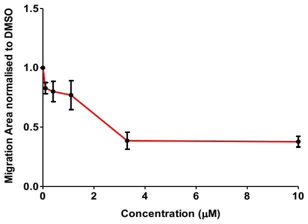
**SB-678557-A**<br><br>Intended Target:<br>**GSK3a/GSK3b**	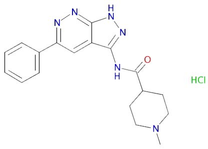	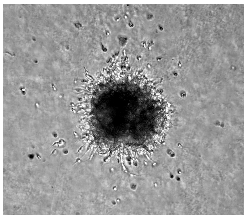	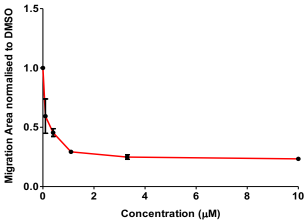

**GW784752X**<br><br>Intended Target:<br><br>**GSK3b**	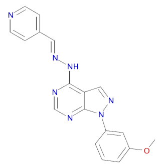	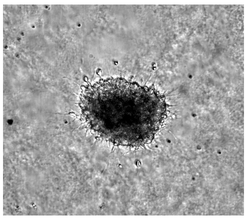	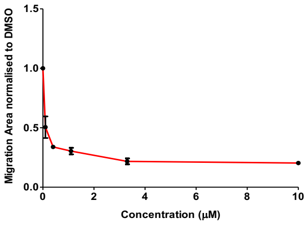
**SB-627772-A**<br><br>Intended Target:<br><br>**GSK3a/GSK3b**	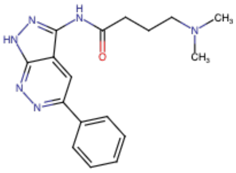	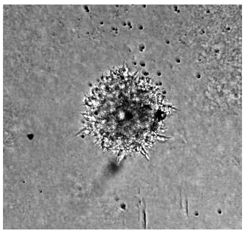	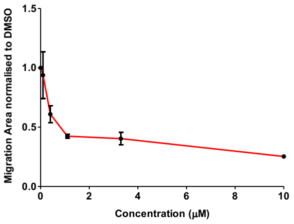
**GW829906X**<br><br>Intended Target:<br>**GSK3b**	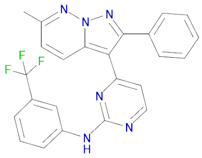	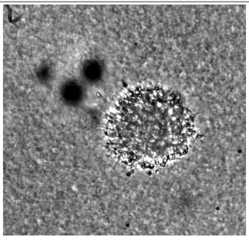	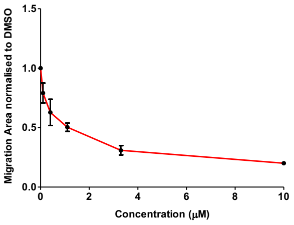
**GSK2358994A**<br><br>Intended Target:<br><br>**PI3K**	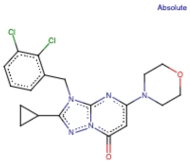	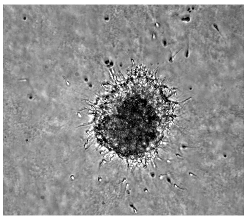	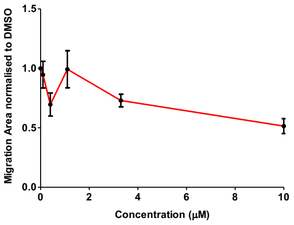
**GSK2286295A**<br><br>Intended Target:<br><br>**PI3K**	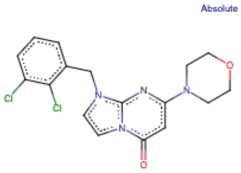	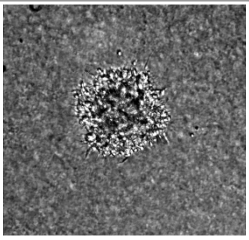	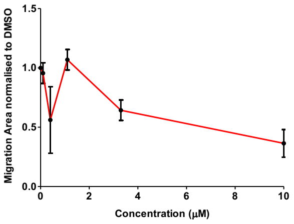
**GSK2373690A**<br><br>Intended Target:<br><br>**PI3K**	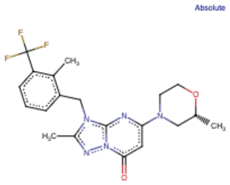	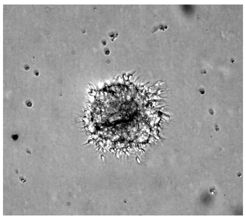	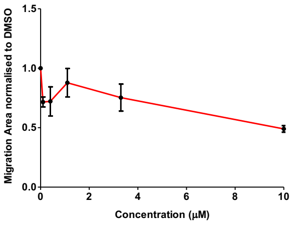
**GSK1389063A**<br><br>Intended Target:<br><br>**Akt**	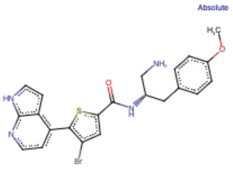	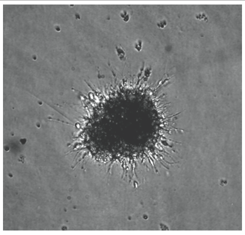	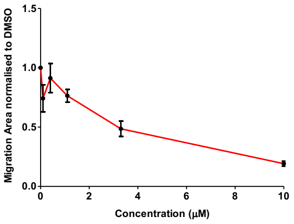

**GSK1000163A**<br><br>Intended Target:<br>**Akt3**	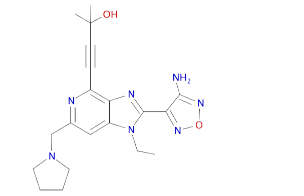	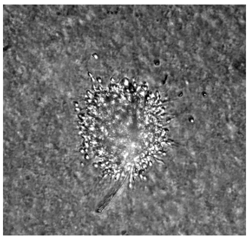	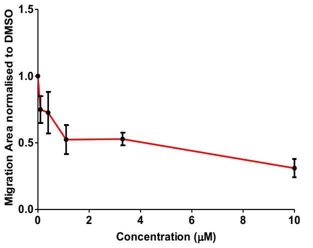
**GSK1383281A**<br><br>Intended Target:<br><br>**Akt**	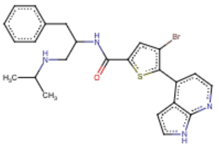	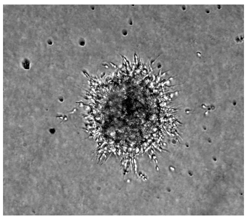	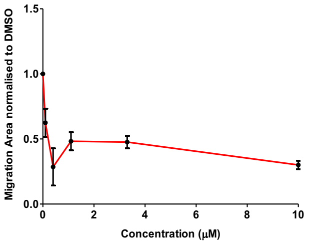
**GSK1383280A**<br><br>Intended Target:<br><br>**Akt**	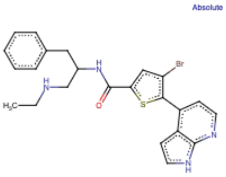	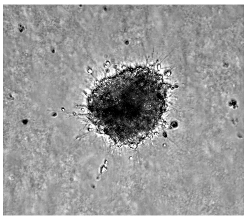	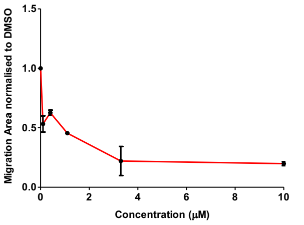
**GW856804X**<br><br>Intended Target:<br><br>**TIE2/VEGFR2**	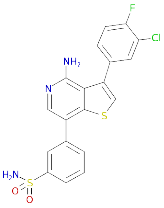	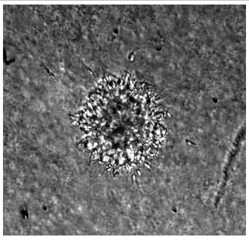	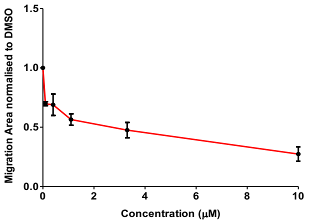
**GW787226A**<br><br>Intended Target:<br><br>**VEGFR**	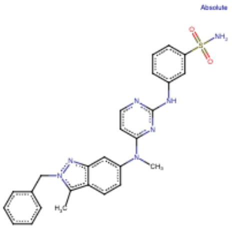	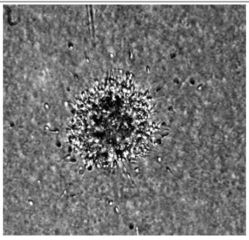	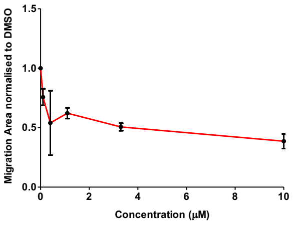
**GSK1819799A**<br><br>Intended Target:<br>**IGF1R**	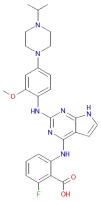	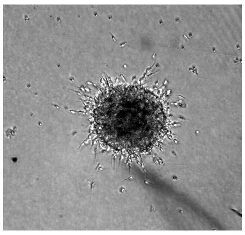	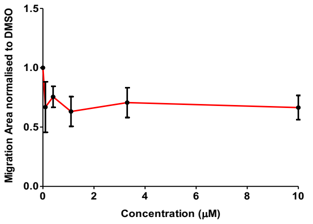
**GW276655X**<br><br>Intended Target:<br>**CDK2**	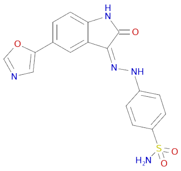	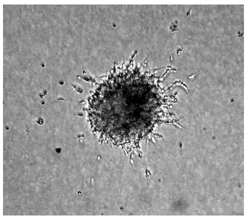	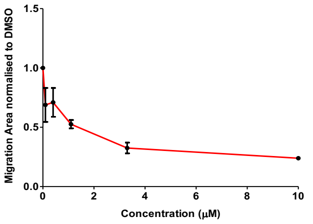

**GSK301362A**<br><br>Intended Target:<br><br>**IKK**	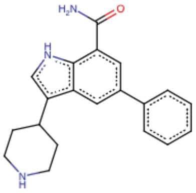	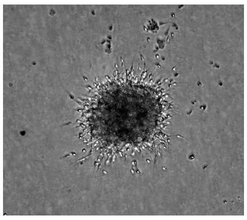	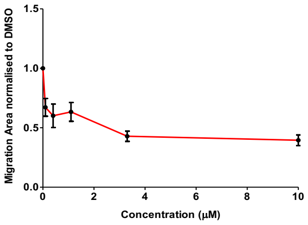
**GW813349X**<br><br>Intended Target:<br><br>**PLK1/PLK2**	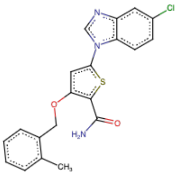	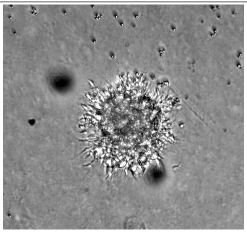	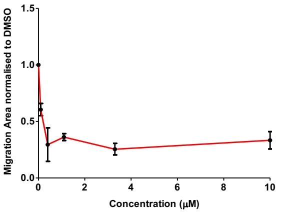
**GSK1440913A**<br><br>Intended Target:<br><br>**Unknown**	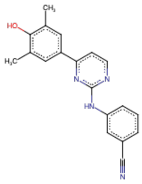	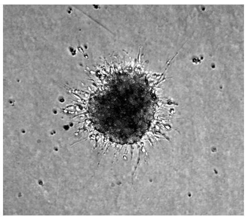	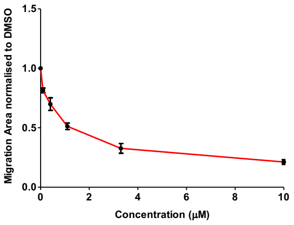
**SB-477794-AAA**<br><br>Intended Target:<br><br>**Unknown**	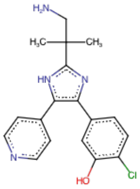	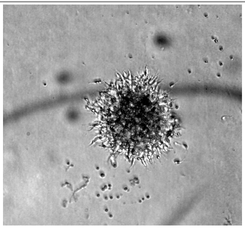	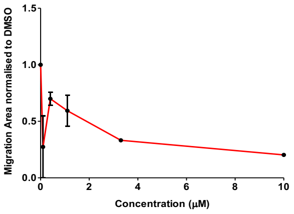
**GW494601A**<br><br>Intended Target:<br><br>**Unknown**	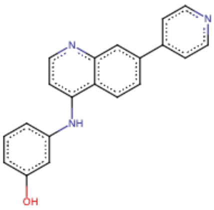	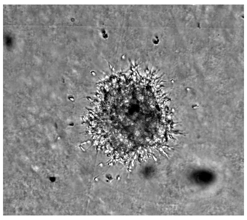	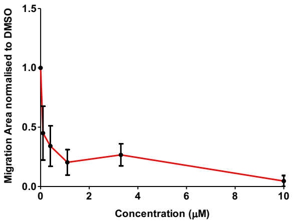
**GSK2181306A**<br><br>Intended Target:<br><br>**Unknown**	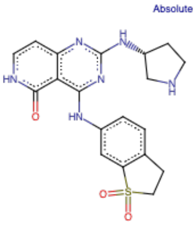	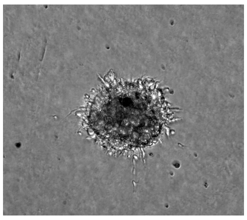	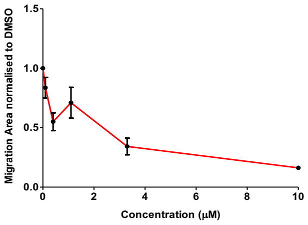
**GSK1379874A**<br><br>Intended Target:<br><br>**Unknown**	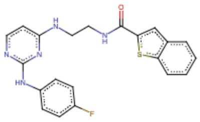	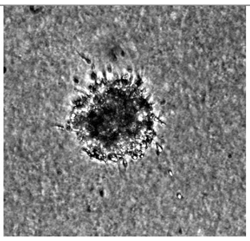	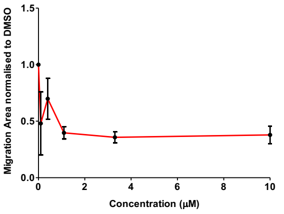

**GSK1379722A**<br><br>Intended Target:<br><br>**Unknown**	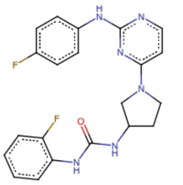	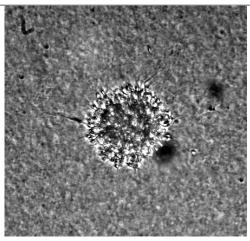	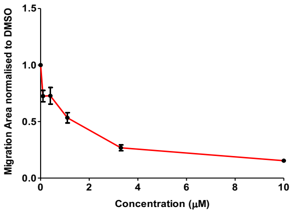
**GW560116X**<br><br>Intended Target:<br><br>**Unknown**	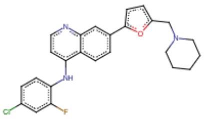	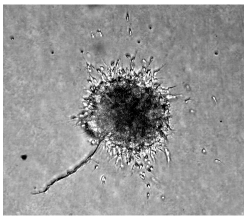	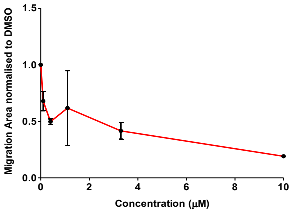
